# New Cross-Linked Polymeric Materials Modified with Antimicrobial Compounds in Relation to Their Biological Activities and Biodegradation by the Laccase-Producing Fungus *Cerrena unicolor*

**DOI:** 10.3390/biom16050731

**Published:** 2026-05-15

**Authors:** Karolina Kiełczewska-Klim, Dawid Stefaniuk, Marcin Grąz, Rafał Typek, Bożena Pawlikowska-Pawlęga, Anna Pawlik, Beata Podkościelna, Magdalena Jaszek

**Affiliations:** 1 Department of Polymer Chemistry, Maria Curie-Sklodowska University in Lublin, Gliniana 33, 20-614 Lublin, Poland; beata.podkoscielna@mail.umcs.pl; 2 Department of Biochemistry and Biotechnology, Institute of Biological Sciences, Maria Curie-Sklodowska University in Lublin, Akademicka 19, 20-033 Lublin, Poland; dawid.stefaniuk@mail.umcs.pl (D.S.); marcin.graz@mail.umcs.pl (M.G.); anna.pawlik@mail.umcs.pl (A.P.); 3 Department of Chromatography, Institute of Chemical Sciences, Faculty of Chemistry, Maria Curie-Sklodowska University in Lublin, Maria Curie-Sklodowska Square 3, 20-031 Lublin, Poland; rafal.typek@mail.umcs.pl; 4 Department of Functional Anatomy and Cytobiology, Institute of Biological Sciences, Maria Curie-Sklodowska University in Lublin, Akademicka 19, 20-033 Lublin, Poland; bozena.pawlikowska-pawlega@mail.umcs.pl

**Keywords:** cross-linked composites, zinc oxide, copper(II) sulfate, benzethonium chloride, nanosilver, fungi, laccase, biodegradation, SEM, profilometric analysis

## Abstract

This study characterizes novel cross-linked polymeric composites based on bisphenol A glycerolate dimethacrylate (BPA.DM) as the primary matrix, incorporating 1-vinyl-2-pyrrolidone (NVP) or 2-hydroxyethyl methacrylate (HEMA) as active diluents, and modified with antimicrobial agents: zinc oxide (ZnO), copper(II) sulfate (CuSO_4_), nanosilver (Ag), and benzethonium chloride (BEN). Release kinetics of active components into water and LH medium were measured over 20 days using HPLC (bisphenol A, benzethonium chloride), GF AAS (Cu, Zn, Ag), and GC–MS, revealing highest silver release from HEMA+Ag composites (1671 µg/L), substantial copper release from HEMA (354 mg/L) and NVP (319 mg/L) systems, while benzethonium chloride exhibited significantly lower migration. The effect of NVP- and HEMA-containing composites on the metabolism of the *Cerrena unicolor* was also assessed. Scanning electron microscopy (SEM) and optical profilometry confirmed extensive surface degradation by *C. unicolor* mycelium, manifesting as cracks, increased porosity, and altered surface across HEMA- and NVP-based composites after 21-day incubation. Biochemical analysis of the fungus post-culture liquids demonstrated that both composite types markedly enhanced extracellular laccase activity at all tested time points (7, 14, 21 days), with ethanol-sterilized samples inducing a slower-migrating laccase isoform identified via zymography. These materials also increased total protein concentration and superoxide anion radical levels while reducing phenolic compounds relative to controls. The findings demonstrate that antimicrobial-modified BPA.DM composites not only undergo controlled biodegradation by *C. unicolor* but crucially serve as potential laccase inducers, highlighting their dual utility in bioactive material design and fungal enzyme biotechnology.

## 1. Introduction

*Cerrena unicolor* is a white-rot basidiomycete wood-decaying fungus. It is recognized as either a parasite or a saprotroph that has the ability to decompose wood from many deciduous species and selected conifers. One of its biological traits is the production of several hydrolytic and oxidative enzymes that cause white rot. These include laccase, cellulase, lignin peroxidase, xylanase, and many others [1,2,3]. The available literature suggests that there are many more enzymes involved in wood degradation than have been identified so far. Many of the transcriptome analyses of this fungus conducted to date suggest that approximately 60 protein-coding transcripts are differentially regulated, depending on the type of wood substrate used. Among others, alcohol oxidase and peroxidase have been identified as enzymes responsible for lignin metabolism [4,5]. Previous studies have shown the possibility of modulating the metabolism of this fungus by introducing composites modified with lignin as fillers into culture media [6,7].

The most comprehensively described enzyme from the fungus *C. unicolor* is laccase [8,9,10,11]. It belongs to the oxidase group, which contains blue copper proteins among others, and is well known for its ability to oxidize aromatic and non-aromatic compounds, such as hydrocarbons and their breakdown products, by reducing molecular oxygen to water. The literature mainly describes the role of laccase as an enzyme for the biodegradation of lignocellulose, which has a very high resistance to biological and chemical degradation, making wood decomposition a time-consuming process [12,13,14]. Laccase is a multifunctional enzyme also used for water purification, biosensor construction, degradation of polymeric materials, or oxidative polymerization [15,16]. In addition, it has a proven application in bioremediation, as well as in the textile and food industries [17]. Numerous publications indicate the variety of biomedical applications of laccase isolated from the fungus *C. unicolor*, including its use to inhibit the reverse transcriptase activity of HIV and the proliferation of breast and cervical cancer cells [18,19].

Polymer-based composite materials are of great interest due to the potential to modify their physicochemical properties, as well as their wide range of applications. Their performance properties depend largely on the composition of the organic matrix and, in particular, on the type of monomers and modifiers used [20]. Bisphenol A glycerolate dimethacrylate (BPA.DM) is a common base monomer. It provides composite materials with high mechanical stability and is characterized by relatively low polymerization shrinkage [21]. However, its high viscosity necessitates the addition of comonomers and modifiers to enhance processability [22].

To minimize the risks associated with BPA.DM’s viscosity, hydrophilic comonomers such as 2-hydroxyethyl methacrylate (HEMA) and N-vinylpyrrolidone (NVP) are often introduced [23]. These compounds reduce viscosity and improve wettability, facilitating better dispersion and interaction within the composite matrix [24]. However, their hydrophilic nature can also increase water absorption and accelerate hydrolytic degradation, which may compromise long-term stability [25]. In addition to the polymer matrix, incorporating functional modifiers is important in altering the performance properties of composites [26]. Metal-based additives, such as silver (Ag), zinc oxide (ZnO) and copper sulfate (CuSO_4_), have well-documented antimicrobial properties, primarily due to their ability to generate reactive oxygen species and destroy microbial cell structures [27,28]. Similarly, quaternary ammonium compounds, such as benzethonium chloride, exhibit strong antimicrobial activity by destabilizing the cell membrane; however, their potential leaching remains a concern [29].

The main objective of this study was to evaluate the potential of using newly synthesized materials enriched with modifiers, such as Ag, ZnO, CuSO_4_ and BEN, to stimulate the biological activity of fungi in order to synthesize biotechnologically important enzymes, such as laccase, chitinase, β-glucosidases and proteases. The study also aimed to investigate the biodegradability of such composites by the fungus *Cerrena unicolor*. Scanning electron microscopy (SEM) and profilometric analysis were used to confirm changes in the structure of composite materials caused by fungal treatment and to determine the rate at which composite material components are released into the external medium as a sign of degradation. Other biochemical markers, such as phenolic compounds, superoxide anion radical levels, total sugar levels and protein concentrations, were also determined in the culture medium.

## 2. Materials and Methods

### 2.1. Synthesis of Composite Materials

#### 2.1.1. Materials and Chemicals

1-vinyl-2-pyrrolidone (NVP), 2-hydroxyethyl methacrylate (HEMA), bisphenol A glycerolate dimethacrylate (BPA.DM), zinc oxide, copper(II) sulfate, nanosilver, and 2,2-dimethoxy-2-phenylacetophenone (Irgacure 651, IQ) were obtained from Sigma-Aldrich (Taufkirchen, Germany). Benzethonium chloride was purchased from Lonzagard^®^ (Basel, Switzerland).

#### 2.1.2. Synthesis of Cross-Linked Composites

Composite materials containing bisphenol A glycerolate dimethacrylate (BPA.DM) as the main component were synthesized using UV polymerization. BPA.DM was mixed with one of two comonomers—N-vinylpyrrolidone (NVP) or 2-hydroxyethyl methacrylate (HEMA)—at a mass ratio of 7:3. The monomer mixtures were placed in a heating chamber at 65 °C to vent. Then, 10 wt% of a modifier (Ag, ZnO, CuSO_4_ or benzethonium chloride, BEN) relative to the total monomer mass was added to each formulation. Composites without any special additives were prepared as a control sample. A photoinitiator (Irgacure, IQ) was introduced at 3 wt% relative to the monomer content. The mixtures were thoroughly homogenized, and then transferred into glass moulds coated with a release agent and equipped with Teflon spacers. The moulds were placed in a chamber fitted with 160 W mercury lamps and irradiated for 30 min. To ensure complete curing, the samples were subsequently post-cured in a heating chamber at 85 °C for 4 h.

As a result, eight types of composite plate (70 × 100 mm, 2 mm thick) were obtained. The composites containing BEN were transparent, whereas those modified with CuSO_4_, ZnO or Ag were opaque. The process used to obtain the materials is described in the patent application [30].

### 2.2. Preparation of Experimental Cerrena unicolor Fungal Cultures

The *C. unicolor* strain (FCL 139, DQ056858) was obtained from the Fungal Culture Collection (FCL) of the Department of Biochemistry and Biotechnology (Maria Curie-Sklodowska University, Poland). The maternal culture was cultivated for 14 days at 25 °C in Lindeberg–Holm’s liquid medium containing the following components in specified concentrations: glucose 10 g/L, L-asparagine 1.5 g/L, MgSO_4_ × 7 H_2_O 0.5 g/L, KH_2_PO_4_ 0.47 g/L, Na_2_HPO_4_ × 12 H_2_O 0.48 g/L, yeast extract 0.1 g/L, Mn(CH_3_COO)_2_ × 4 H_2_O 12 mg/L, Zn(NO_3_)_2_ × 6 H_2_O 3.14 mg/L, CuSO_4_ × 5 H_2_O 3.9 mg/L, Ca(NO_3_)_2_ × 4 H_2_O 50 mg/L, FeCl_3_ × 6 H_2_O 3.2 mg/L, and thiamine 50 μg/L [31]. The maternal cultures, which had been homogenized in sterile conditions, were used to inoculate the experimental cultures. These were carried out in 25 mL Erlenmeyer flasks containing 10 mL of medium.

Preparation of polymer samples for the biological experiments: The composite samples were cut into 1 cm × 1 cm pieces. The first batch of composites was placed in a laminar chamber and exposed to UV light. The second batch was placed in a 70% ethanol solution for 3 min, thoroughly rinsed with sterile water and allowed to dry completely in the laminar chamber. Fragments of sterilized composite materials were placed in flasks containing the inoculum. For each composite variant, cultures were incubated for 7, 14 and 21 days at 28 °C under non-agitated conditions, in three replicates. After each incubation period (7, 14 and 21 days), the post-culture fluid was separated from the fungal biomass and polymer samples using Miracloth (Calbiochem, St. Louis, MO, USA) filters, frozen, and used for downstream biochemical analyses. Samples of the composite materials were rinsed thoroughly in sterile water and left for testing using a profilometer and SEM analyses.

### 2.3. Methods for Characterizing Composites

The composite samples were cut into 1 cm × 1 cm pieces. To avoid the risk of damaging the composite structure, while at the same time introducing sterile composite samples to the solutions, the composites were exposed to UV radiation in a laminar chamber [32,33].

#### 2.3.1. Release of Composite Material Components into the External Medium

To test for the release of components from the composite material over time, fragments of the composite material, prepared as described above, were placed in water and Lindeberg–Holm liquid medium [31] for 10 and 20 days. After this period, the amounts of components such as bisphenol A glycerolate dimethacrylate, copper(II) sulfate, nanosilver, benzethonium chloride, and zinc oxide released into the external medium were determined. The methods described below were used for this purpose.

The analysis of the bisphenol A glycerolate dimethacrylate concentration was performed using an HPLC system (Agilent Infinity 1260 equipped with DAD detector, Agilent Technologies, Santa Clara, CA, USA) fitted with a Phenomenex Luna column (150 mm × 4.6 mm, 5 µm) coupled to a DAD detector. Elution was carried out in the gradient mode from 50 to 90% of acetonitrile (ACN, eluent B) over 5 min. The eluent flow rate was maintained at 1 mL/min throughout the separation process, and the temperature of the column was maintained at 45 °C. Each 5 μL sample was injected using an autosampler. Detection was carried out at 210 nm. Agilent OpenLAB CDS ChemStation LC and CE Drivers (A.02.10 (026) version) software was used for data processing and reporting. Identification of the substrate peak was achieved by comparing retention times with the standard. A sample of the BPA.DM standard was prepared by dissolving it in a solution of methanol and water in a volume ratio of 1:1.

The Ag, Cu, and Zn concentration determination was performed by an atomic absorption spectrometer with atomization in a graphite furnace (GF AAS) and Zeeman background correction (SpectrAA 880Z, Varian, Belrose, Australia). The concentration level was determined at 328.1 nm for silver, 327.4 nm for copper, and 307.6 nm for zinc. The pyrolysis and atomization temperatures were 400 °C and 2200 °C for Ag, 800 °C and 2300 °C for Cu, and 300 °C and 1900 °C for Zn, respectively.

Quantitative analyses of benzethonium chloride were conducted using a gas chromatograph in combination with a triple quadruple tandem mass spectrometer detector (GCMS-TQ8040; Shimadzu, Kyoto, Japan). The GC–MS conditions were as follows: capillary column—Zebron ZB5-MSi (30 m × 0.25 mm i.d., 0.25 μm film thickness; Phenomenex, Torrance, CA, USA); carrier gas: helium (grade 5.0); flow rate: 1.0 mL/min; splitless/split injection mode (sampling time: 1.00 min); glass-wool-packed liner (AG0-4683, Phenomenex): —3.4 mm ID × 95 mm L × 5 mm OD; injector temperature: 310 °C; injection volume: 1 μL; temperature program: initial temperature 60 °C held for 1 min, followed by an increase to 310 °C at a rate of 15 °C/min. The final temperature was held for 5 min. Mass spectrometer parameters: normalized electron energy of 70 eV; ion source temperature: 225 °C. The SIM mode for *m*/*z* = 412 was used. To determine the concentration of benzethonium chloride in the tested samples, a calibration curve was prepared using the benzethonium chloride solution prior to the analysis.

For each analysis, each sample was analyzed three times (*n* = 3), and the obtained concentrations are the averages of these analyses.

#### 2.3.2. Scanning Electron Microscopy (SEM)

The composite samples, which had previously been exposed to the growing mycelium of *C. unicolor*, and those conducted to the release experiment, were cut into rectangles with an approximate surface area of 64 mm^2^ each and then mounted onto stubs. Next, the samples were coated with gold in an Emitech K550X Sputter Coater (Emitech, Kent, UK). Afterwards, the samples were imaged using a TESCAN Vega 3 LMU microscope (TESCAN Group, Brno, Czech Republic) in the secondary electron mode. Pictures were collected at the following magnifications: ×200, 500, 1000, 2000, and 5000.

#### 2.3.3. Surface Roughness Visualization

The morphological images and surface roughness measurements of the composite fragments, which had been treated with the fungus and subjected to the release experiment, were obtained using an optical profilometer (Contour GT-K1, Veeco, Plainview, NY, USA) with very high accuracy in the size range from nanometers to 10 mm. Each sample was measured 3 times, and the average roughness (Ra) was also determined.

### 2.4. Biochemical Analysis of Fungal Culture Fluids

#### 2.4.1. Spectrophotometric Detection of Phenolic Compounds, Total Carbohydrates, Laccase Activity, Free Radicals, Antioxidant Properties, and Proteins

Biochemical analysis was performed on the post-culture liquid obtained from 7-, 14- and 21-day cultures of *C. unicolor* in the presence of HEMA- and NVP-based composites, in order to determine the effect of the composites on the fungus’s metabolism.

Laccase activity was determined using syringaldazine (4-hydroxy-3,5-dimethoxybenzaldehyde) as the enzyme substrate. During the reaction, the substrate is oxidized and the resulting pink product is measured at 525 nm in a pH 5.2 buffer. The activity is expressed in nkat/L [34]. The protein concentration was determined using a Coomassie Brilliant Blue (G250) solution according to the Bradford method. The measurements were performed at 595 nm. The results are expressed in µg/mL [35]. The phenolic compound content was determined using diazosulfanilamide (DASA method) and measured at a wavelength of 500 nm. The results are expressed in mM [36]. The relative level of superoxide anion radicals (SOR) was determined using nitrotetrazolium blue (NBT). The use of a 1 M NaOH solution prevented the precipitation of dark blue formazan. The measurements were performed at 560 nm. The SOR level was expressed as a percentage relative to the control, i.e., 7-day fungal post-culture liquid without composite addition, which was set as 100% [37]. The antioxidant properties were determined using the ABTS method, which tested the scavenging ability. The measurements were performed at a wavelength of 734 nm [18,38]. The total carbohydrates concentration was determined using a phenol–sulfuric acid method. The spectrophotometric measurements were performed at 490 nm and the results are expressed in µg/mL [39]. All measurements were performed in triplicate in three independent biological replications. All results are expressed as the mean ± SD (standard deviation) from three measurements (*n* = 3).

#### 2.4.2. Zymographic Detection of Laccase, Protease, and β-Glucosidase Activities Using Polyacrylamide Gel Electrophoresis

The culture fluid, which had been mixed with the sample buffer, was applied to the gel, prepared according to the Laemmli method [40]. To detect protease activity, an additional 1% gelatin solution was added to the gel. All zymographic separations were carried out at 4 °C and 145 V. The original electrophoresis gel can be found at Supplementary Materials.

##### Protease Activity Detection

Following separation, the gels were transferred to a citrate–phosphate buffer (pH 3.5 or 8.0) [18] and incubated for 18 h at 37 °C. Bands indicative of proteolytic activity were visualized using Coomassie Brilliant Blue G-250, as described in the procedure. The gels were archived using the Syngene G-Box system (Syngene, Baltimore, MD, USA).

##### β-Glucosidase Activity Detection

The three-step procedure for detecting β-glucosidase activity was employed, as described by [41] with modifications [42]. 4-Methylumbelliferyl β-D-glucopyranoside (MUG) was used as the fluorescent enzymatic substrate. During the first step, the gels were placed in a solution of citrate–phosphate buffer with a pH of 4.8. The gels were incubated for 30 min at 25 °C using a laboratory cradle. They were then transferred to a solution of the same buffer containing 5 µM MUG and incubated for a further 30 min. Finally, the gels were again rinsed in the buffer solution. Bands corresponding to enzyme activity were visualized using a transilluminator and archived (Syngene G-Box system, Syngene, Baltimore, MD, USA).

##### Laccase Activity Detection

To determine laccase activity, the gels were incubated in citrate–phosphate buffer at pH of 5.2 containing guaiacol as the substrate. The brown-red bands that corresponded to laccase activity were visualized using visible light and archived with the Syngene G-Box system.

### 2.5. Statistical Analysis

The statistical analysis of the results was performed using a two-way analysis of variance (two-way ANOVA), followed by Dunnett’s post hoc test with the control as reference. The significance level was set at *p* < 0.05.

For the formulations evaluated in the release test and for the anion radical levels, due to the lack of normality in data distribution (Shapiro–Wilk test, *p* < 0.001), the nonparametric aligned rank transform (ART) procedure was applied. The significance of the main effects and their interactions was then assessed using analysis of variance. Post hoc multiple comparisons were performed using Dunnett’s test (for anion radical levels) and Tukey’s test, comparing the tested formulations within each day. The analyses were performed using Statistica software version 13 (TIBCO Software Inc., San Ramon, CA, USA).

## 3. Results

### 3.1. Release of Composite Material Components into the External Medium

The concentration of zinc in the solution was determined for each of the composite samples. For each component of the composite, comparisons were made within a day. As previously described, the composites were placed in two different liquids—LH medium and deionized water. The zinc concentration was determined in each sample after 10 and 20 days (Table 1). It was observed that the concentration varied depending on the external medium in which the composite fragments were placed. The most intense release of zinc was observed in the case of water. For the NVP+ZnO composite, the concentrations were 5.75 ± 0.03 mg/L after 20 days and for HEMA+ZnO composite 7.67 ± 0.01 mg/L. The lowest zinc concentrations were found in NVP+ZnO water/10d (2.37 ± 0.00 mg/L), NVP+ZnO LH/10d, and HEMA+ZnO LH/10d (both at 2.62 ± 0.01 mg/L).

In the case of copper, the concentration determined after the release process was not strictly dependent on the external medium in which the samples were incubated (Table 2). The lowest copper concentration was found in the NVP+CuSO_4_ composite placed in LH medium (21.13 ± 0.07 mg/L) and HEMA+CuSO_4_ composite placed in water (22.49 ± 0.02 mg/L after 10 days and 23.80 ± 0.64 mg/L after 20 days). The highest concentrations of copper were obtained for NVP+CuSO_4_ in water after 20 days (319.07 ± 1.12 mg/L) and for HEMA+CuSO_4_ in the LH medium after 20 days (354.98 ± 3.75 mg/L). The most significant increase in the copper concentration between 10 and 20 days was also observed for these composites.

As with copper, the concentration of silver detected in the external medium was independent of the medium type (Table 3). The lowest nanosilver concentrations detected in the collected liquid were 27.54 ± 2.24 µg/L (HEMA+Ag LH/10d) and 32.22 ± 0.75 µg/L (HEMA+Ag LH/20d). Significant differences in silver concentrations were observed between days 10 and 20 of the release process, as determined for NVP+Ag water (an increase from 150.20 ± 4.65 µg/L to 413.88 ± 2.45 µg/L) and for HEMA+Ag water (an increase from 428.43 ± 3.26 µg/L to 1671.30 ± 5.12 µg/L).

The concentration of benzethonium chloride (BEN) in the external liquid following the release process depended on the composite type (Table 4). The lowest BEN content was found in HEMA+BEN LH at 0.23 ± 0.00 ppm after 10 days and 0.27 ± 0.00 ppm after 20 days. The highest values, which differed significantly from those of the other samples, were found in the NVP+BEN (water) sample: 22.00 ± 0.03 ppm after 10 days, and 23.13 ± 0.02 ppm after 20 days.

For composites containing no special additives with antimicrobial properties, the concentration of bisphenol A glycerolate dimethacrylate released to the external medium after 10 and 20 days was determined (Table 5). For the material containing NVP as the active diluent, no BPA.DM was detected in the sample after 10 or 20 days in water or LH medium. The highest concentrations found were 1.20 ± 0.01 µg/L and 1.40 ± 0.01 µg/L for HEMA LH at 10 and 20 days, respectively. Lower concentrations were found for HEMA water/10d (0.30 ± 0.02 µg/L) and for HEMA water/20d (0.30 ± 0.01 µg/L).

As indicated by the results presented above, the lowest concentrations released into the external medium were recorded for bisphenol A glycerolate dimethacrylate, benzethonium chloride, and silver ions. No clear relationship was observed between the type of external medium and the rate at which components were released from the composite.

### 3.2. Scanning Electron Microscopy of the Surface of the New Cross-Linked Composites That Were Subjected to a Release Experiment and Biodegradation by C. unicolor

Composite materials that had been placed in flasks with the *C. unicolor* culture for 21 days (materials after sterilization with UV and ethanol) and composites that had undergone analysis of the release of composite components into the external medium (i.e., the LH medium and water) for 20 days were selected for SEM studies. Non-sterilized materials that were not in contact with the culture medium, water, or microorganisms were used as control samples. Tests were performed on composites without any modifier, and on materials containing benzethonium chloride (BEN), which gave the most promising results of the biochemical tests.

**Figure 1:**
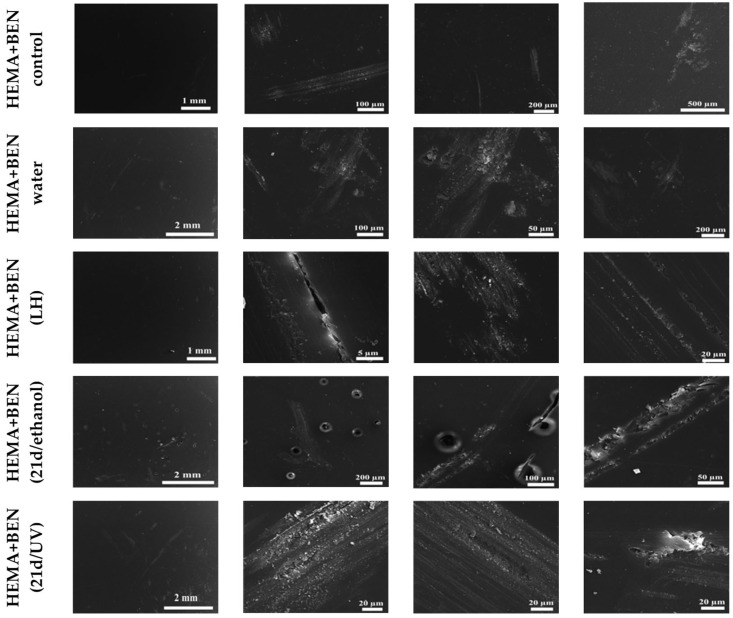
Evaluation of structural alterations in HEMA-containing composites based on SEM analysis. Non-sterilized materials that were not in contact with the culture medium, water, or microorganisms were used as a control (HEMA+BEN control). Samples after the release experiment (HEMA+BEN (water), HEMA+BEN (LH), and in those sterilized with UV or ethanol and subjected to 21-day biodegradation by *C. unicolor* (HEMA+BEN (21d/ethanol), HEMA+BEN (21d/UV)) were also analyzed.

The interactions between the UV- or ethanol-sterilized polymer composites and the *C. unicolor* were analyzed to verify the biodegradation properties of the fungus in relation to the untreated composite surface. No changes were observed on the surface of the non-sterilized HEMA+BEN composite (control), which had not been exposed to a culture medium, water, or microorganisms (Figure 1). In the composite materials analyzed after the release of their components into external media, the HEMA+BEN (water) sample showed changes on approximately three-quarters of the surface, with the main alterations being cracks in the recesses. The surface of the HEMA+BEN (LH) composite in the altered regions (a very tiny area) exhibited longitudinal cracks together with some material adhesion. However, significant changes were observed in the surface of the tested composites when HEMA-and BEN-containing materials sterilized with UV light and ethanol were incubated for 21 days in the presence of *C. unicolor*. Numerous indentations with cracks near round lesions covering the entire surface of the composite were observed in the case of HEMA+BEN (21d/ethanol). Approximately three-quarters of the surface was altered in the case of HEMA+BEN (21d/UV). Some indentations with material adhesion were easily noted.

In contrast, the surface of the HEMA composite not modified with benzethonium chloride [the HEMA (control)] was slightly changed, which was accompanied by longitudinal scratches without cracks, but with indentations (Supplementary Materials Figure S1). Most of the HEMA (water) composite sample surfaces were smooth and intact. However, the very small composite surface was altered, as scratches visible without cracks and an intersecting fiber structure was noted at the edges. The small surface area was changed in the case of HEMA (LH) sample, with longitudinal scratches without cracks, but with a few dents, present in the altered area. Almost the entire surface was changed in the case of HEMA (21d/ethanol). Different-sized indentations and cracks were visible. In the HEMA (21d/UV) composite, a large area of lesions was noted, with transverse and longitudinal cracks and accompanying invaginations.

**Figure 2:**
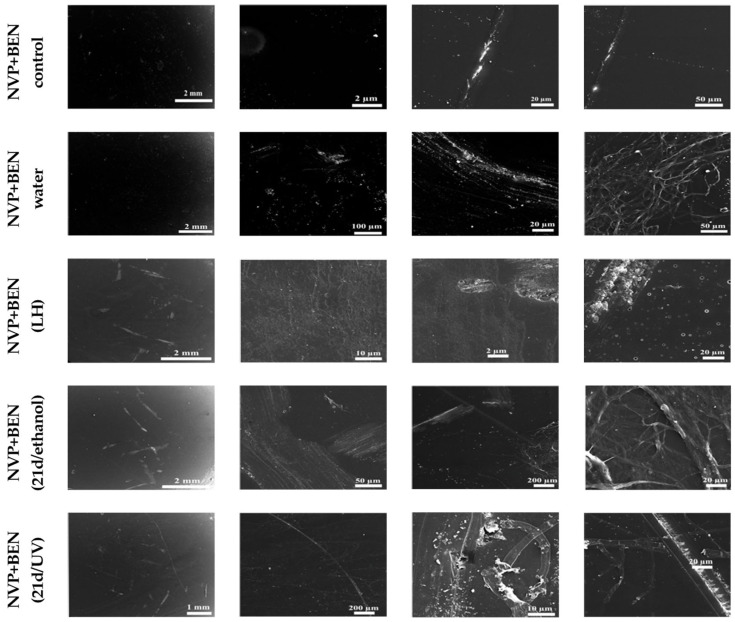
Evaluation of structural alterations in NVP-containing composites based on SEM analysis. Non-sterilized materials that were not in contact with the culture medium, water, or microorganisms were used as a control (NVP+BEN control). Samples after the release experiment (NVP+BEN (water), NVP+BEN (LH), and in those sterilized with UV or ethanol and subjected to 21-day biodegradation by *C. unicolor* (NVP+BEN (21d/ethanol), NVP+BEN (21d/UV)) were also analyzed.

The surface of the benzethonium chloride-modified, non-sterilized NVP+BEN (control) composite, which had not been exposed to culture medium, water, or microorganisms, remained largely unchanged (Figure 2). The composite surface was mostly altered in the case of NVP+BEN (water) and NVP+BEN (LH), i.e., the NVP-based composites that were subjected to the release experiment. These samples were characterized by a small crack, material adhesion, and round changes resembling holes. Scratches without cracks and intersecting fiber structures at the edges were visible in NVP+BEN (21d/ethanol) samples incubated in the presence of *C. unicolor* mycelium. Similarly, scratches and a few small cracks were visible in a few places on the surface. Additionally, an intersecting fiber structure was noticed at the edges. Most surfaces were altered, and longitudinal cracks and changes resembling intersecting fibers were observed in NVP (21d/UV).

The NVP (control) sample, lacking benzethonium chloride, exhibited an almost intact surface, with only minor scratches visible (Supplementary Materials Figure S2). Numerous bursts and depressions were sometimes observed in the case of NVP (water), where the composite surface was entirely covered with scratches. In the NVP (LH) composite, the surface was also scratched with characteristic long cracks. The NVP (21d/ethanol) sample incubated in the presence of *C. unicolor* mycelium exhibited noticeable cracks and depressions with material adhesion and a small area of changes. In the NVP (21d/UV) sample, incubated under the same conditions, less than half of the total composite surface was covered by numerous scratches and cracks without adhesion.

### 3.3. Surface Roughness of the New Cross-Linked Composites After C. unicolor Treatment

**Figure 3:**
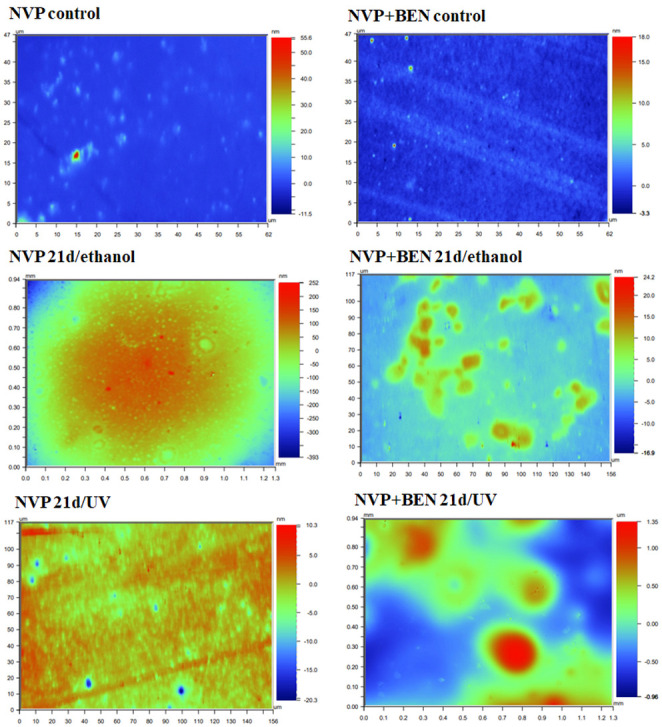
Profilometric analysis of the surface of NVP-containing composites that were subjected to a release experiment for 20 days and 21 days biodegradation by *C. unicolor*. The scale of the changes is illustrated by the change in color intensity. The presence of blue, dark blue, and red fragments confirms the changes to the surface of the composite samples. The small differently colored dots indicate punctual surface degradation caused by microorganisms.

**Figure 4:**
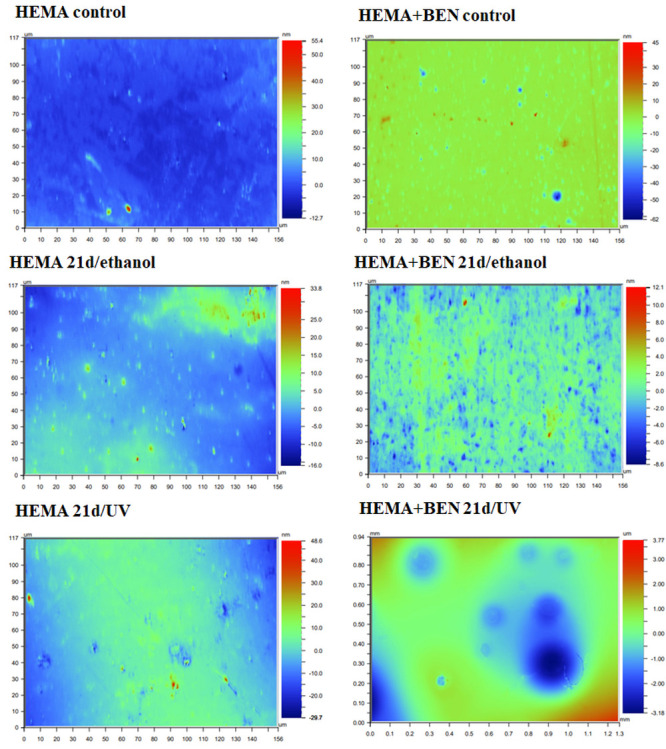
Profilometric analysis of the surface of HEMA-containing composites that were subjected to a release experiment for 20 days and 21 days biodegradation by *C. unicolor*. The scale of the changes is illustrated by the change in color intensity. The presence of blue, dark blue, and red fragments confirms the changes to the surface of the composite samples. The small differently colored dots indicate punctual surface degradation caused by microorganisms.

A wide profilometric analysis of the composite materials’ surface was also performed for NVP- and HEMA-containing composites without any modifier, and on materials modified with benzethonium chloride (BEN), which gave the most promising results of the biochemical tests. As with the SEM analysis, this included a control material—a polymer that had not come into contact with the *C. unicolor* fungus and samples that had been treated with UV light (21d/UV) or 70% ethanol (21d/ethanol) and then incubated with a *C. unicolor* culture for 21 days. The analyses revealed quite significant changes to the surface of the composite samples treated with metabolites produced by *C. unicolor*. For all the composites, an increase in the Ra value, compared with the control sample, was evident. The composite samples with the largest increases in surface roughness were observed for the HEMA+BEN 21d/UV composite (an increase from 0.92 nm to 719.50 nm compared to the control sample) and the NVP+BEN 21d/UV composite (an increase from 0.54 nm to 348.54 nm compared to the control sample). The Ra data are summarized in Table 6. Figure 3 and Figure 4 show the profilometric analyses of the samples.

### 3.4. Spectrophotometric Assessments of Chosen Biochemical Parameters in C. unicolor Cultures Caused by the Presence of Composite Materials

**Figure 5:**
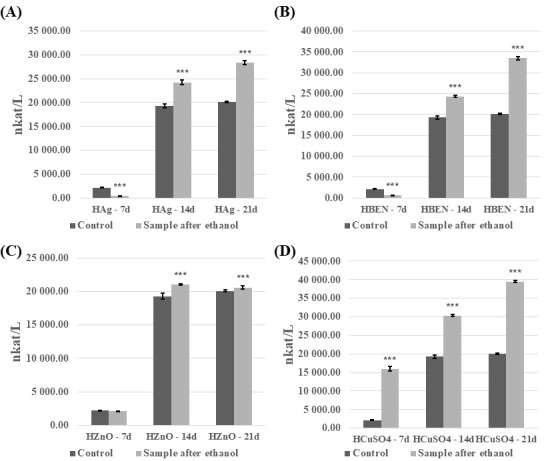
Analysis of extracellular laccase activity after cultivation of the fungus *C. unicolor* for 7, 14 or 21 days in the presence of HEMA-containing composite material fragments, following ethanol disinfection. (**A**)—samples with nanosilver; (**B**)—samples with benzethonium chloride; (**C**)—samples with zinc oxide; (**D**)—samples with copper(II) sulfate; bars represent means with error bars denoting standard deviation (SD) from three measurements (*n* = 3). Significance against control determined by Dunnett’s test, where the *p* value is *** 0.001.

**Figure 6:**
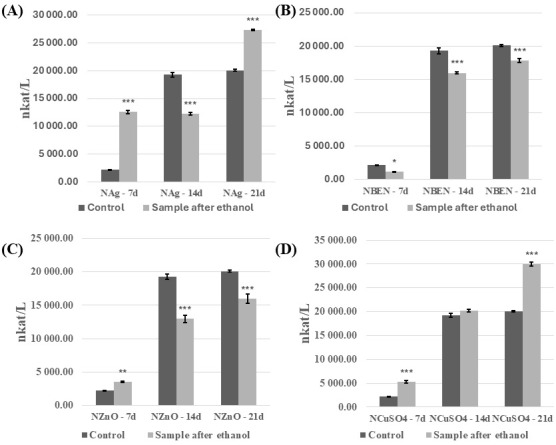
Analysis of extracellular laccase activity after cultivation of the fungus *C. unicolor* for 7, 14 or 21 days in the presence of NVP-containing composite material fragments, following ethanol disinfection. (**A**)—samples with nanosilver; (**B**)—samples with benzethonium chloride; (**C**)—samples with zinc oxide; (**D**)—samples with copper(II) sulfate; bars represent means with error bars denoting standard deviation (SD) from three measurements (*n* = 3). Significance against control determined by Dunnett’s test, where the *p* value is *** 0.001; ** 0.01; * 0.05.

The analysis revealed that introducing the modified, cross-linked polymeric material into the *C. unicolor* culture resulted in significant changes to the biochemical parameters examined. These changes depended on the method used to disinfect the composite samples and on the growth phase of the fungal culture. The ethanol-sterilized composites showed the most pronounced differences and are therefore discussed in more detail in this study. In general, it was evident that the composites increased laccase activity after disinfection with ethanol (Figure 5 and Figure 6). An increase in laccase activity was observed in most samples, in comparison to the control. Exceptions included HEMA+Ag on day 7 of culture, and in the HEMA-BEN/7d, HEMA-ZnO/7d, NVP/14d, NVP-Ag/14d, NVP-BEN/7d, and NVP-ZnO on days 14 and 21 of culture. The highest laccase activity was found in HEMA+CuSO_4_ on day 21 of culture (39,475.3 nkat/L), and the lowest in HEMA-Ag on day 7 of culture (464.42 nkat/L). The presence of composites containing modifier additives and the control samples was found to decrease laccase activity in the UV-sterilized samples (Figures S3 and S4). However, an exception was the addition of benzethonium chloride and copper(II) sulfate to the composite with HEMA on day 21 of culture, which resulted in an increase in laccase activity from 7656.65 nkat/L to 9106.33 nkat/L and 7656.65 nkat/L to 9494.22 nkat/L, respectively. A similar trend was observed with the addition of copper(II) sulfate to the composite with NVP on day 14 of culture (4708.55 nkat/L for the control sample and 10,368.73 nkat/L for sample with special additive), and 21 day of culture (7656.76 nkat/L for control and 9202.30 nkat/L for sample with copper(II) sulfate).

**Figure 7:**
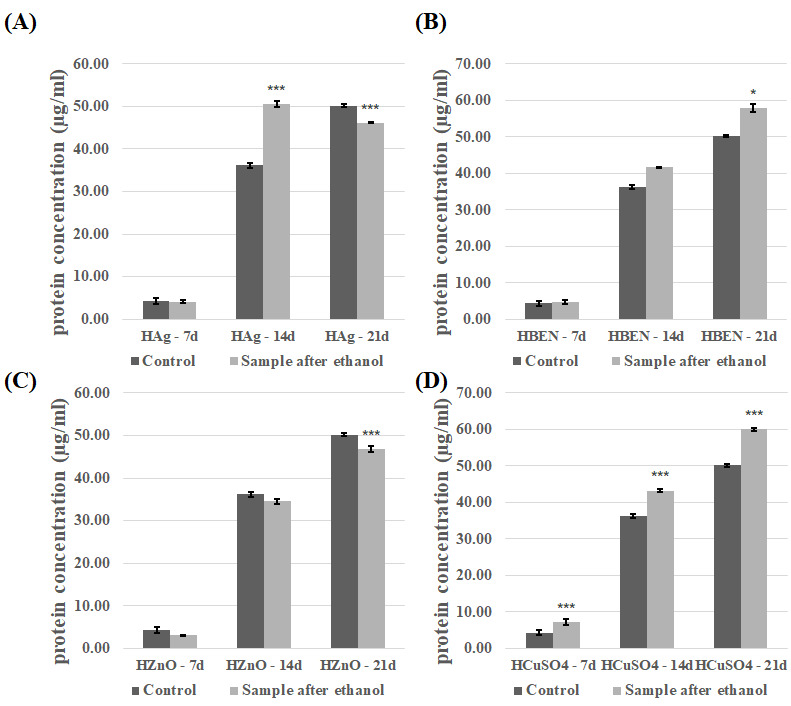
Concentration of proteins in the culture fluid of *C. unicolor* after 7, 14 and 21 days of cultivation in the presence of HEMA-containing composite material fragments, following ethanol disinfection. (**A**)—samples with nanosilver; (**B**)—samples with benzethonium chloride; (**C**)—samples with zinc oxide; (**D**)—samples with copper(II) sulfate; bars represent means with error bars denoting standard deviation (SD) from three measurements (*n* = 3). Significance against control determined by Dunnett’s test, where the *p* value is *** 0.001; * 0.05.

**Figure 8:**
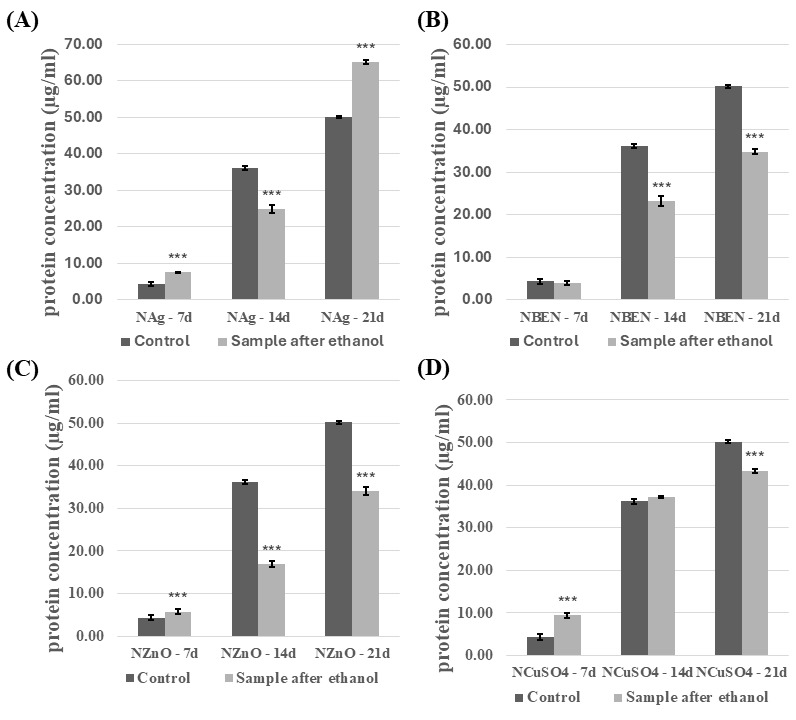
Concentration of proteins in the culture fluid of *C. unicolor* after 7, 14 and 21 days of cultivation in the presence of NVP-containing composite material fragments, following ethanol disinfection. (**A**)—samples with nanosilver; (**B**)—samples with benzethonium chloride; (**C**)—samples with zinc oxide; (**D**)—samples with copper(II) sulfate; bars represent means with error bars denoting standard deviation (SD) from three measurements (*n* = 3). Significance against control determined by Dunnett’s test, where the *p* value is *** 0.001.

The introduction of the modified composites into the experimental fungal cultures also affected concentration of the extracellular proteins. The effect of the presence of composites disinfected with ethanol was analyzed (Figure 7 and Figure 8). The presence of composites containing HEMA-BEN, HEMA+CuSO_4_, and NVP+CuSO_4_ resulted in an increase in the protein concentration relative to the control samples, regardless of the growth phase. The highest protein concentrations were recorded on day 21 of incubation: 59.99 ± 0.50 µg/mL for the HEMA+CuSO_4_ samples and 65.18 ± 0.67 µg/mL for the NVP+Ag samples. By contrast, the lowest protein concentrations were measured on day 7 for both the control and test samples, ranging from 7.20 ± 0.82 to 9.42 ± 0.58 µg/mL for HEMA+CuSO_4_ and NVP+CuSO_4_, respectively. For the other composites, however, no clear relationship between the growth phase and the effect of the composite on the protein concentration could be shown. In cultures performed in the presence of HEMA-containing composites after UV sterilization, an increase in protein concentration was observed, compared to the control samples (Figure S5). The exceptions were HEMA/14d, HEMA-Ag/14d, HEMA-BEN/14d, and HEMA+CuSO_4_/14d. The highest protein concentration and the greatest increase were observed for HEMA-Ag after 21 days of culture. A different trend was observed for the NVP-containing composites after UV sterilization (Figure S6). In this case, the presence of the composites resulted in a decrease in the protein concentration. The exceptions were the cultures after seven days of incubation (an increase compared with the control) and NVP+CuSO_4_/14d.

**Figure 9:**
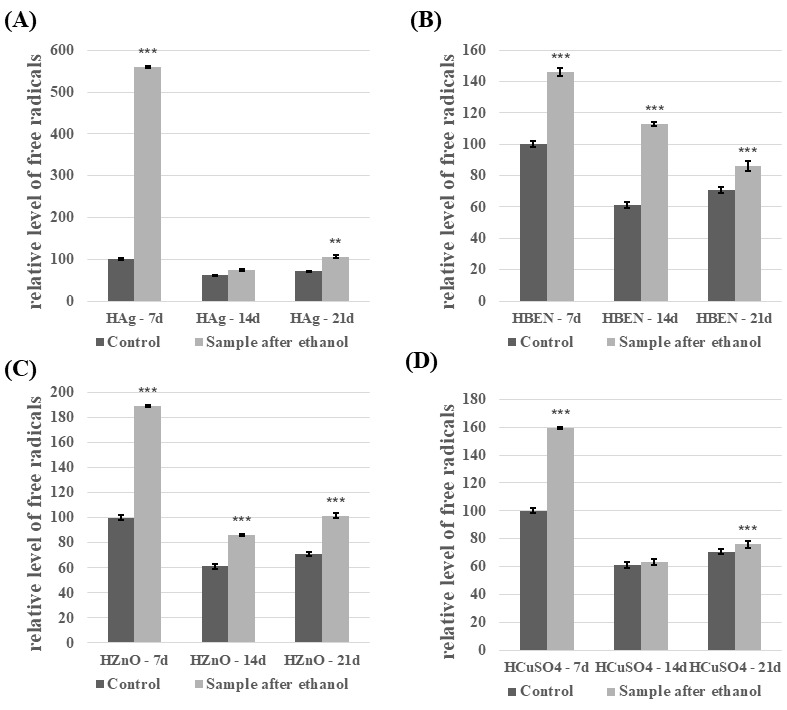
Relative level of superoxide anion radicals—analysis conducted for HEMA-containing composites after ethanol disinfection. (**A**)—samples with nanosilver; (**B**)—samples with benzethonium chloride; (**C**)—samples with zinc oxide; (**D**)—samples with copper(II) sulfate; bars represent means with error bars denoting standard deviation (SD) from three measurements (*n* = 3). Significance against control determined by Dunnett’s test, where the *p* value is *** 0.001; ** 0.01.

**Figure 10:**
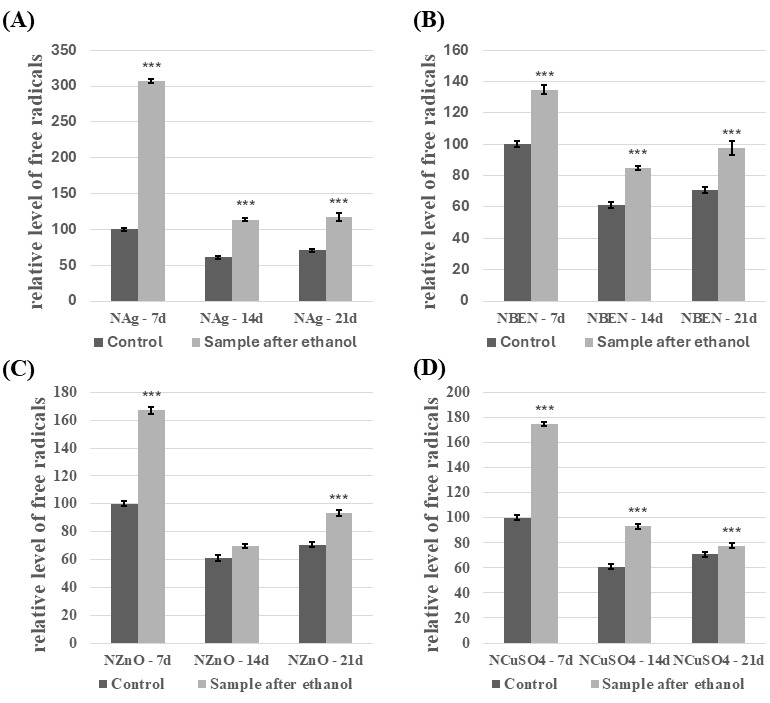
Relative level of superoxide anion radicals—analysis conducted for NVP-containing composites after ethanol disinfection. (**A**)—samples with nanosilver; (**B**)—samples with benzethonium chloride; (**C**)—samples with zinc oxide; (**D**)—samples with copper(II) sulfate; bars represent means with error bars denoting standard deviation (SD) from three measurements (*n* = 3). Significance against control determined by Dunnett’s test, where the *p* value is *** 0.001.

Compared to the control, *C. unicolor* cultures performed in the presence of NVP- and HEMA-containing composites after disinfection with ethanol showed an increase in the free radical concentration (Figure 9 and Figure 10). The highest level of SOR was detected in both HEMA+Ag/7d (559.5% relative to the control) and in NVP+Ag/7d (206.9% relative to the control). For the experimental cultures containing UV-sterilized HEMA composites, an increase in free radicals’ concentration was observed in most experimental cases compared to the control samples (Figure S7). The exceptions were HEMA/21d and HEMA+CuSO_4_/21d. A very similar relationship was observed for cultures performed in the presence of UV-sterilized NVP composites (Figure S8). Only NVP/21d and NVP-ZnO/21d showed a decrease in the free radical concentration, compared to the control.

**Figure 11:**
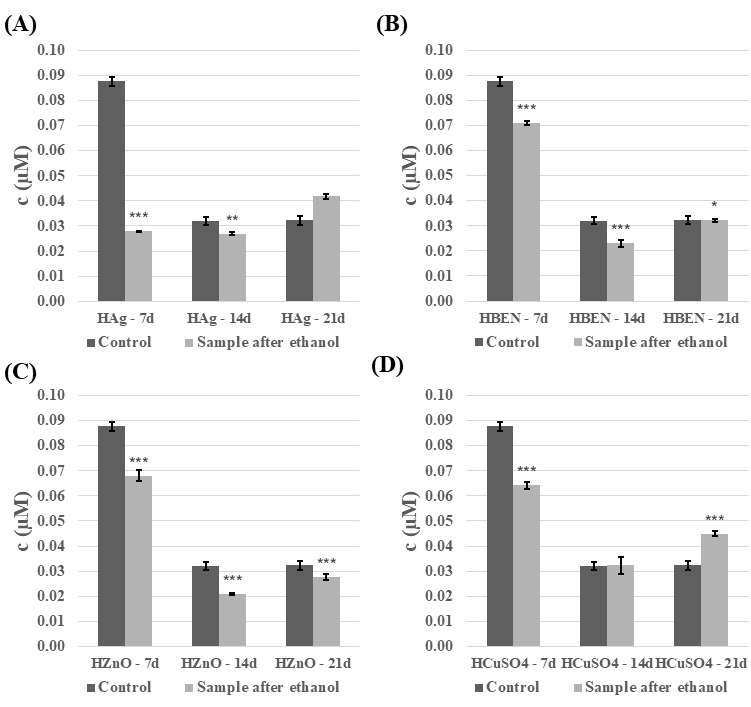
Phenolic compounds content in the culture fluid of *C. unicolor* after 7, 14 and 21 days of cultivation in the presence of HEMA-containing composite material fragments, following ethanol disinfection. (**A**)—samples with nanosilver; (**B**)—samples with benzethonium chloride; (**C**)—samples with zinc oxide; (**D**)—samples with copper(II) sulfate; bars represent means with error bars denoting standard deviation (SD) from three measurements (*n* = 3). Significance against control determined by Dunnett’s test, where the *p* value is *** 0.001; ** 0.01; * 0.05.

**Figure 12:**
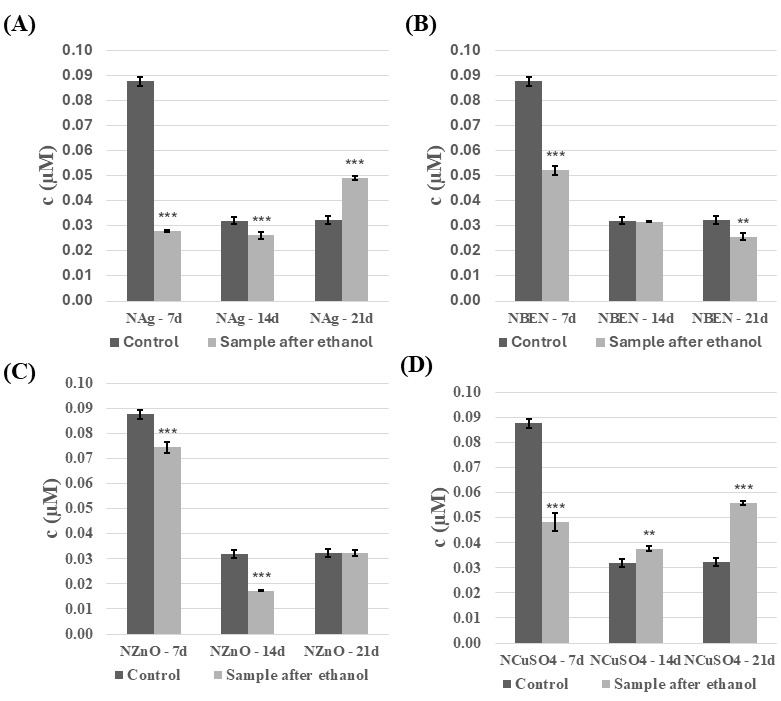
Phenolic compounds content in the culture fluid of *C. unicolor* after 7, 14 and 21 days of cultivation in the presence of NVP-containing composite material fragments, following ethanol disinfection. (**A**)—samples with nanosilver; (**B**)—samples with benzethonium chloride; (**C**)—samples with zinc oxide; (**D**)—samples with copper(II) sulfate; bars represent means with error bars denoting standard deviation (SD) from three measurements (*n* = 3). Significance against control determined by Dunnett’s test, where the *p* value is *** 0.001; ** 0.01.

The next step was to analyze the phenolic compound content of the *C. unicolor* culture fluid. In general, the presence of ethanol-disinfected composite samples resulted in a decrease in phenolic compounds content, and this decrease was also evident as the duration of the culture increased (Figure 11 and Figure 12). The highest concentrations of phenolic compounds were recorded in the culture fluid of the control samples on day 7 of incubation, reaching 0.74 µM for the NVP+ZnO sample. However, a reversal in this trend was observed for both composites containing Ag and Cu on day 21 of incubation, and for the NVP+CuSO_4_/14d sample. A very similar relationship was observed for composites treated with UV light (Supplementary Materials Figures S9 and S10).

**Figure 13:**
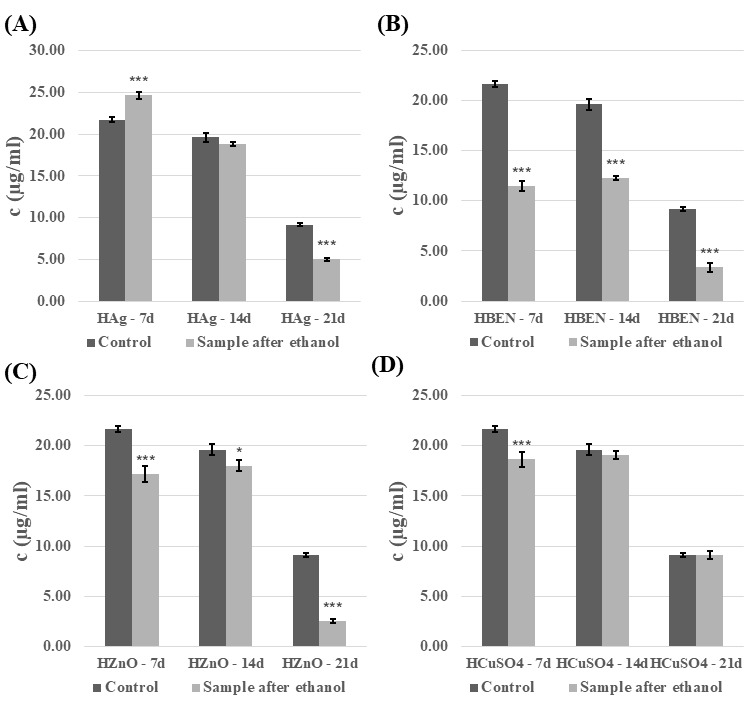
Extracellular total carbohydrates content in the culture fluid of *C. unicolor* after 7, 14 and 21 days of cultivation in the presence of HEMA-containing composite material fragments, following ethanol disinfection. (**A**)—samples with nanosilver; (**B**)—samples with benzethonium chloride; (**C**)—samples with zinc oxide; (**D**)—samples with copper(II) sulfate; bars represent means with error bars denoting standard deviation (SD) from three measurements (*n* = 3). Significance against control determined by Dunnett’s test, where the *p* value is *** 0.001; * 0.05.

**Figure 14:**
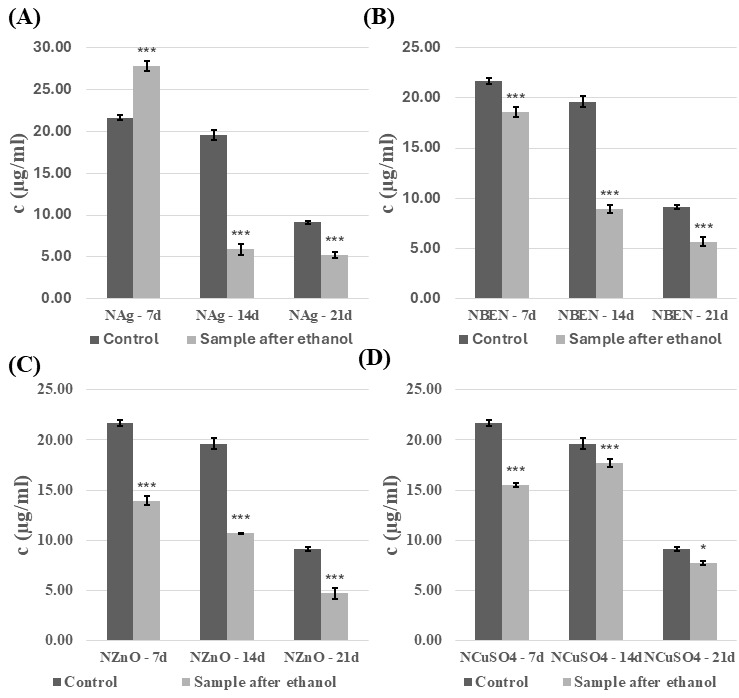
Extracellular total carbohydrates content in the culture fluid of *C. unicolor* after 7, 14 and 21 days of cultivation in the presence of NVP-containing composite material fragments, following ethanol disinfection. (**A**)—samples with nanosilver; (**B**)—samples with benzethonium chloride; (**C**)—samples with zinc oxide; (**D**)—samples with copper(II) sulfate; bars represent means with error bars denoting standard deviation (SD) from three measurements (*n* = 3). Significance against control determined by Dunnett’s test, where the *p* value is *** 0.001; * 0.05.

The total sugar content in the *C. unicolor* culture medium changed over time and depended on the material used. Disinfecting the composites in a 70% ethanol solution generally resulted in a lower concentration of sugars in the culture fluid than in the control sample. However, after seven days, HEMA-Ag and NVP-Ag were found to be the exceptions, with the highest concentrations of phenolic compounds at 24.6–27.8 µg/mL (Figure 13 and Figure 14). The presence of the UV-sterilized composites generally increased the total sugar level, particularly during the initial stages of cultivation (Figures S11 and S12). A decrease was observed for the majority of HEMA and NVP-based composites after 14 and 21 days of cultivation.

### 3.5. Zymographic Analysis of Enzyme Activities (Laccase, Protease, and β-Glucosidase)

**Figure 15:**
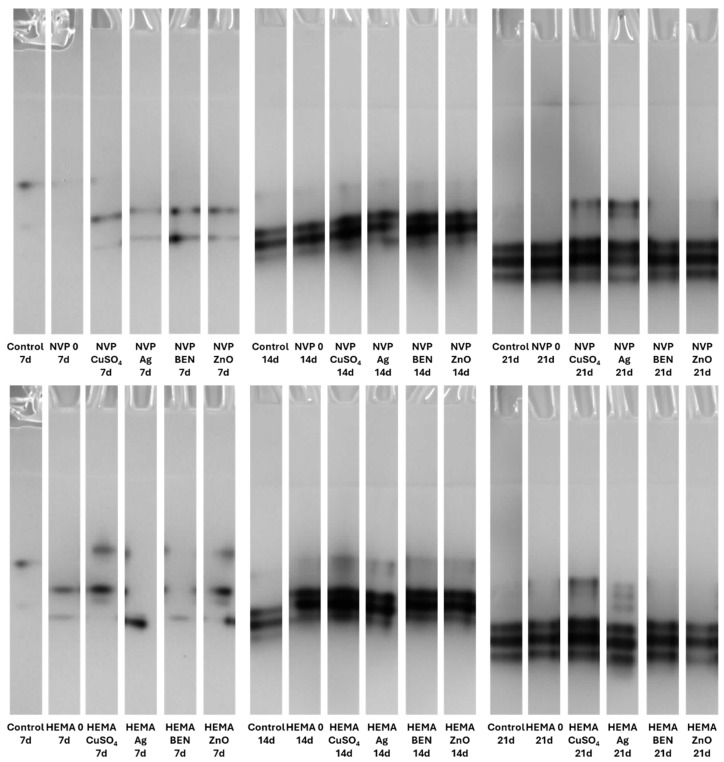
Zymographic detection of extracellular laccase activity in the culture fluid of *C. unicolor* after 7, 14 and 21 days of cultivation in the presence of NVP- and HEMA-containing composite material fragments, following ethanol disinfection.

Electrophoretic analysis of the enzyme activities in the post-culture fluid revealed significant changes in the laccase and β-glucosidase activity profiles after UV sterilization (Figures S13–S16). The zymograms obtained clearly demonstrate the presence of bands indicating laccase activity. It is worth emphasizing that both NVP- and HEMA-containing polymers, with the addition of copper ions and nanosilver, when sterilized with ethanol, induced the formation of an additional activity band corresponding to the slower migrating laccase isoform on the 21th day of incubation (Figure 15).

**Figure 16:**
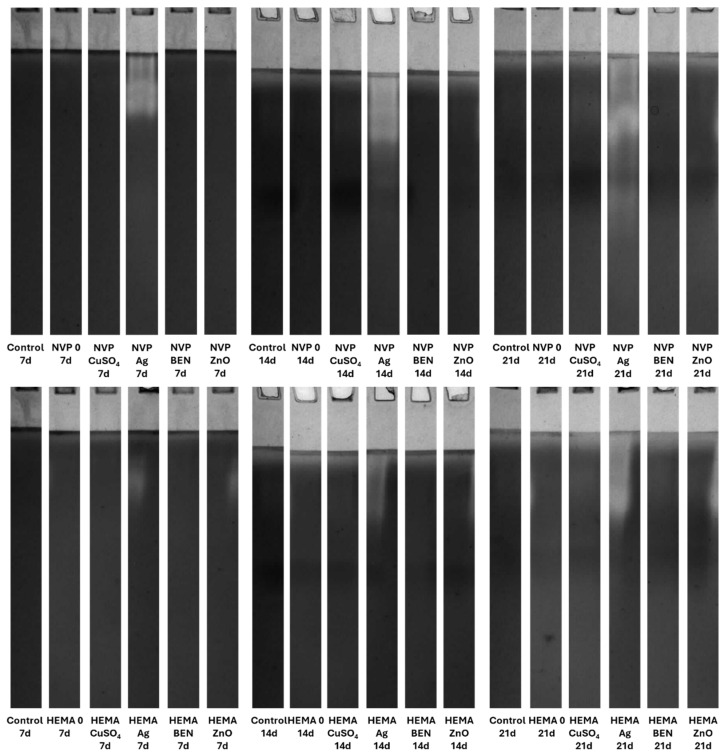
Electrophoretic detection of alkaline protease activity in the culture fluid of *C. unicolor* after 7, 14 and 21 days of cultivation in the presence of NVP- and HEMA-containing composite material fragments, following ethanol disinfection.

**Figure 17:**
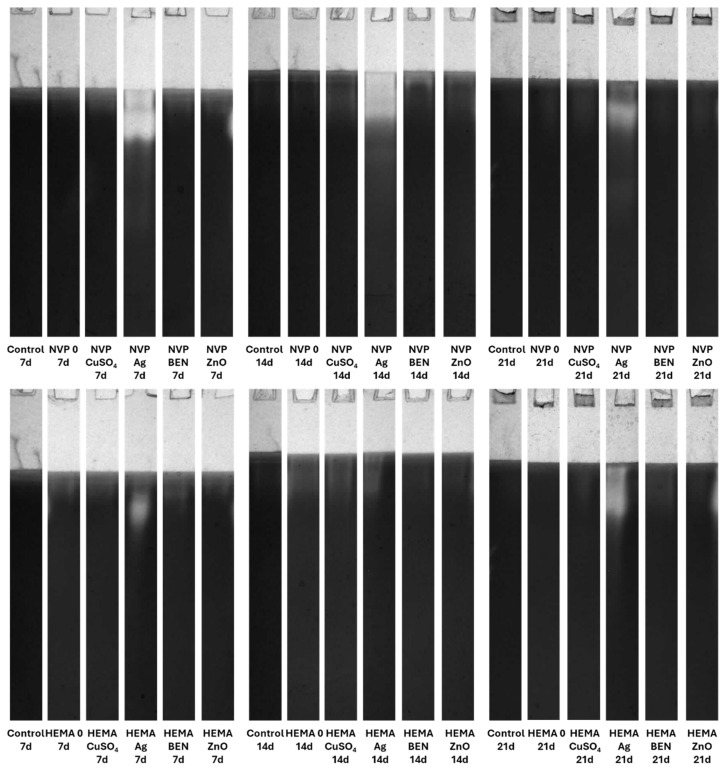
Zymographic detection of acid protease activity in the culture fluid of *C. unicolor* after 7, 14 and 21 days of cultivation in the presence of NVP- and HEMA-containing composite material fragments, following ethanol disinfection.

Both the acidic and alkaline protease activity showed a significant increase on each subsequent measurement day, for both NVP and HEMA in the preparations containing silver ions. This phenomenon is clearly observed in preparations after ethanol disinfection (Figure 16 and Figure 17).

**Figure 18:**
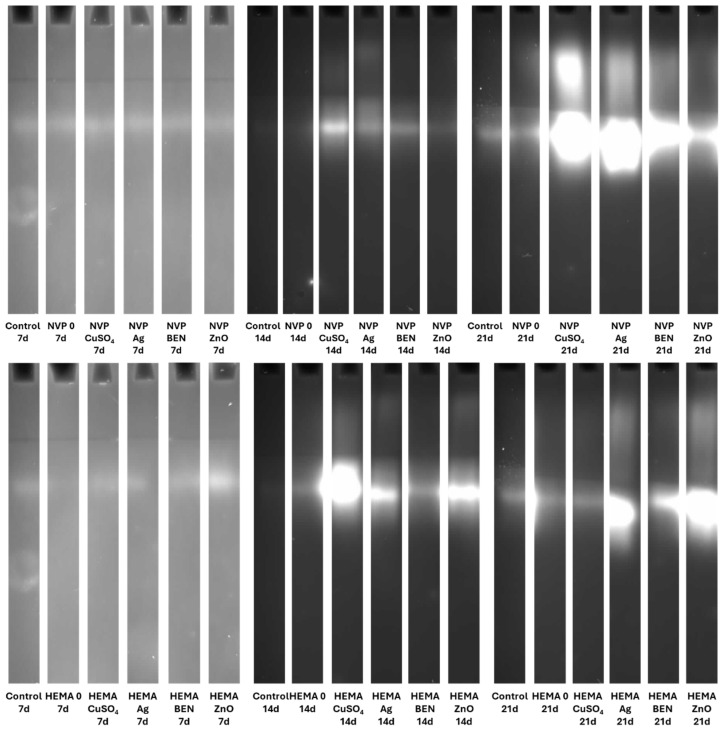
Electrophoretic detection of β-glucosidase activity in the culture fluid of *C. unicolor* after 7, 14 and 21 days of cultivation in the presence of NVP- and HEMA-containing composite material fragments, following ethanol disinfection.

There was an upward trend in β-glucosidase activity in preparations containing metal ions. An exceptionally high level of activity was observed on day 21 in NVP preparations containing silver and copper ions, compared to the control (Figure 18). High extracellular β-glucosidase activity, indicating increased polysaccharide metabolism, was also observed in both UV-sterilized NVP- and HEMA-containing polymers on day 21 of culture, with a certain level of variability in the case of the control and the modified polymers. The NVP- and CuSO_4_-modified polymer demonstrates high enzymatic activity as early as the 14th day of *C. unicolor* cultivation (Figure S16).

## 4. Discussion

The widespread use of antibiotics has led to many bacterial and fungal strains developing resistance to their effects [43,44,45,46]. One interesting solution is the synthesis of new composite materials with antimicrobial properties. Coating everyday objects, such as countertops, cabinets, handles, and handrails with these materials can reduce the spread and transmission of pathogenic microorganisms [47]. However, it is important to note that the production of plastics is steadily increasing. The world is grappling with the growing problem of polymer waste disposal [48]. From this point of view, it is crucial to search for new solutions in two areas: the synthesis of effective polymers and their degradation. Therefore, the research plan carried out in the present study addresses these needs adequately.

Firstly, new methacrylate-based polymers containing antimicrobial properties are likely to be biodegraded by wood-decaying fungi, such as the *C. unicolor* strain used in this study. Furthermore, bisphenol A, a component of epoxides, is degraded by laccase produced by fungi such as *Cerrena unicolor* and *Trametes hirsuta* [11,49,50]. Conversely, white-rot fungi can adapt their metabolic system to changes caused by the presence of a modified composite. Profilometric analysis revealed that the new composite materials can probably be degraded upon contact with the *C. unicolor* fungus. This is confirmed by the porosity coefficient (Ra) value determined for the composite samples. Additionally, changes in the surface of the composite materials were clearly visible in SEM images. The microbial degradation of polymeric materials containing either polyurethane or polyamide has been reported previously [51]. The impact of white wood rot fungi on composite materials incorporating biofillers is also well documented [6,52,53].

The use of a 70% ethanol solution to disinfect the composites before testing them with the *C. unicolor* fungus increased laccase activity relative to the control sample. This suggests that ethanol may facilitate the fungus’s access to components of the composites that it can use as inducers. These results also indicate that the fungus has the potential to degrade this type of composite material effectively. Similar results were obtained when polymers were modified with biofillers, such as corn cobs, wheat bran, and oat bran, or lignin [54]. In the majority of cases, UV-sterilized composites exhibited slightly lower laccase activity compared to control samples. This could be explained by the fact that the access to the active ingredients of the composites was more difficult, compared to composites sterilized with ethanol [55,56]. A similar relationship involving an increase in laccase activity after the addition of substances capable of inducing stress was observed with the addition of copper, silver, lead, and zinc [56]. To date, four distinct isoenzymes of laccase have been identified in *C. unicolor*, which were purified from cultures grown on LH medium supplemented with copper ions [57]. In contrast, the laccase zymograms obtained (Figure 15 and Figure S13) for polymers containing NVP and HEMA, with the addition of copper ions and nanosilver, following ethanol sterilization, clearly indicate the presence of additional bands corresponding to slower-migrating laccase isoforms. This finding signifies not solely quantitative but also qualitative alterations in enzyme activity, most plausibly attributable to the culture conditions applied to *C. unicolor*, which are recognized to exert a pronounced influence on the expression levels of laccase genes and their post-translational processing [58,59,60]. The results obtained indicate new perspectives for using the tested composites, especially those previously treated with ethanol to enhance the biodegradation potential in the tested group of fungi. It is probable that the introduction of polymers prepared in this way could increase the degradation effectiveness in other biodegradation-resistant materials and environmental pollutants. Analysis of the present results revealed a clear influence of the new polymers on the modification of the metabolic profile of *C. unicolor*. Upon determining the concentration of extracellular proteins, it became clear that their content depended on the type of the monomer used. Changes in the protein concentration are most often a consequence of xenobiotic compounds or stress factors. Previous studies have observed an increase in protein content in the culture filtrate caused by either light or oxidative stress [61,62].

The increase in the relative superoxide anion radical content, compared to the control samples, indicates that the analyzed composite materials stimulate biochemical reactions in fungal cells. Free radicals are likely to be by-products of these reactions. However, an excess of free radicals is detrimental to cell viability. The presence of stress factors, such as composite materials, induces oxidative stress. Similar conclusions were reached by [55,63,64]. On the other hand, this type of reaction may facilitate the decomposition of polymers like those presented in this work and increase their biodegradation rate.

The phenolic compounds released into the environment by wood-rotting fungi such as *C. unicolor*, which were studied in this work, have certain therapeutic or antioxidant properties [65]. The addition of the composite materials to the culture medium decreased the level of extracellular phenolic compounds. Conversely, the total sugar content increased. Analyzing the information presented above, it can be concluded that there is some interaction between the composite materials and the mycelium of *C. unicolor*. This interaction seems promising as a method of modulating the activity of the fungus. However, polymers from this group can be biodegraded by this organism [2,66].

Copper ions are known as inducers of laccase in *C. unicolor* [8]. As reported by Galhaup et al. [67], silver ions also appeared to stimulate laccase biosynthesis in *Trametes pubescens* to a slight extent. Significant changes in the activity of acidic and alkaline proteases were observed in the *C. unicolor* cultures that had been incubated with HEMA- and NVP-based composites, which had been sterilized with ethanol and modified with nanosilver. This may suggest both increased protein turnover and, more likely, increased cell lysis associated with chemical stress. However, the induction mechanism is likely much more complex. These cases support the hypothesis that ethanol increases the bioavailability of metal ions to a greater extent than UV sterilization.

## 5. Conclusions

In conclusion, this study demonstrates that novel methacrylate-based composites containing antimicrobial agents, when introduced into cultures of the white-rot fungus *C. unicolor*, can effectively stimulate the activity of biotechnologically relevant enzymes such as laccase. The presence of stress-inducing composites also significantly affects other parameters, including the levels of free radicals, phenolic compounds, and proteins, as well as protease and β-glucosidase activities. Notably, pronounced alterations in the porosity of the composites, confirmed by profilometric measurements and SEM analysis, indicate that *C. unicolor* may hold considerable potential for applications in polymer recycling and biodegradation of environmental pollutants. Furthermore, the proposed approach offers a promising strategy to enhance the biodegradation capacity of this fungus, for instance by targeted stimulation of laccase activity—an aspect of particular relevance to modern biotechnological processes.

## Figures and Tables

**Table 1:** Zinc concentration after release from NVP- or HEMA-containing composites during incubation (10th and 20th days) in Lindeberg–Holm (LH) medium, or water. Values marked with different superscript letters are statistically significant (*p* < 0.05—Dunnett’s post hoc test).

Composite/Days	10 Days [mg/L]	20 Days [mg/L]
NVP+ZnO LH	2.62 ± 0.01 ^a^	3.73 ± 0.02 ^a^
HEMA+ZnO LH	2.62 ± 0.01 ^a^	5.20 ± 0.03 ^ab^
NVP+ZnO water	2.37 ± 0.00 ^b^	5.75 ± 0.03 ^bc^
HEMA+ZnO water	3.12 ± 0.01 ^c^	7.67 ± 0.01 ^c^

**Table 2:** Copper concentration after release from NVP- or HEMA-containing composites during incubation (10th and 20th days) in Lindeberg–Holm (LH) medium, or water. Values marked with different superscript letters are statistically significant (*p* < 0.05—Dunnett’s post hoc test).

Composite/Days	10 Days [mg/L]	20 Days [mg/L]
NVP+CuSO_4_ LH	21.13 ± 0.07 ^a^	31.09 ± 0.40 ^a^
HEMA+CuSO_4_ LH	128.54 ± 0.06 ^b^	354.98 ± 3.75 ^b^
NVP+CuSO_4_ water	117.22 ± 2.64 ^c^	319.07 ± 1.12 ^c^
HEMA+CuSO_4_ water	22.49 ± 0.02 ^d^	23.80 ± 0.64 ^d^

**Table 3:** Silver concentration after release from NVP- or HEMA-containing composites during incubation (10th and 20th days) in Lindeberg–Holm (LH) medium, or water. Values marked with different superscript letters are statistically significant (*p* < 0.05—Dunnett’s post hoc test).

Composite/Days	10 Days [µg/L]	20 Days [µg/L]
NVP+Ag LH	56.72 ± 1.23 ^a^	128.22 ± 1.12 ^a^
HEMA+Ag LH	27.54 ± 2.24 ^b^	32.22 ± 0.75 ^b^
NVP+Ag water	150.20 ± 4.65 ^c^	413.88 ± 2.45 ^c^
HEMA+Ag water	428.43 ± 3.26 ^d^	1671.30 ± 5.12 ^d^

**Table 4:** Benzethonium chloride concentration (BEN) after release from NVP- or HEMA-containing composites during incubation (10th and 20th days) in Lindeberg–Holm (LH) medium, or water. Values marked with different superscript letters are statistically significant (*p* < 0.05—Dunnett’s post hoc test).

Composite/Days	10 Days [ppm]	20 Days [ppm]
NVP+BEN LH	2.61 ± 0.01 ^a^	3.31 ± 0.03 ^a^
HEMA+BEN LH	0.23 ± 0.00 ^b^	0.27 ± 0.00 ^b^
NVP+BEN water	22.00 ± 0.03 ^c^	23.13 ± 0.02 ^c^
HEMA+BEN water	3.18 ± 0.00 ^d^	3.56 ± 0.00 ^d^

**Table 5:** Bisphenol A glycerolate dimethacrylate concentration after release from NVP- or HEMA-containing composites during incubation (10th and 20th days) in Lindeberg–Holm (LH) medium, or water. Values marked with different superscript letters are statistically significant (*p* < 0.05—Dunnett’s post hoc test).

Composite/Days	10 Days [µg/L]	20 Days [µg/L]
NVP LH	0.00 ± 0.00 ^a^	0.00 ± 0.00 ^a^
HEMA LH	1.20 ± 0.01 ^b^	1.40 ± 0.01 ^b^
NVP water	0.00 ± 0.00 ^a^	0.00 ± 0.00 ^a^
HEMA water	0.30 ± 0.02 ^c^	0.30 ± 0.01 ^c^

**Table 6:** Changes in the value of Ra obtained in profilometric analysis of NVP- and HEMA-containing composites that were subjected to a release experiment for 20 days and 21 days biodegradation by *C. unicolor*. The evaluation was carried out for the control material and the material exposed to UV sterilization and the 70% ethanol solution.

Polymer Sample	Ra Value [nm]	Polymer Sample	Ra Value [nm]
NVP control	0.92 ± 0.05	HEMA control	1.84 ± 0.00
NVP 21d/ethanol	63.58 ± 1.30	HEMA 21d/ethanol	2.99 ± 0.04
NVP 21d/UV	1.43 ± 0.03	HEMA 21d/UV	4.54 ± 0.03
NVP+BEN control	0.54 ± 0.01	HEMA+BEN control	0.92 ± 0.00
NVP+BEN 21d/ethanol	2.68 ± 0.03	HEMA+BEN 21d/ethanol	1.23 ± 0.01
NVP+BEN 21d/UV	348.54 ± 3.3	HEMA+BEN 21d/UV	719.50 ± 3.4

## Data Availability

The raw data supporting the conclusions of this article will be made available by the authors on request.

## Supplementary Materials

The following supporting information can be downloaded at: https://www.mdpi.com/article/10.3390/biom16050731/s1. Figure S1. Evaluation of structural alterations in HEMA-containing composites based on SEM analysis. Non-sterilized materials that were not in contact with the culture medium, water, or microorganisms were used as a control (HEMA control). Samples after the release experiment (HEMA (water), HEMA (LH), and in those sterilized with UV or ethanol and subjected to 21-day biodegradation by *C. unicolor* (HEMA (21d/ethanol), HEMA (21d/UV)) were also analyzed; Figure S2. Evaluation of structural alterations in NVP-containing composites based on SEM analysis. Non-sterilized materials that were not in contact with the culture medium, water, or microorganisms were used as a control (NVP control). Samples after the release experiment (NVP (water), NVP (LH), and in those sterilized with UV or ethanol and subjected to 21-day biodegradation by *C. unicolor* (NVP (21d/ethanol), NVP (21d/UV)) were also analyzed; Figure S3. Analysis of extracellular laccase activity after cultivation of the fungus *C. unicolor* for 7, 14 or 21 days in the presence of HEMA-containing composite material fragments, following UV sterilization. (A)—samples with nanosilver; (B)—samples with benzethonium chloride; (C)—samples with zinc oxide; (D)—samples with copper(II) sulfate; bars represent means with error bars denoting standard deviation (SD) from three measurements (*n* = 3). Significance against control determined by Dunnett’s test, where *p* value is: *** 0.001; Figure S4. Analysis of extracellular laccase activity after cultivation of the fungus *C. unicolor* for 7, 14 or 21 days in the presence of NVP-containing composite material fragments, following UV disinfection. (A)—samples with nanosilver; (B)—samples with benzethonium chloride; (C)—samples with zinc oxide; (D)—samples with copper(II) sulfate; bars represent means with error bars denoting standard deviation (SD) from three measurements (*n* = 3). Significance against control determined by Dunnett’s test, where *p* value is: *** 0.001; * 0.05; Figure S5. Concentration of proteins in the culture fluid of *C. unicolor* after 7, 14 and 21 days of cultivation in the presence of HEMA-containing composite material fragments, following UV sterilization. (A)—samples with nanosilver; (B)—samples with benzethonium chloride; (C)—samples with zinc oxide; (D)—samples with copper(II) sulfate; bars represent means with error bars denoting standard deviation (SD) from three measurements (*n* = 3). Significance against control determined by Dunnett’s test, where *p* value is: *** 0.001; Figure S6. Concentration of proteins in the culture fluid of *C. unicolor* after 7, 14 and 21 days of cultivation in the presence of NVP-containing composite material fragments, following UV sterilization. (A)—samples with nanosilver; (B)—samples with benzethonium chloride; (C)—samples with zinc oxide; (D)—samples with copper(II) sulfate; bars represent means with error bars denoting standard deviation (SD) from three measurements (*n* = 3). Significance against control determined by Dunnett’s test, where *p* value is: *** 0.001; ** 0.01; * 0.05; Figure S7. Relative level of superoxide anion radicals—analysis conducted for HEMA-containing composites after UV disinfection. (A)—samples with nanosilver; (B)—samples with benzethonium chloride; (C)—samples with zinc oxide; (D)—samples with copper(II) sulfate; bars represent means with error bars denoting standard deviation (SD) from three measurements (*n* = 3). Significance against control determined by Dunnett’s test, where *p* value is: *** 0.001; * 0.05; Figure S8. Relative level of superoxide anion radicals—analysis conducted for NVP-containing composites after UV disinfection. (A)—samples with nanosilver; (B)—samples with benzethonium chloride; (C)—samples with zinc oxide; (D)—samples with copper(II) sulfate; bars represent means with error bars denoting standard deviation (SD) from three measurements (*n* = 3). Significance against control determined by Dunnett’s test, where *p* value is: *** 0.001; * 0.05; Figure S9. Phenolic compounds content in the culture fluid of *C. unicolor* after 7, 14 and 21 days of cultivation in the presence of HEMA-containing composite material fragments, following UV sterilization. (A)—samples with nanosilver; (B)—samples with benzethonium chloride; (C)—samples with zinc oxide; (D)—samples with copper(II) sulfate; bars represent means with error bars denoting standard deviation (SD) from three measurements (*n* = 3). Significance against control determined by Dunnett’s test, where *p* value is: *** 0.001; ** 0.01; * 0.05; Figure S10. Phenolic compounds content in the culture fluid of *C. unicolor* after 7, 14 and 21 days of cultivation in the presence of NVP-containing composite material fragments, following UV sterilization. (A)—samples with nanosilver; (B)—samples with benzethonium chloride; (C)—samples with zinc oxide; (D)—samples with copper(II) sulfate; bars represent means with error bars denoting standard deviation (SD) from three measurements (*n* = 3). Significance against control determined by Dunnett’s test, where *p* value is: *** 0.001; Figure S11. Extracellular total carbohydrates content in the culture fluid of *C. unicolor* after 7, 14 and 21 days of cultivation in the presence of HEMA-containing composite material fragments, following UV sterilization. (A)—samples with nanosilver; (B)—samples with benzethonium chloride; (C)—samples with zinc oxide; (D)—samples with copper(II) sulfate; bars represent means with error bars denoting standard deviation (SD) from three measurements (*n* = 3) Significance against control determined by Dunnett’s test, where *p* value is: *** 0.001; * 0.05; Figure S12. Extracellular total carbohydrates content in the culture fluid of *C. unicolor* after 7, 14 and 21 days of cultivation in the presence of NVP-containing composite material fragments, following UV sterilization. (A)—samples with nanosilver; (B)—samples with benzethonium chloride; (C)—samples with zinc oxide; (D)—samples with copper(II) sulfate; bars represent means with error bars denoting standard deviation (SD) from three measurements (*n* = 3) Significance against control determined by Dunnett’s test, where *p* value is: *** 0.001; ** 0.01; Figure S13. Zymographic detection of extracellular laccase activity in the culture fluid of *C. unicolor* after 7, 14 and 21 days of cultivation in the presence of NVP- and HEMA-containing composite material fragments, following UV sterilization; Figure S14. Electrophoretic detection of alkaline protease activity in the culture fluid of *C. unicolor* after 7, 14 and 21 days of cultivation in the presence of NVP- and HEMA-containing composite material fragments, following UV sterilization; Figure S15. Zymographic detection of acid protease activity in the culture fluid of *C. unicolor* after 7, 14 and 21 days of cultivation in the presence of NVP- and HEMA-containing composite material fragments, following UV sterilization; Figure S16. Electrophoretic detection of β-glucosidase activity in the culture fluid of *C. unicolor* after 7, 14 and 21 days of cultivation in the presence of NVP- and HEMA-containing composite material fragments, following UV sterilization. File S1. Original electrophoresis gel.

## Abbreviations

The following abbreviations are used in this manuscript:

BPA.DM	Bisphenol A glycerolate dimethacrylate
NVP	1-vinyl-2-pyrrolidone
HEMA	2-hydroxyethyl methacrylate
ZnO	Zinc oxide
CuSO_4_	Copper sulfate
Ag	Nanosilver
IQ	2,2-dimethoxy-2-phenylacetophenone
BEN	Benzethonium chloride
LH	Lindeberg–Holm liquid medium
SEM	Scanning electron microscopy
NVP+ZnO LH	Composite material containing 1-vinyl-2-pyrrolidone and zinc oxide after incubation in Lindeberg–Holm liquid medium
HEMA+ZnO LH	Composite material containing 2-hydroxyethyl methacrylate and zinc oxide after incubation in Lindeberg–Holm liquid medium
NVP+ZnO water	Composite material containing 1-vinyl-2-pyrrolidone and zinc oxide after incubation in water
HEMA+ZnO water	Composite material containing 2-hydroxyethyl methacrylate and zinc oxide after incubation in water
NVP+CuSO4 LH	Composite material containing 1-vinyl-2-pyrrolidone and copper sulfate after incubation in Lindeberg–Holm liquid medium
HEMA+CuSO4 LH	Composite material containing 2-hydroxyethyl methacrylate and copper sulfate after incubation in Lindeberg–Holm liquid medium
NVP+CuSO4 water	Composite material containing 1-vinyl-2-pyrrolidone and copper sulfate after incubation in water
HEMA+CuSO4 water	Composite material containing 2-hydroxyethyl methacrylate and copper sulfate after incubation in water
NVP+Ag LH	Composite material containing 1-vinyl-2-pyrrolidone and nanosilver after incubation in Lindeberg–Holm liquid medium
HEMA+Ag LH	Composite material containing 2-hydroxyethyl methacrylate and nanosilver after incubation in Lindeberg–Holm liquid medium
NVP+Ag water	Composite material containing 1-vinyl-2-pyrrolidone and nanosilver after incubation in water
HEMA+Ag water	Composite material containing 2-hydroxyethyl methacrylate and nanosilver after incubation in water
NVP+BEN LH	Composite material containing 1-vinyl-2-pyrrolidone and benzethonium chloride after incubation in Lindeberg–Holm liquid medium
HEMA+BEN LH	Composite material containing 2-hydroxyethyl methacrylate and benzethonium chloride after incubation in Lindeberg–Holm liquid medium
NVP+BEN water	Composite material containing 1-vinyl-2-pyrrolidone after incubation in water
HEMA+BEN water	Composite material containing 2-hydroxyethyl methacrylate and benzethonium chloride after incubation in water
NVP LH	Composite material containing 1-vinyl-2-pyrrolidone after incubation in Lindeberg–Holm liquid medium
HEMA LH	Composite material containing 2-hydroxyethyl after incubation in Lindeberg–Holm liquid medium
NVP water	Composite material containing 1-vinyl-2-pyrrolidone after incubation in water
HEMA water	Composite material containing 2-hydroxyethyl methacrylate after incubation in water
