# Supplementary material for: New Cross-Linked Polymeric Materials Modified with Antimicrobial Compounds in Relation to Their Biological Activities and Biodegradation by the Laccase-Producing Fungus Cerrena unicolor
Source: Biomolecules. 2026 May 15;16(5):731. doi: 10.3390/biom16050731 (PMC13204337; doi:10.3390/biom16050731)
Supplement: Supplementary file 1 [file biomolecules-16-00731-s001.zip › biomolecules-4261694-supplementary.pdf]

*biomolecules*

*Article*

# New cross-linked polymeric materials modified with antimicrobial compounds in relation to their biological activities and biodegradation by the laccase-producing fungus *Cerrena unicolor*

**Karolina Kiełczewska-Klim[1*], Dawid Stefaniuk[2], Marcin Grąz[2], Rafał Typek[3], Bożena Pawlikowska-Pawlęga[4], Anna Pawlik[2], Beata Podkościelna[1] and Magdalena Jaszek[2*]**

[1] Department of Polymer Chemistry, Maria Curie-Sklodowska University in Lublin, Gliniana 33, 20-614 Lublin, Poland; karolina.mlynarczyk@mail.umcs.pl (K.K-K.); beata.podkoscielna@mail.umcs.pl (B.P.)
[2] Department of Biochemistry and Biotechnology, Institute of Biological Sciences, Maria Curie-Sklodowska University in Lublin, Akademicka 19, 20-033 Lublin, Poland; dawid.stefaniuk@mail.umcs.pl (D.S.); marcin.graz@mail.umcs.pl (M.G.); anna.pawlik@mail.umcs.pl (A.P.); magdalena.jaszek@mail.umcs.pl (M.J.)
[3] Department of Chromatography, Institute of Chemical Sciences, Faculty of Chemistry, Maria Curie Sklodowska University in Lublin, Maria Curie-Sklodowska Square 3, 20-031 Lublin, Poland; rafal.typek@mail.umcs.pl (R.T.)
[4] Department of Functional Anatomy and Cytobiology, Institute of Biological Sciences, Maria Curie-Sklodowska University, Akademicka 19, 20-033 Lublin, Poland; bozena.pawlikowska-pawlega@mail.umcs.pl (B.P-P.)
* Correspondence: magdalena.jaszek@mail.umcs.pl; +48815375017 (M.J.); karolina.mlynarczyk@mail.umcs.pl; +48815242251 (K.K-K.)

**Keywords:** cross-linked composites, zinc oxide, copper(II) sulfate, benzethonium chloride, nanosilver, fungi, laccase, biodegradation, SEM, profilometric analysis

Academic Editor: Firstname Lastname

Received: date
Revised: date
Accepted: date
Published: date

**Copyright:** © 2026 by the authors. Submitted for possible open access publication under the terms and conditions of the Creative Commons Attribution (CC BY) license.

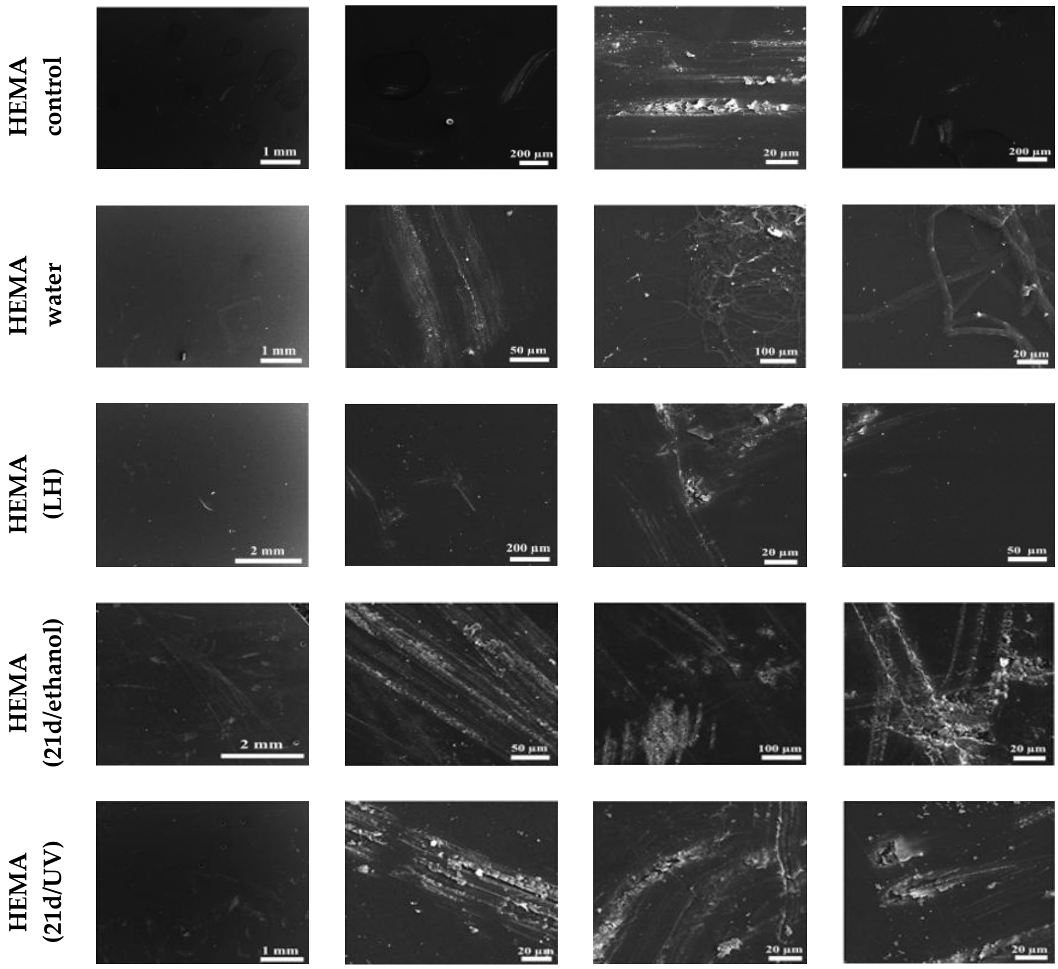


**Figure S1.** Evaluation of structural alterations in HEMA-containing composites based on SEM analysis. Non-sterilized materials that were not in contact with the culture medium, water, or microorganisms were used as a control (HEMA control). Samples after the release experiment (HEMA (water), HEMA (LH), as well as in those sterilized with UV or ethanol and subjected to 21-day biodegradation by *C. unicolor* (HEMA (21d/ethanol), HEMA (21d/UV)) were also analyzed.

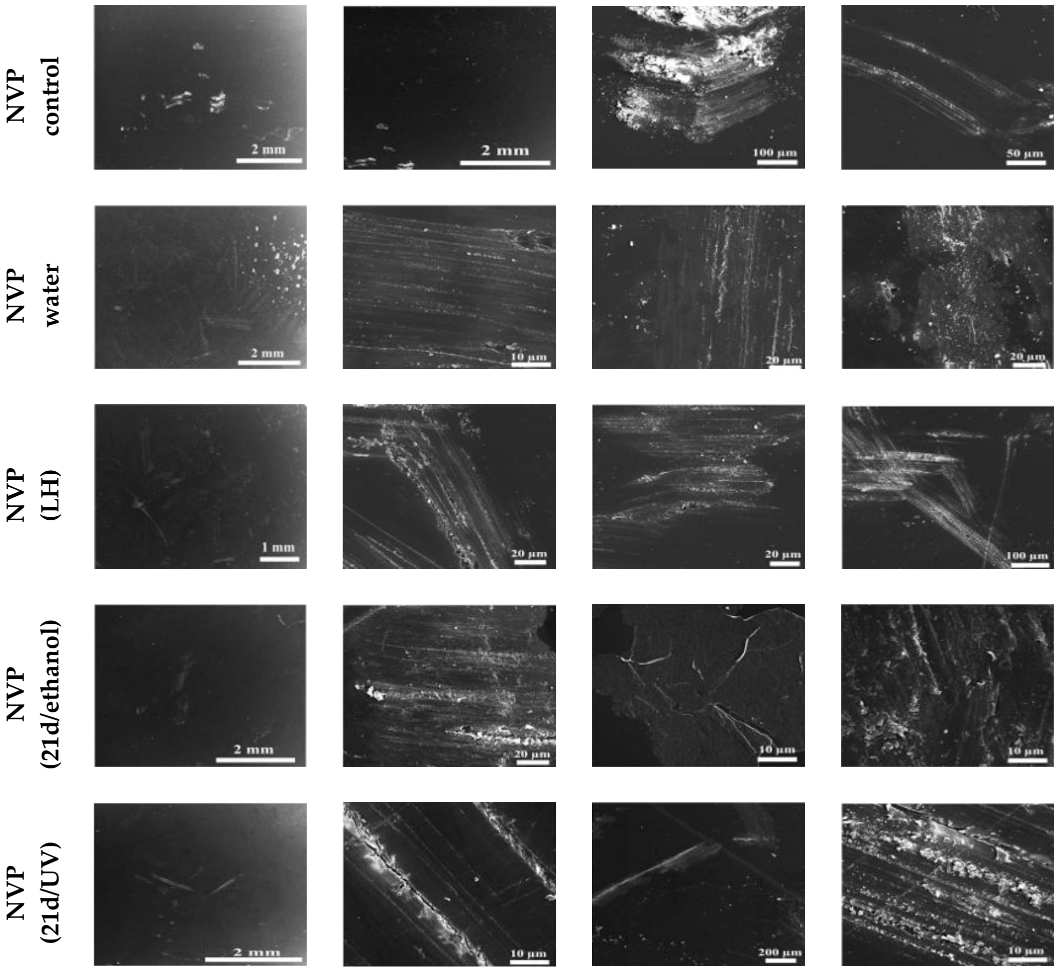


**Figure S2.** Evaluation of structural alterations in NVP-containing composites based on SEM analysis. Non-sterilized materials that were not in contact with the culture medium, water, or microorganisms were used as a control (NVP control). Samples after the release experiment (NVP (water), NVP (LH), as well as in those sterilized with UV or ethanol and subjected to 21-day biodegradation by *C. unicolor* (NVP (21d/ethanol), NVP (21d/UV)) were also analyzed.

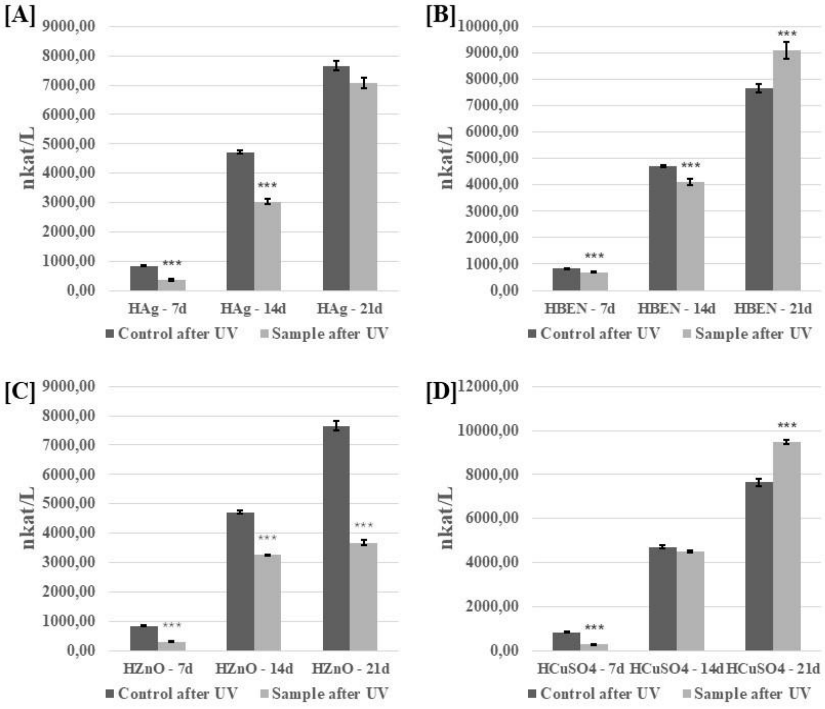


**Figure S3.** Analysis of extracellular laccase activity after cultivation of the fungus *C. unicolor* for 7, 14 or 21 days in the presence of HEMA-containing composite material fragments, following UV sterilization. [A] – samples with nanosilver; [B] – samples with benzethonium chloride; [C] – samples with zinc oxide; [D] – samples with copper(II) sulfate; bars represent means with error bars denoting standard deviation (SD) from three measurements (n = 3). Significance against control determined by Dunnett's test, where p value is: *** 0.001; ** 0.01; * 0.05.

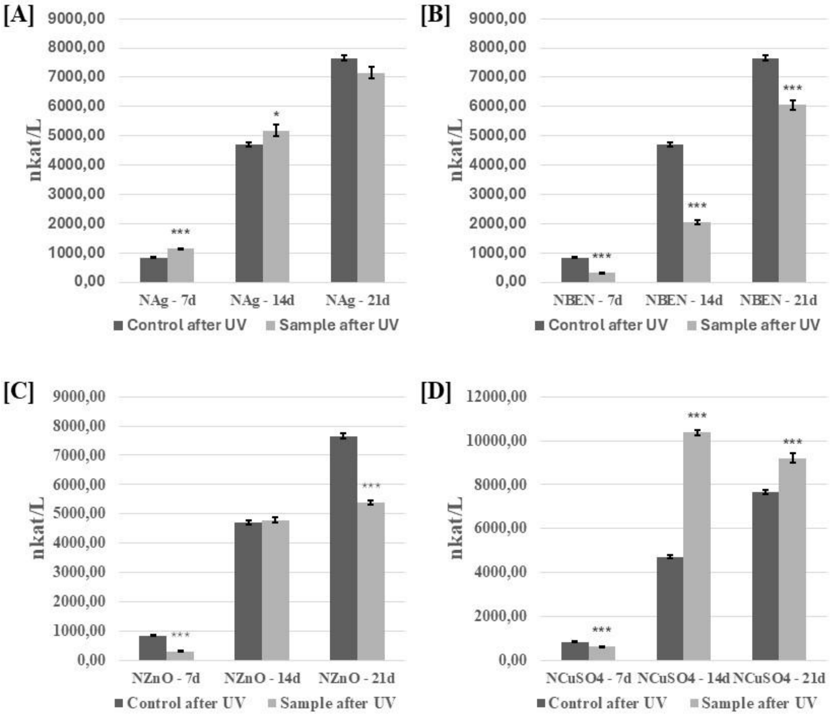

**Figure S4.** Analysis of extracellular laccase activity after cultivation of the fungus C. unicolor for 7, 14 or 21 days in the presence of NVP-containing composite material fragments, following UV disinfection. [A] – samples with nanosilver; [B] – samples with benzethonium chloride; [C] – samples with zinc oxide; [D] – samples with copper(II) sulfate; bars represent means with error bars denoting standard deviation (SD) from three measurements (n = 3). Significance against control determined by Dunnett's test, where p value is: *** 0.001; ** 0.01; * 0.05.

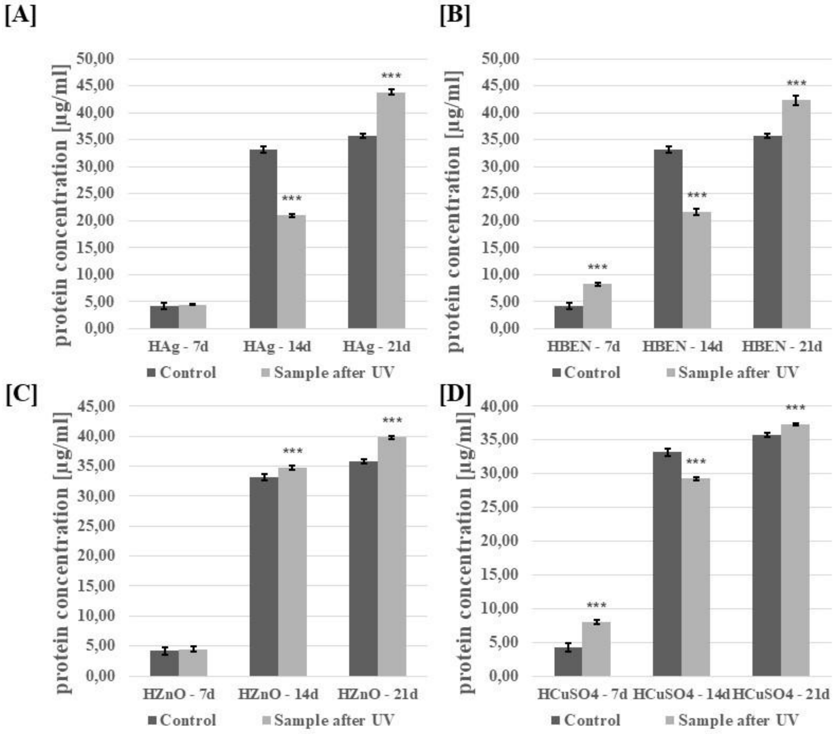


**Figure S5.** Concentration of proteins in the culture fluid of *C. unicolor* after 7, 14 and 21 days of cultivation in the presence of HEMA-containing composite material fragments, following UV sterilization. [A] – samples with nanosilver; [B] – samples with benzethonium chloride; [C] – samples with zinc oxide; [D] – samples with copper(II) sulfate; bars represent means with error bars denoting standard deviation (SD) from three measurements (n = 3). Significance against control determined by Dunnett's test, where p value is: *** 0.001; ** 0.01; * 0.05.

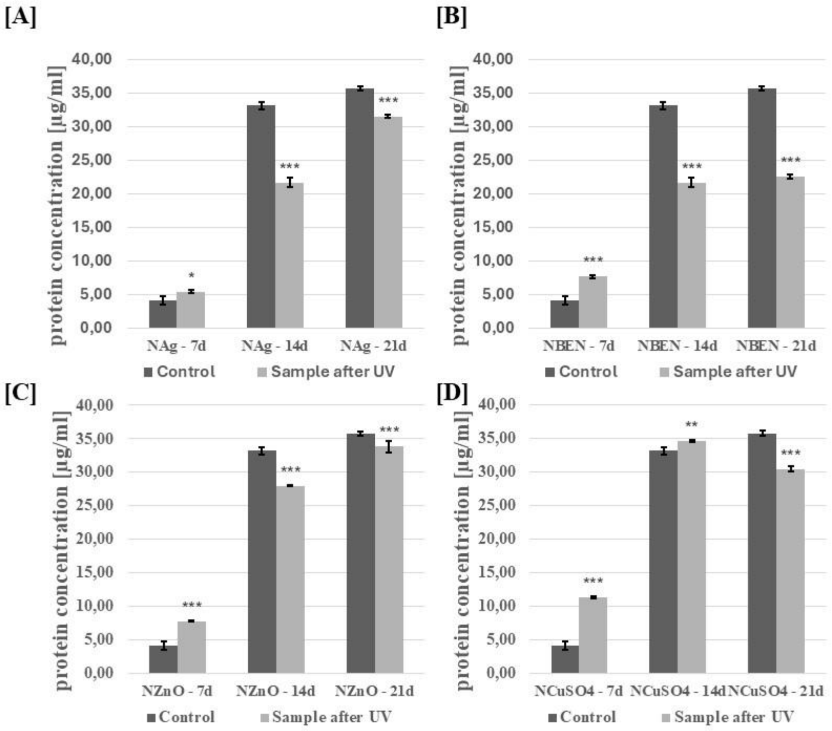


**Figure S6.** Concentration of proteins in the culture fluid of *C. unicolor* after 7, 14 and 21 days of cultivation in the presence of NVP-containing composite material fragments, following UV sterilization. [A] – samples with nanosilver; [B] – samples with benzethonium chloride; [C] – samples with zinc oxide; [D] – samples with copper(II) sulfate; bars represent means with error bars denoting standard deviation (SD) from three measurements (n = 3). Significance against control determined by Dunnett's test, where p value is: *** 0.001; ** 0.01; * 0.05.

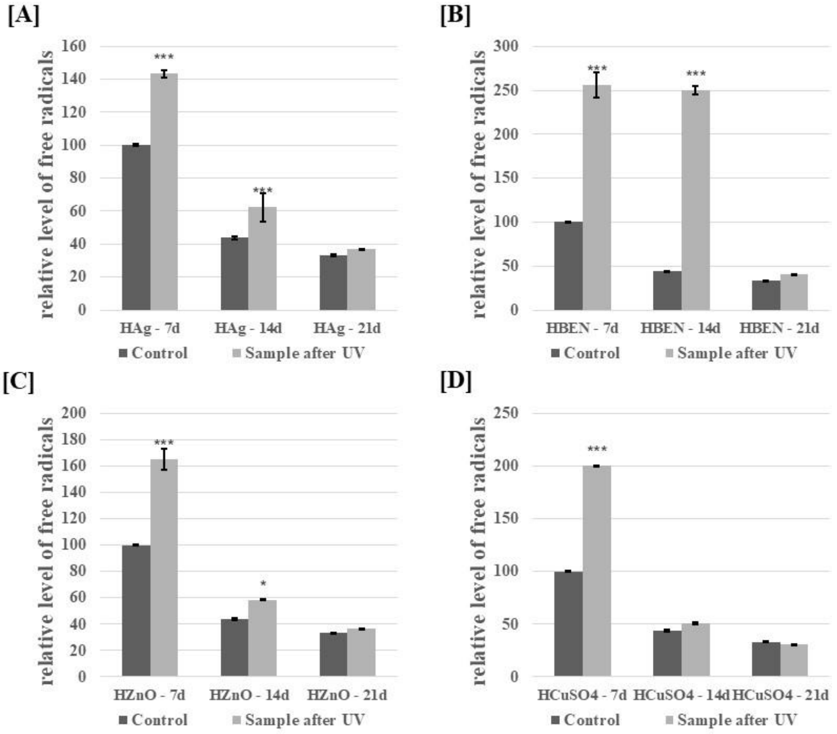

**Figure S7.** Relative level of superoxide anion radicals - analysis conducted for HEMA-containing composites after UV disinfection. [A] – samples with nanosilver; [B] – samples with benzethonium chloride; [C] – samples with zinc oxide; [D] – samples with copper(II) sulfate; bars represent means with error bars denoting standard deviation (SD) from three measurements (n = 3). Significance against control determined by Dunnett's test, where p value is: *** 0.001; ** 0.01; * 0.05.

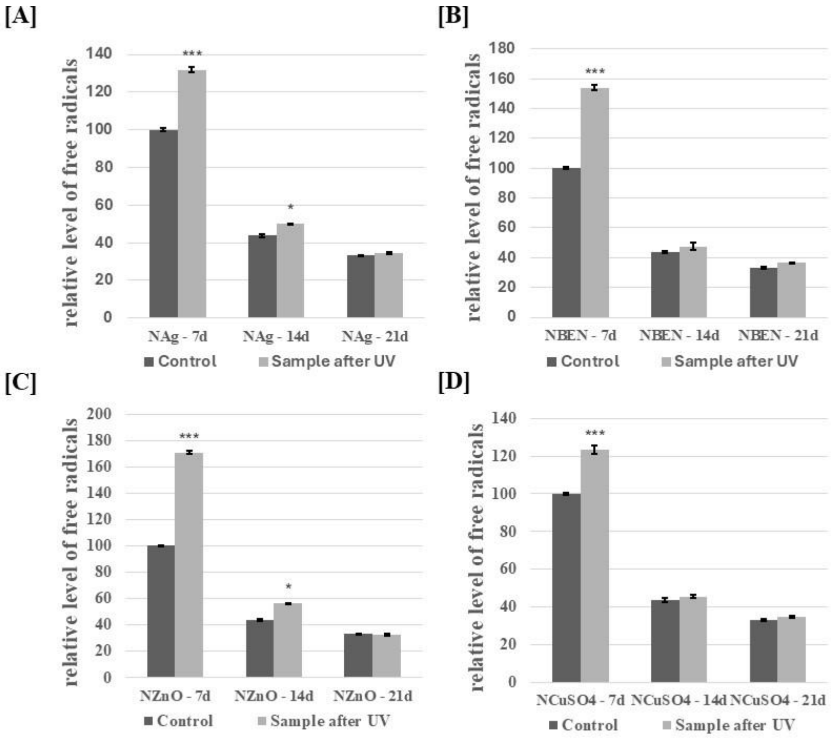


**Figure S8.** Relative level of superoxide anion radicals - analysis conducted for NVP-containing composites after UV disinfection. [A] – samples with nanosilver; [B] – samples with benzethonium chloride; [C] – samples with zinc oxide; [D] – samples with copper(II) sulfate; bars represent means with error bars denoting standard deviation (SD) from three measurements (n = 3). Significance against control determined by Dunnett's test, where p value is: *** 0.001; ** 0.01; * 0.05.

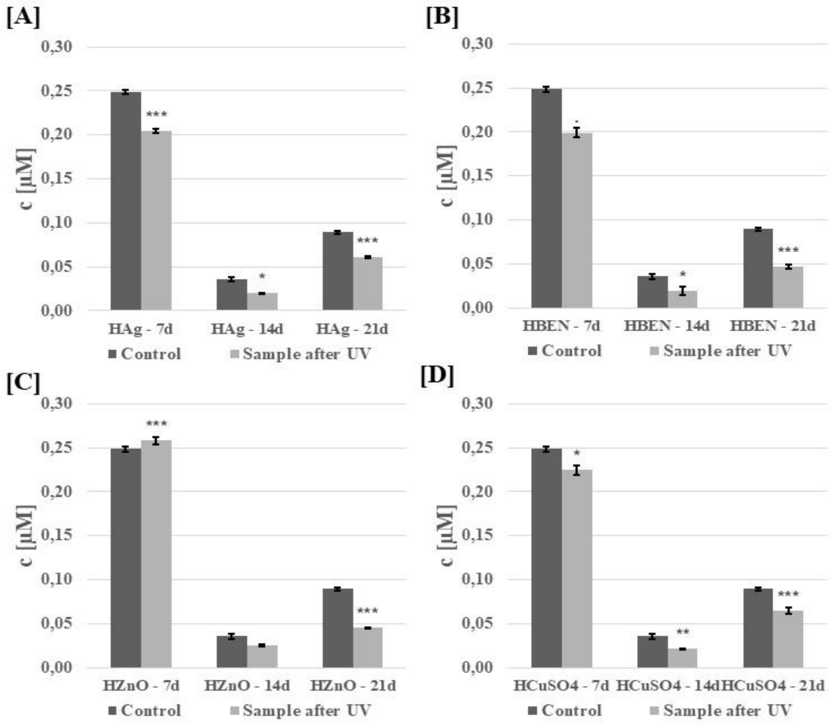


**Figure S9.** Phenolic compounds content in the culture fluid of *C. unicolor* after 7, 14 and 21 days of cultivation in the presence of HEMA-containing composite material fragments, following UV sterilization. [A] – samples with nanosilver; [B] – samples with benzethonium chloride; [C] – samples with zinc oxide; [D] – samples with copper(II) sulfate; bars represent means with error bars denoting standard deviation (SD) from three measurements (n = 3). Significance against control determined by Dunnett's test, where p value is: *** 0.001; ** 0.01; * 0.05.

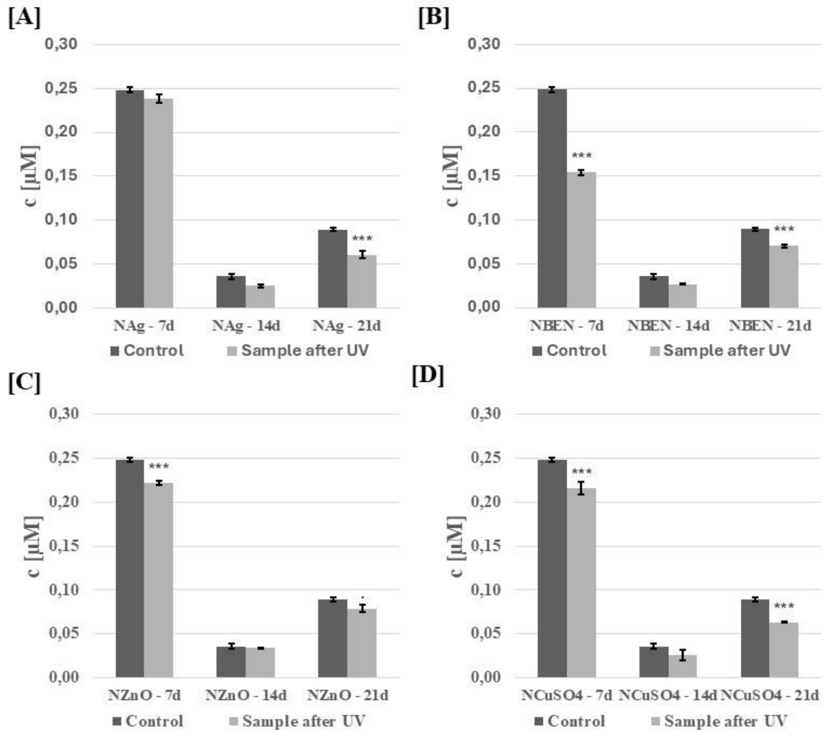

**Figure S10.** Phenolic compounds content in the culture fluid of *C. unicolor* after 7, 14 and 21 days of cultivation in the presence of NVP-containing composite material fragments, following UV sterilization. [A] – samples with nanosilver; [B] – samples with benzethonium chloride; [C] – samples with zinc oxide; [D] – samples with copper(II) sulfate; bars represent means with error bars denoting standard deviation (SD) from three measurements (n = 3). Significance against control determined by Dunnett's test, where p value is: *** 0.001; ** 0.01; * 0.05.

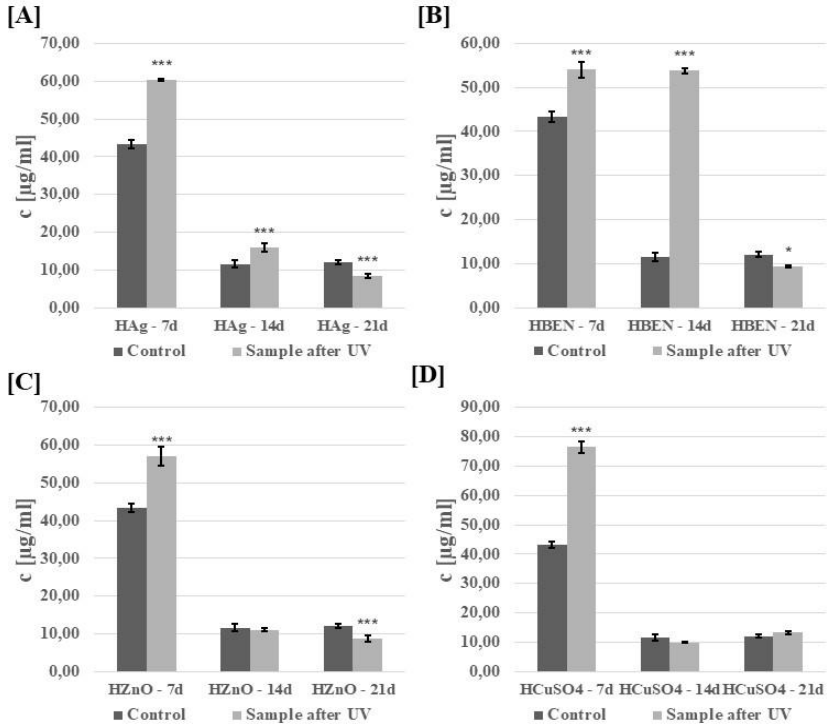


**Figure S11.** Extracellular total carbohydrates content in the culture fluid of *C. unicolor* after 7, 14 and 21 days of cultivation in the presence of HEMA-containing composite material fragments, following UV sterilization. [A] – samples with nanosilver; [B] – samples with benzethonium chloride; [C] – samples with zinc oxide; [D] – samples with copper(II) sulfate; bars represent means with error bars denoting standard deviation (SD) from three measurements (n = 3). Significance against control determined by Dunnett's test, where p value is: *** 0.001; ** 0.01; * 0.05.

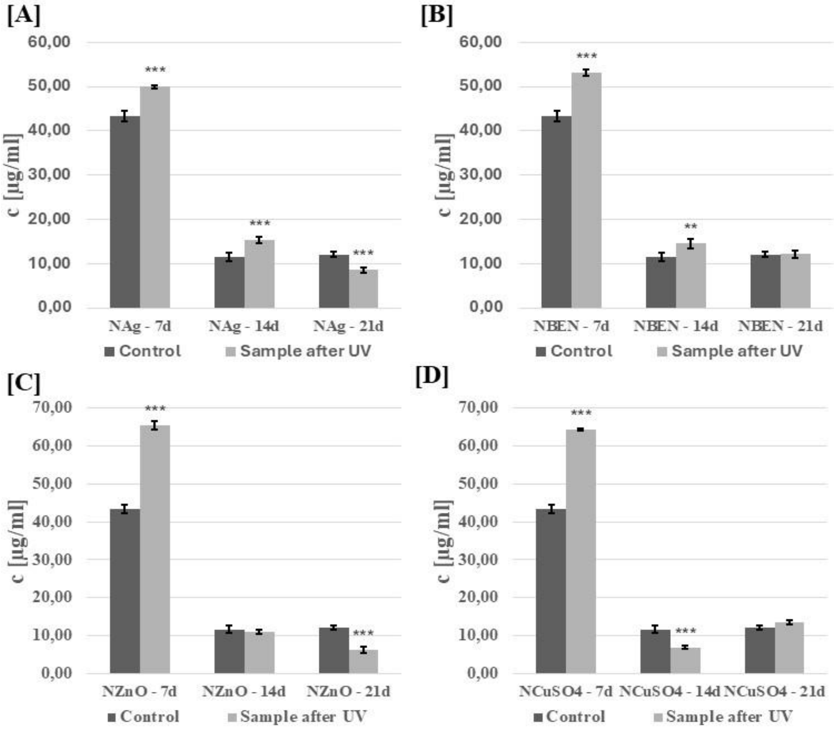


**Figure S12.** Extracellular total carbohydrates content in the culture fluid of *C. unicolor* after 7, 14 and 21 days of cultivation in the presence of NVP-containing composite material fragments, following UV sterilization. [A] – samples with nanosilver; [B] – samples with benzethonium chloride; [C] – samples with zinc oxide; [D] – samples with copper(II) sulfate; bars represent means with error bars denoting standard deviation (SD) from three measurements (n = 3). Significance against control determined by Dunnett's test, where p value is: *** 0.001; ** 0.01; * 0.05.

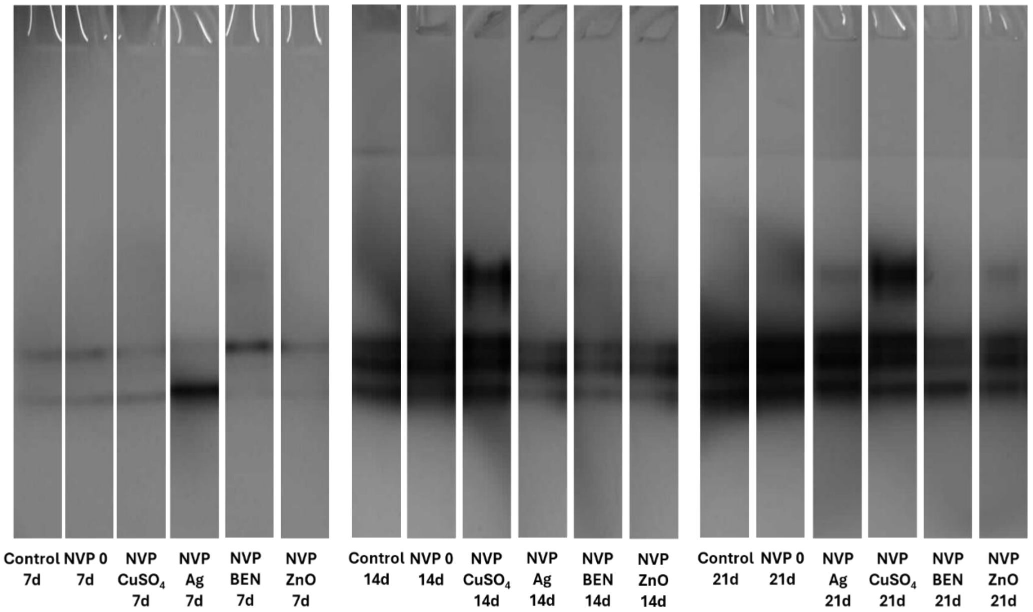

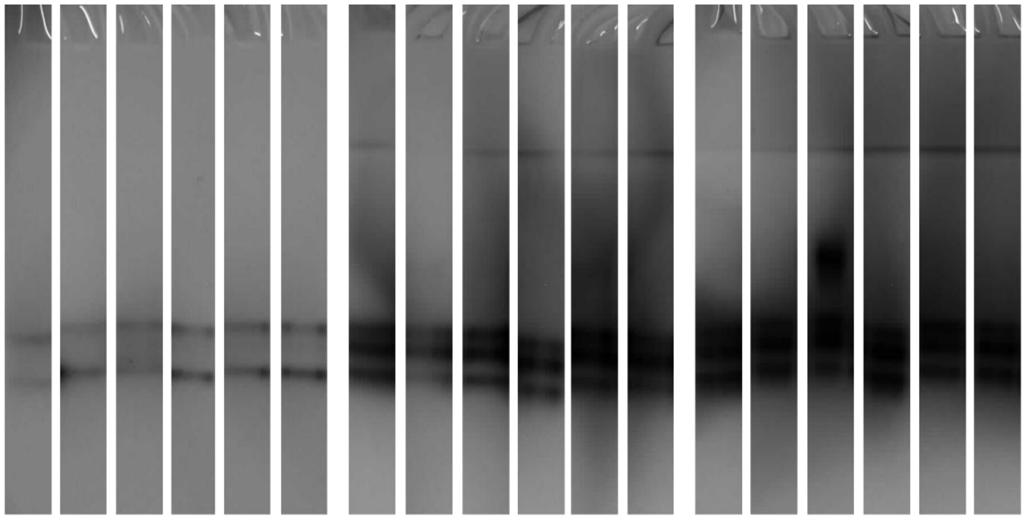


**Figure S13.** Zymographic detection of extracellular laccase activity in the culture fluid of *C. unicolor* after 7, 14 and 21 days of cultivation in the presence of NVP- and HEMA-containing composite material fragments, following UV sterilization

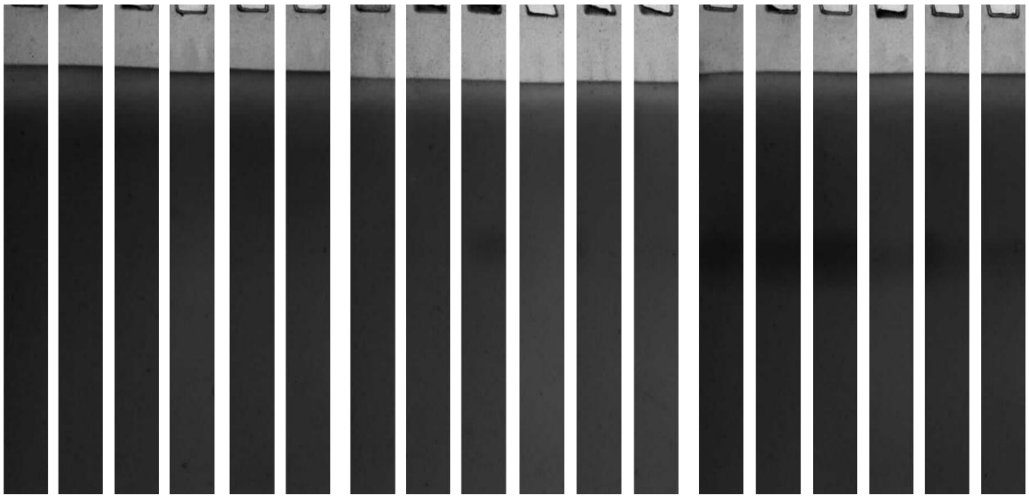

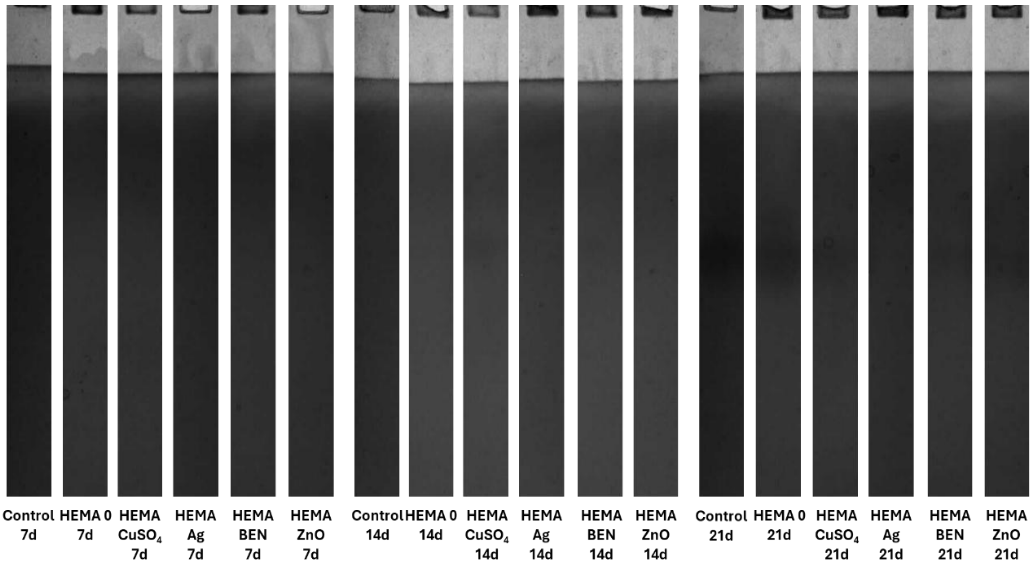


**Figure S14.** Electrophoretic detection of alkaline protease activity in the culture fluid of *C. unicolor* after 7, 14 and 21 days of cultivation in the presence of NVP- and HEMA-containing composite material fragments, following UV sterilization

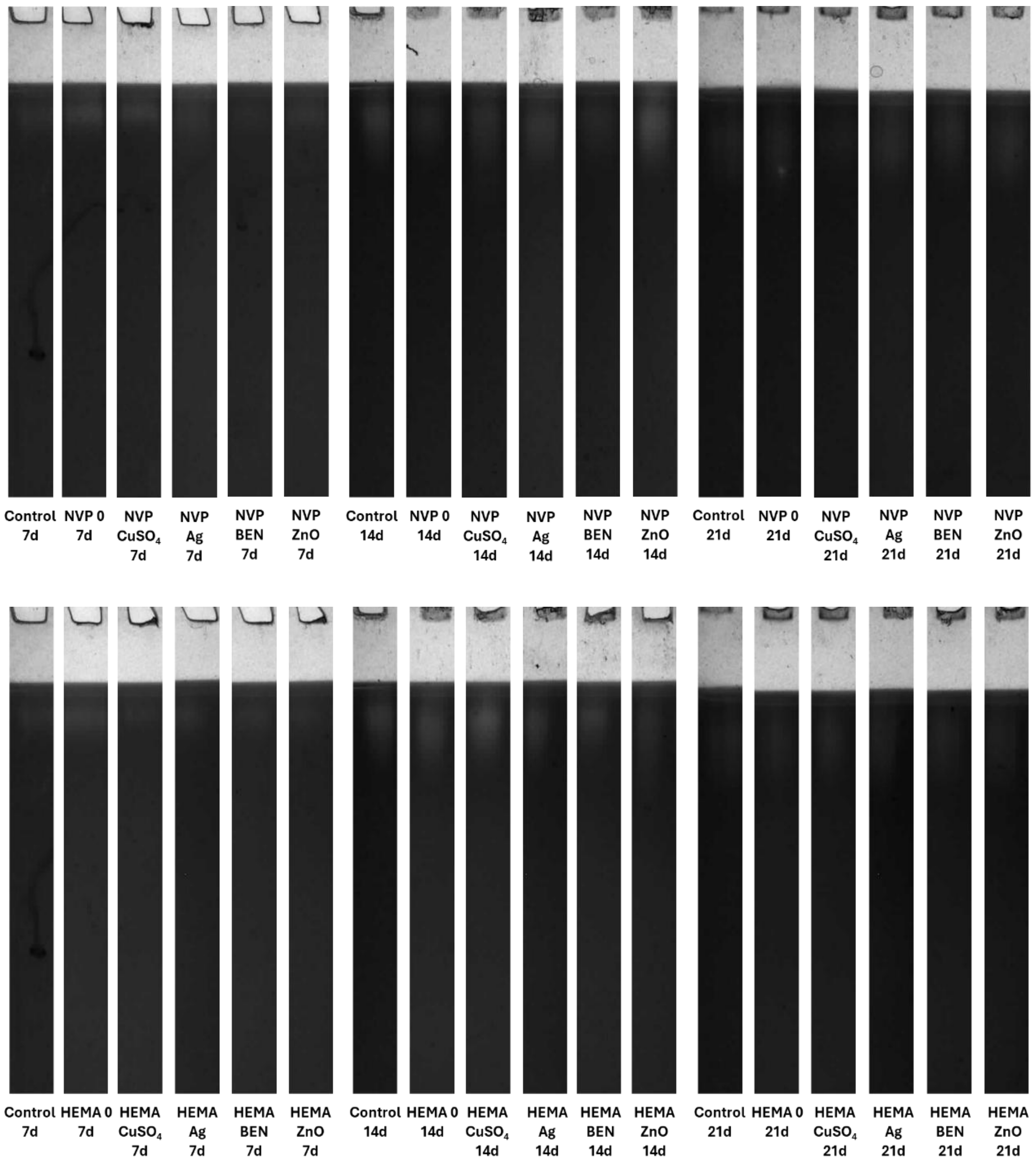


**Figure S15.** Zymographic detection of acid protease activity in the culture fluid of *C. unicolor* after 7, 14 and 21 days of cultivation in the presence of NVP- and HEMA-containing composite material fragments, following UV sterilization

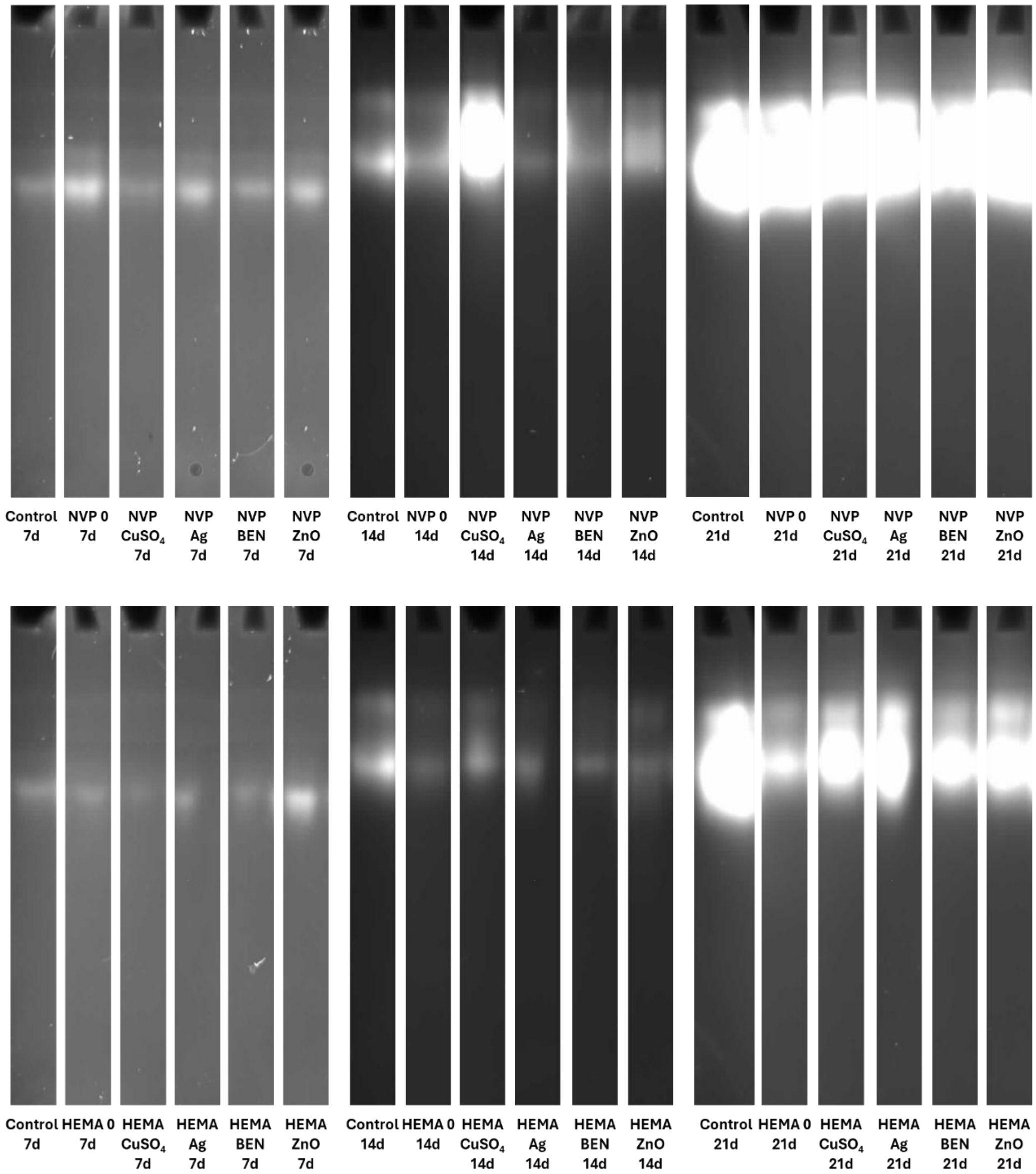


**Figure S16.** Electrophoretic detection of β-glucosidase activity in the culture fluid of *C. unicolor* after 7, 14 and 21 days of cultivation in the presence of NVP- and HEMA-containing composite material fragments, following UV sterilization
