# Supplementary figures and images for: New Cross-Linked Polymeric Materials Modified with Antimicrobial Compounds in Relation to Their Biological Activities and Biodegradation by the Laccase-Producing Fungus Cerrena unicolor
Source: Biomolecules. 2026 May 15;16(5):731. doi: 10.3390/biom16050731 (PMC13204337; doi:10.3390/biom16050731)

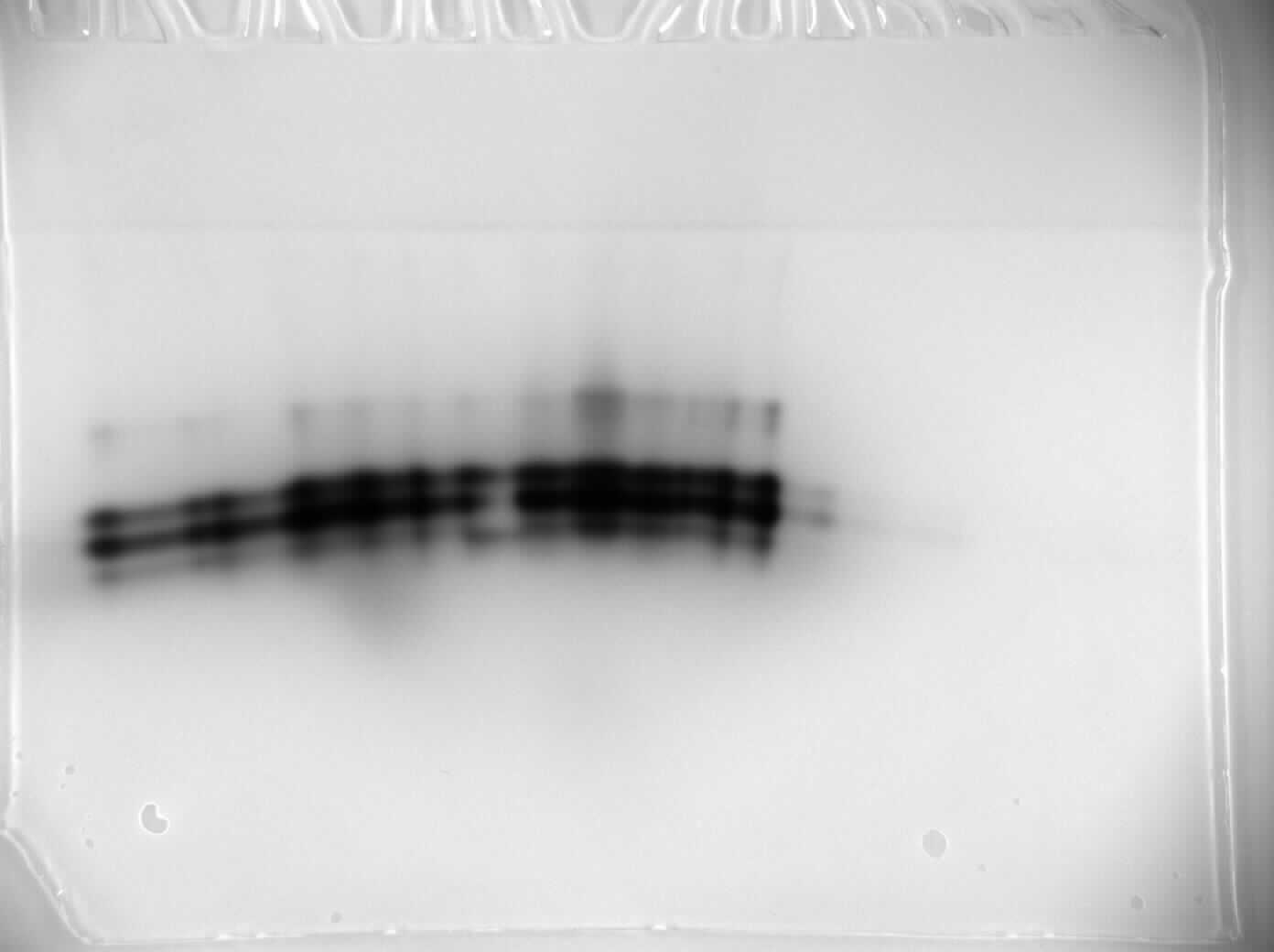

Supplement: Supplementary file 1 [file biomolecules-16-00731-s001.zip › File S1. original electrophoresis gel/Extracellular laccase activity/14 days after ethanol.jpg]

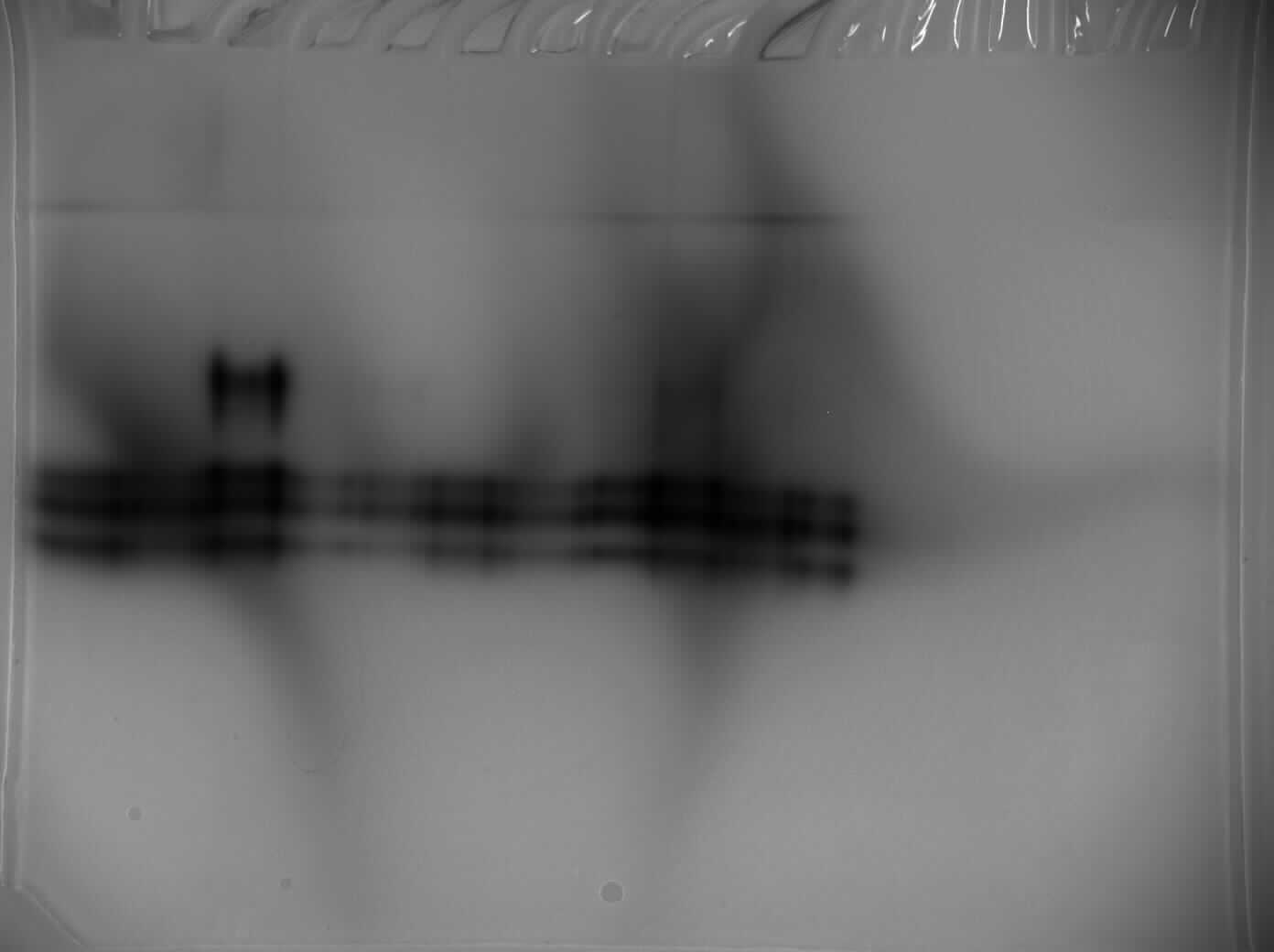

Supplement: Supplementary file 1 [file biomolecules-16-00731-s001.zip › File S1. original electrophoresis gel/Extracellular laccase activity/14 days after UV.jpg]

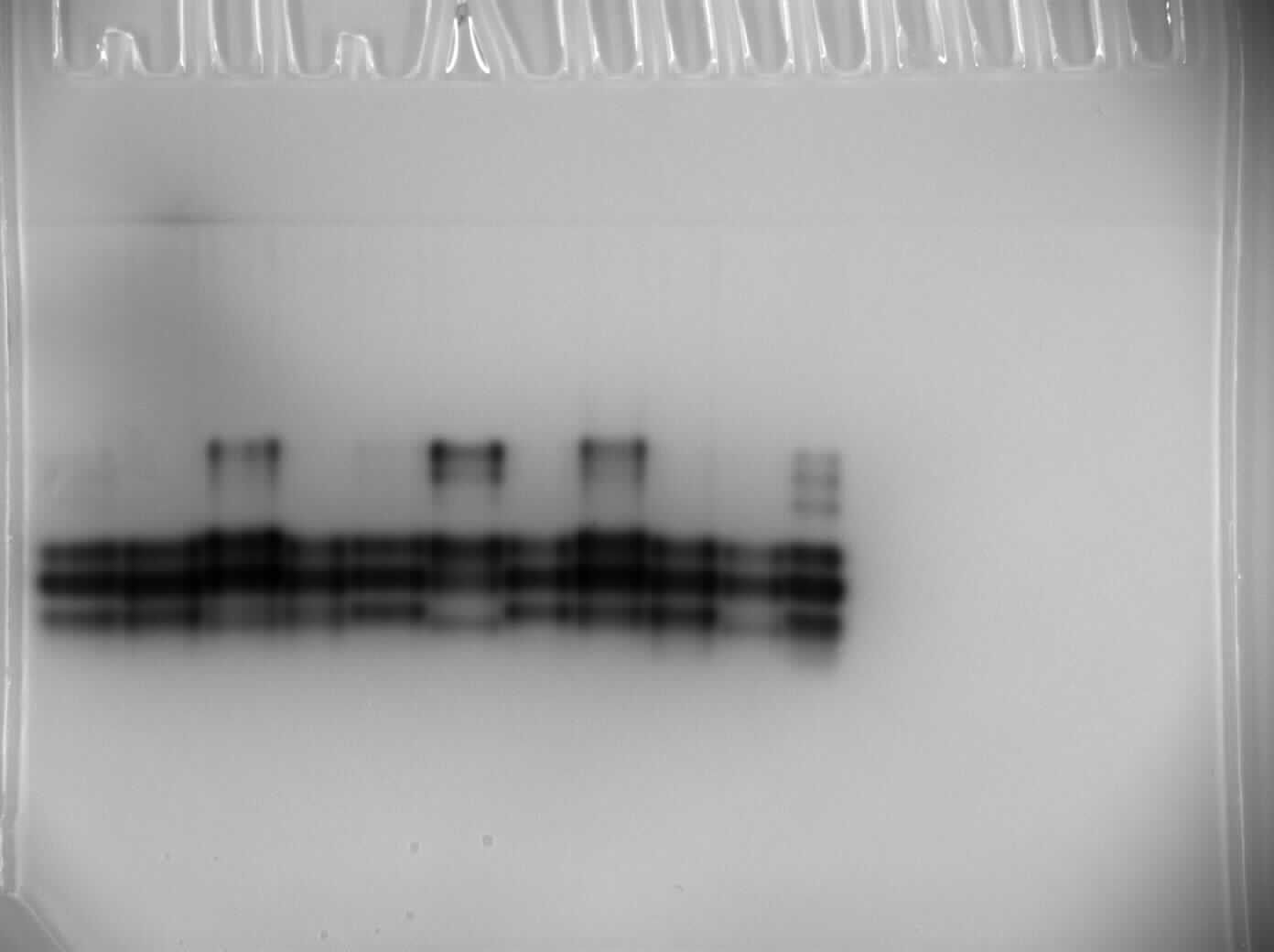

Supplement: Supplementary file 1 [file biomolecules-16-00731-s001.zip › File S1. original electrophoresis gel/Extracellular laccase activity/21 days after ehanol.jpg]

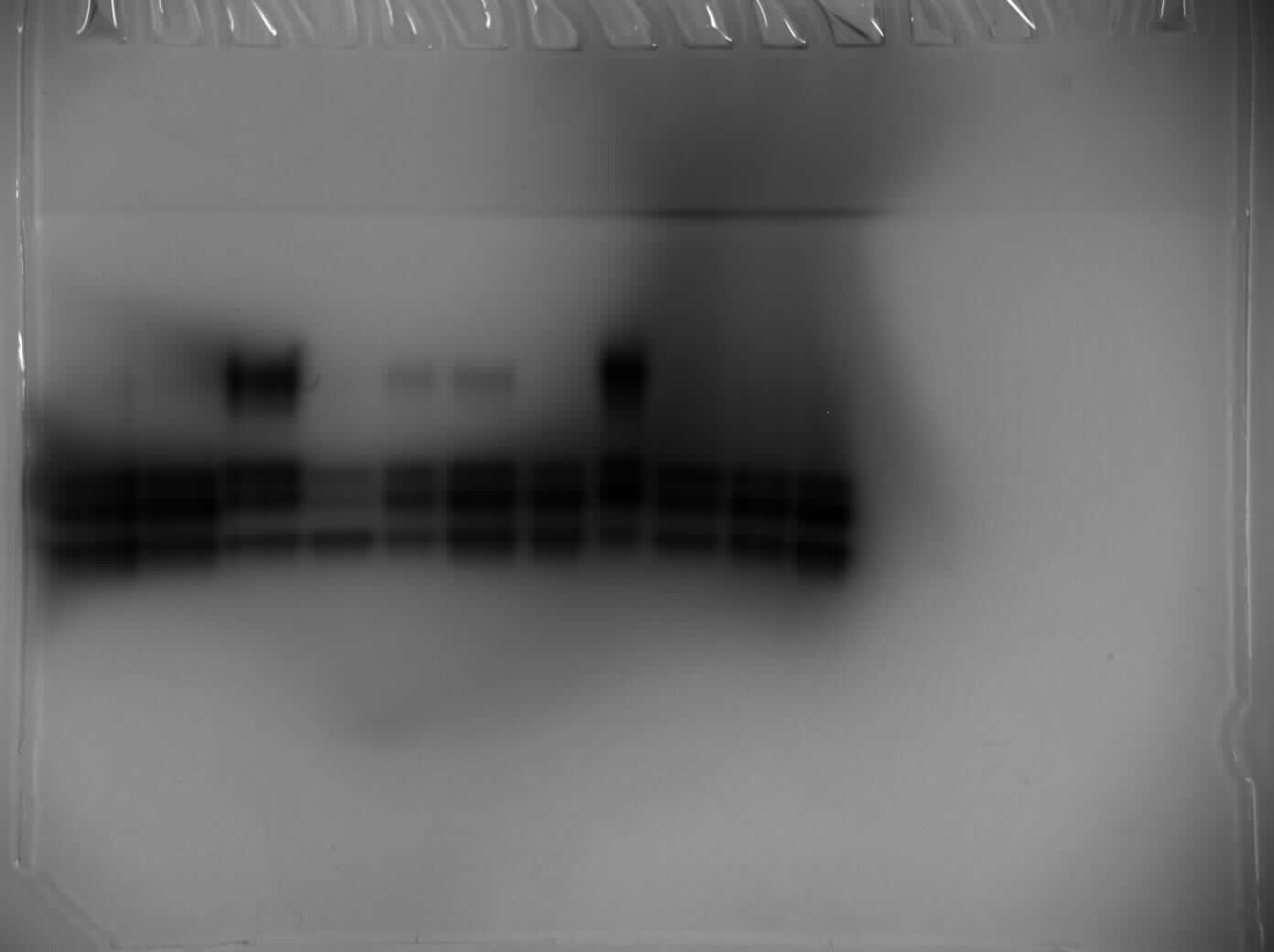

Supplement: Supplementary file 1 [file biomolecules-16-00731-s001.zip › File S1. original electrophoresis gel/Extracellular laccase activity/21 days after UV.jpg]

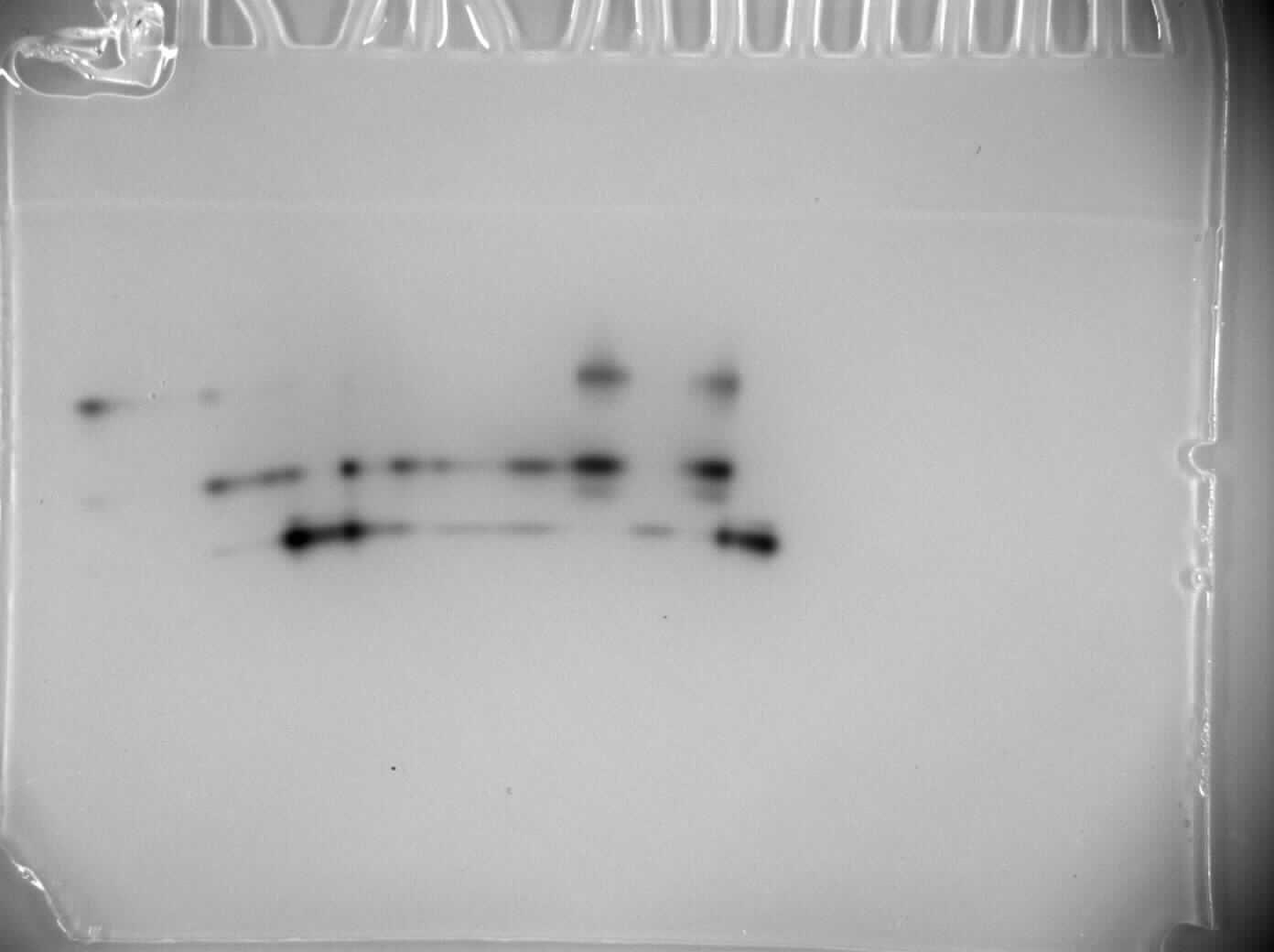

Supplement: Supplementary file 1 [file biomolecules-16-00731-s001.zip › File S1. original electrophoresis gel/Extracellular laccase activity/7 days after ethanol.jpg]

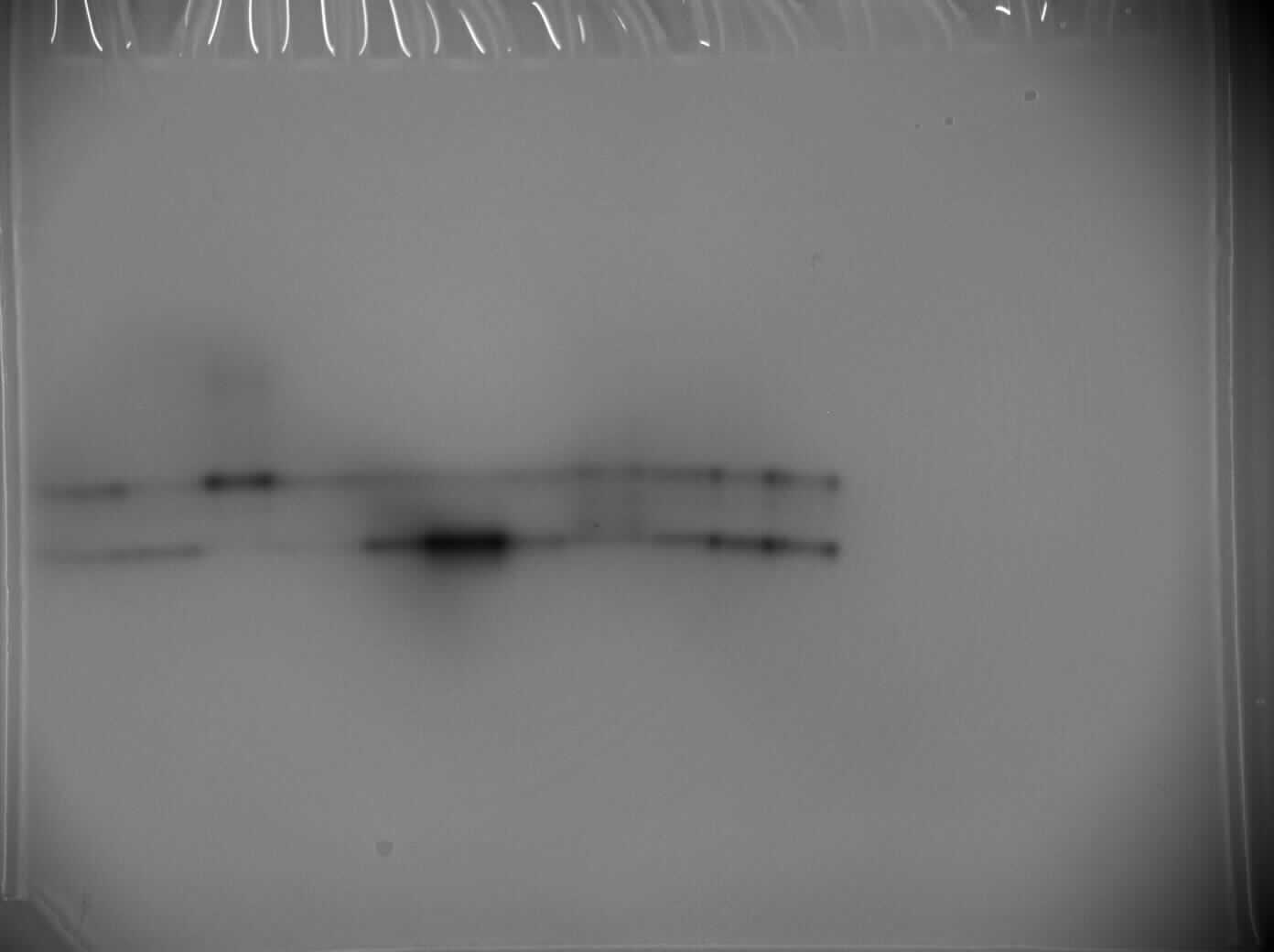

Supplement: Supplementary file 1 [file biomolecules-16-00731-s001.zip › File S1. original electrophoresis gel/Extracellular laccase activity/7days after UV.jpg]

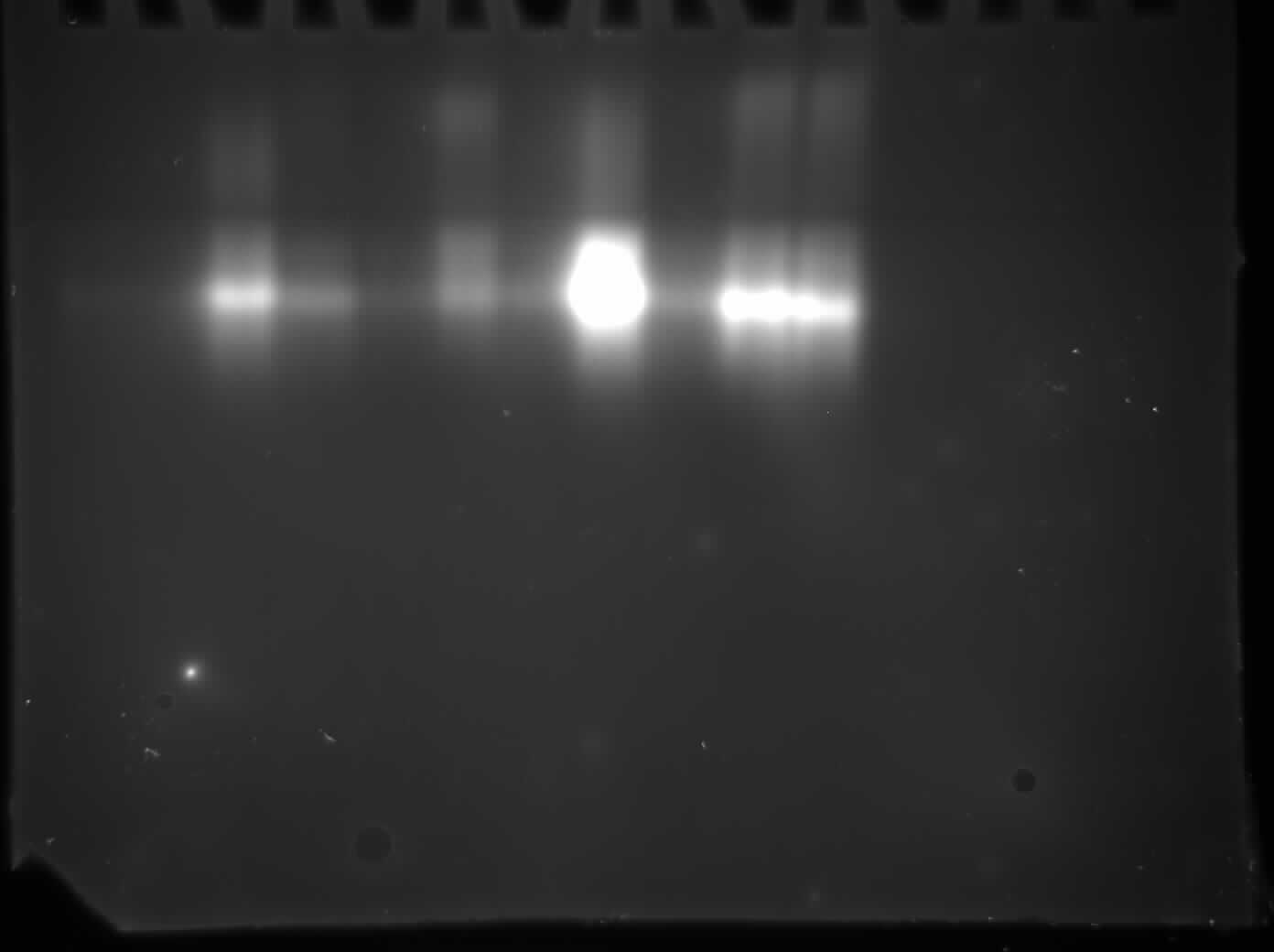

Supplement: Supplementary file 1 [file biomolecules-16-00731-s001.zip › File S1. original electrophoresis gel/glucosidase/14 days after ethanol.jpg]

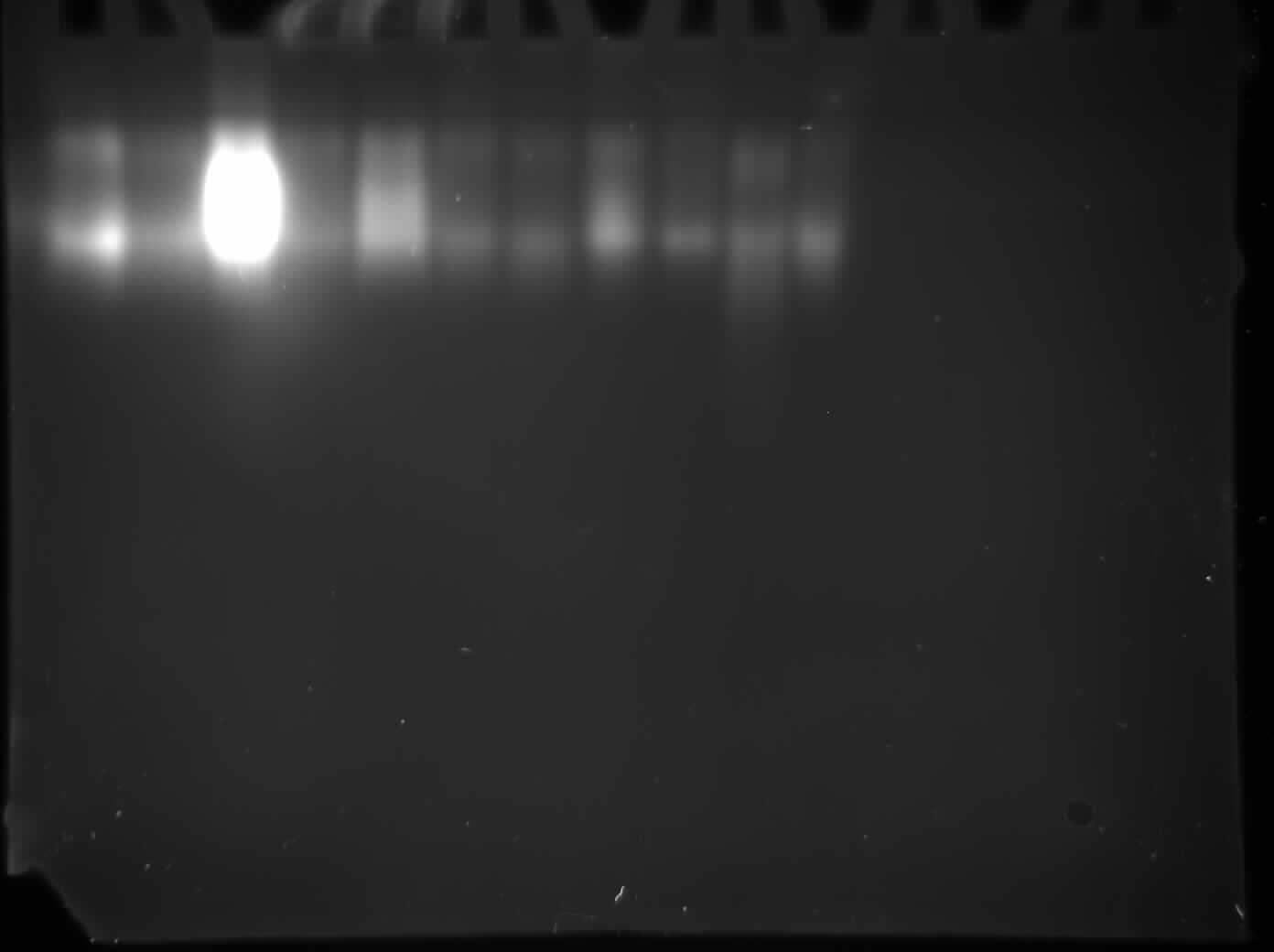

Supplement: Supplementary file 1 [file biomolecules-16-00731-s001.zip › File S1. original electrophoresis gel/glucosidase/14 days after UV.jpg]

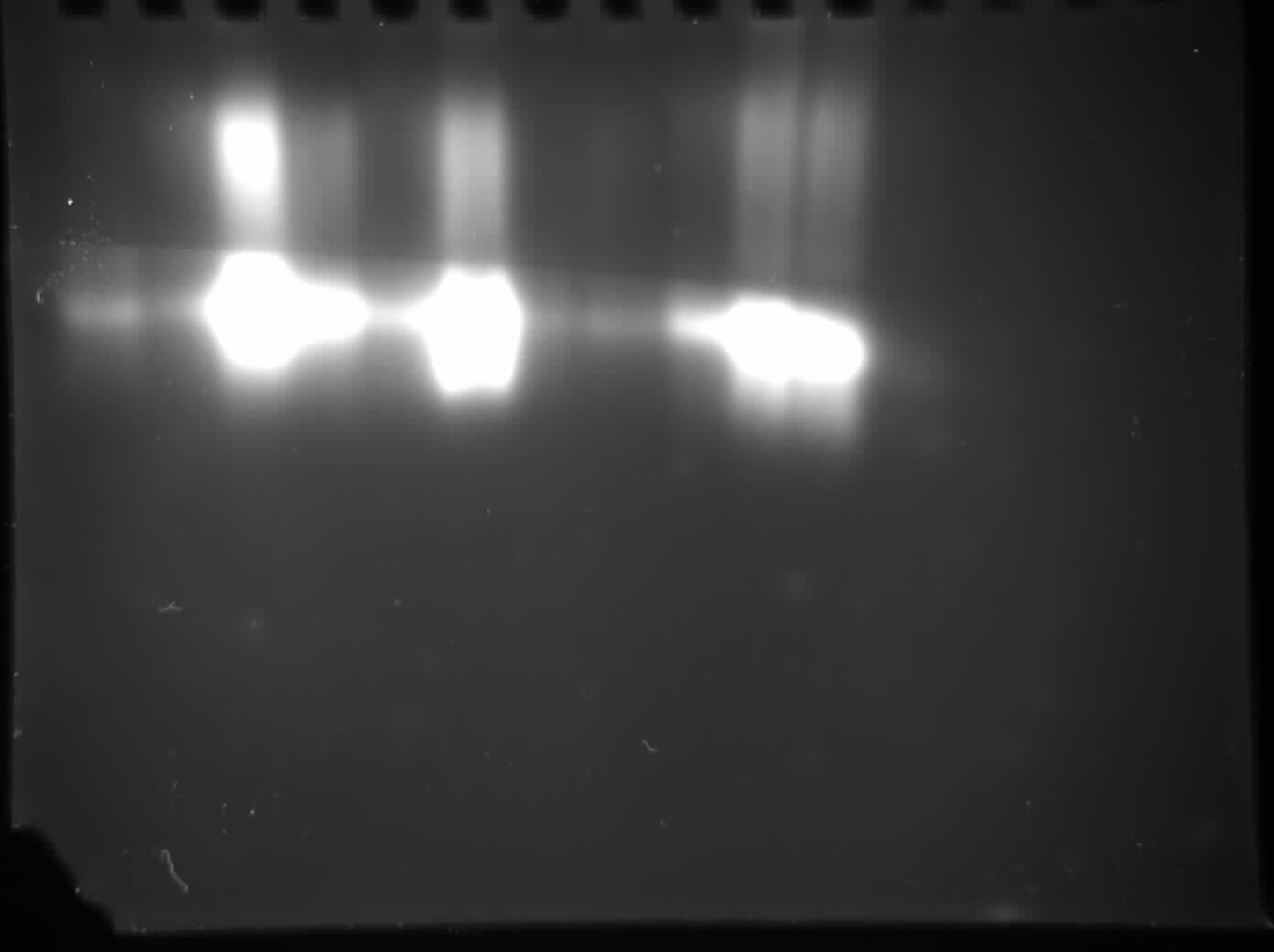

Supplement: Supplementary file 1 [file biomolecules-16-00731-s001.zip › File S1. original electrophoresis gel/glucosidase/21 days after ethanol.jpg]

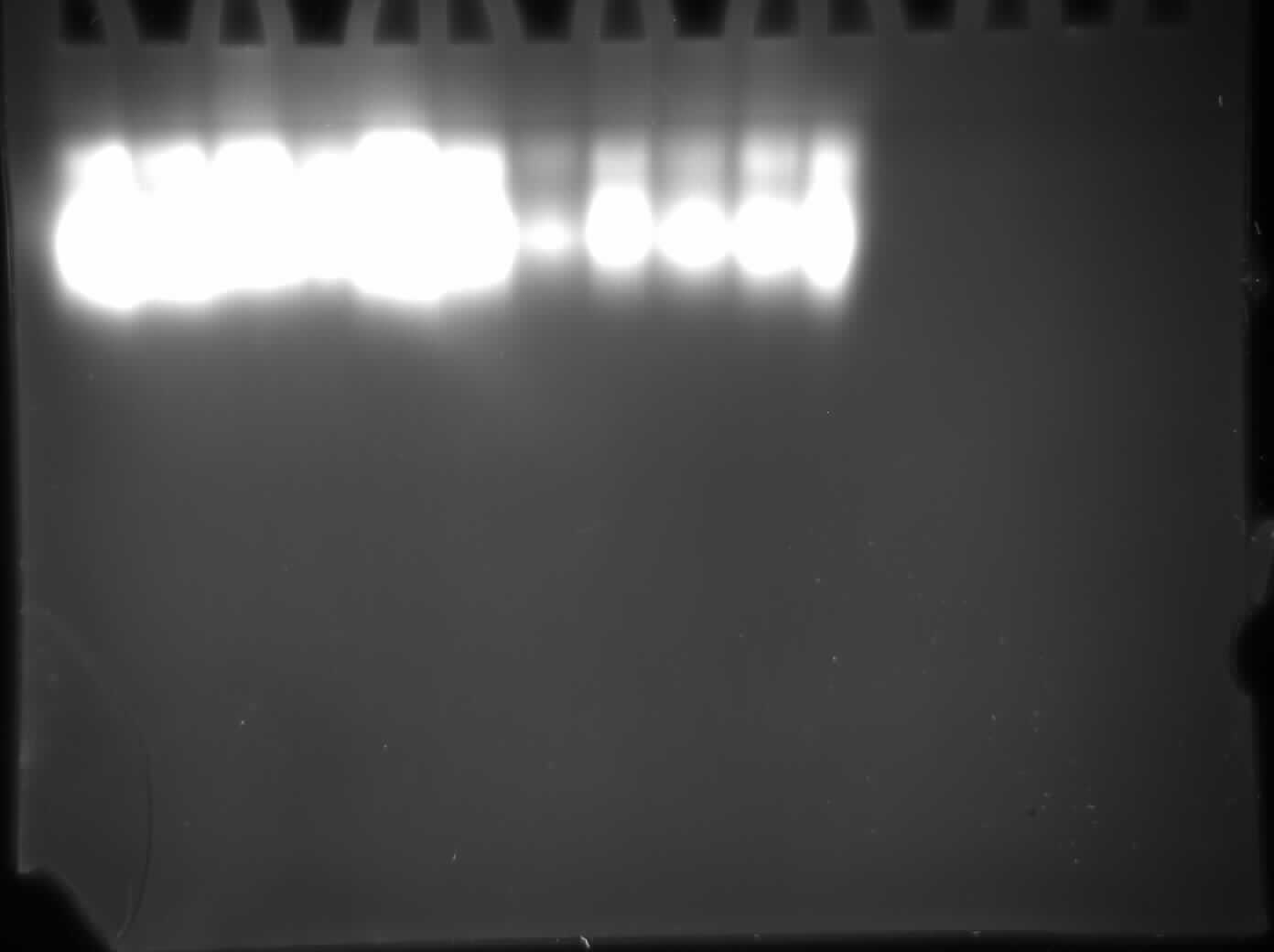

Supplement: Supplementary file 1 [file biomolecules-16-00731-s001.zip › File S1. original electrophoresis gel/glucosidase/21 days after UV.jpg]

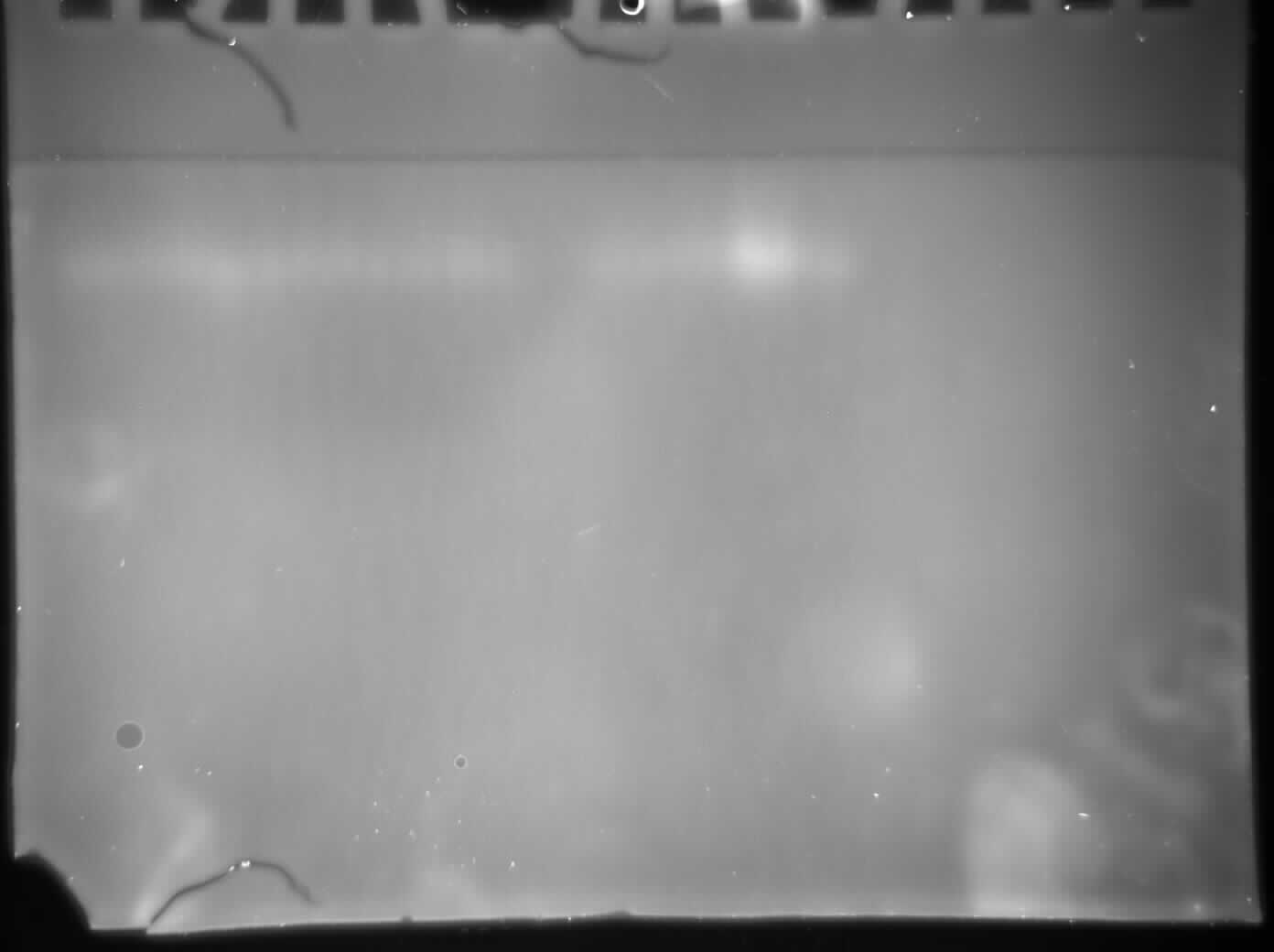

Supplement: Supplementary file 1 [file biomolecules-16-00731-s001.zip › File S1. original electrophoresis gel/glucosidase/7 days after ethanol.jpg]

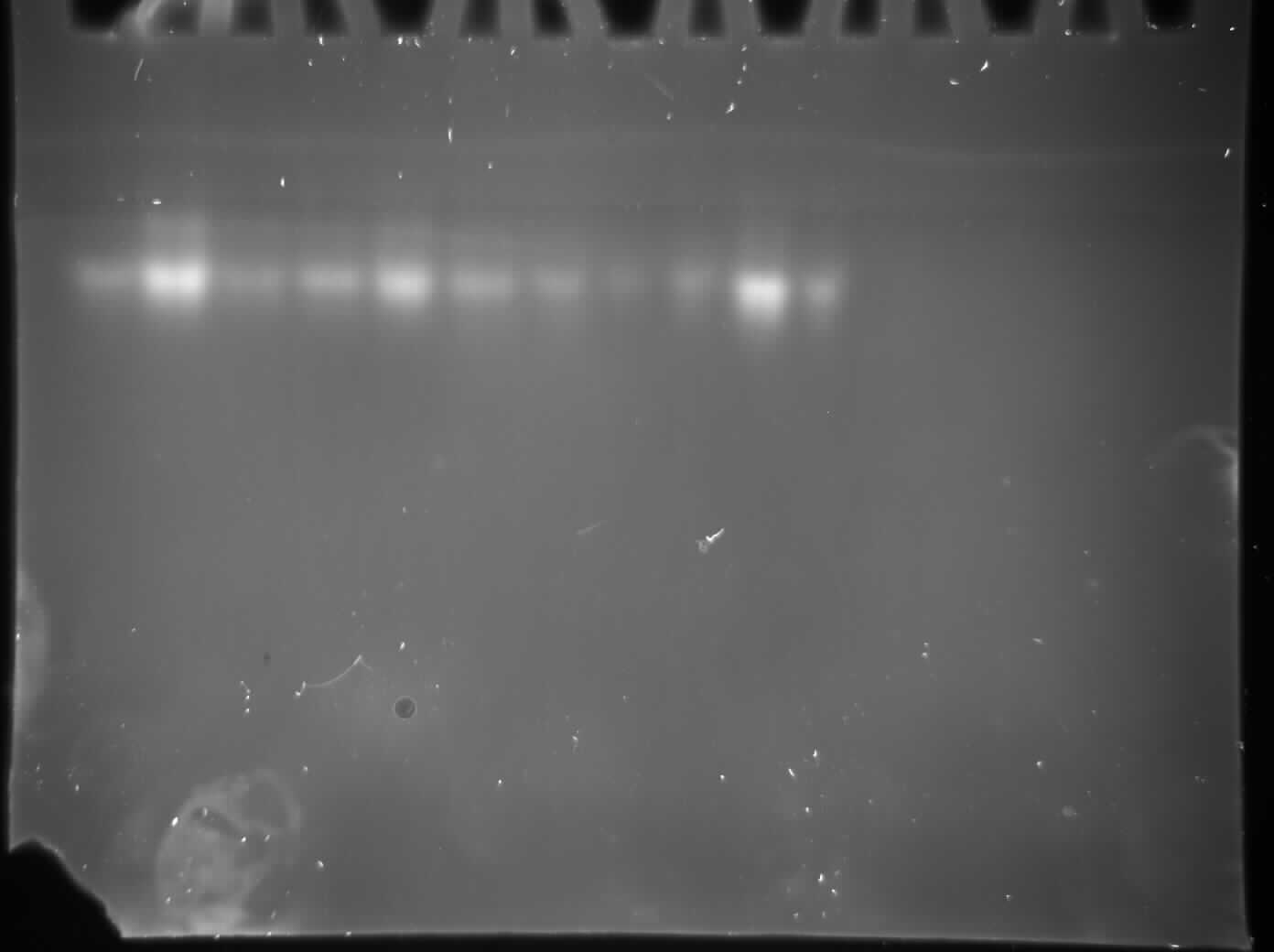

Supplement: Supplementary file 1 [file biomolecules-16-00731-s001.zip › File S1. original electrophoresis gel/glucosidase/7 days after UV.jpg]

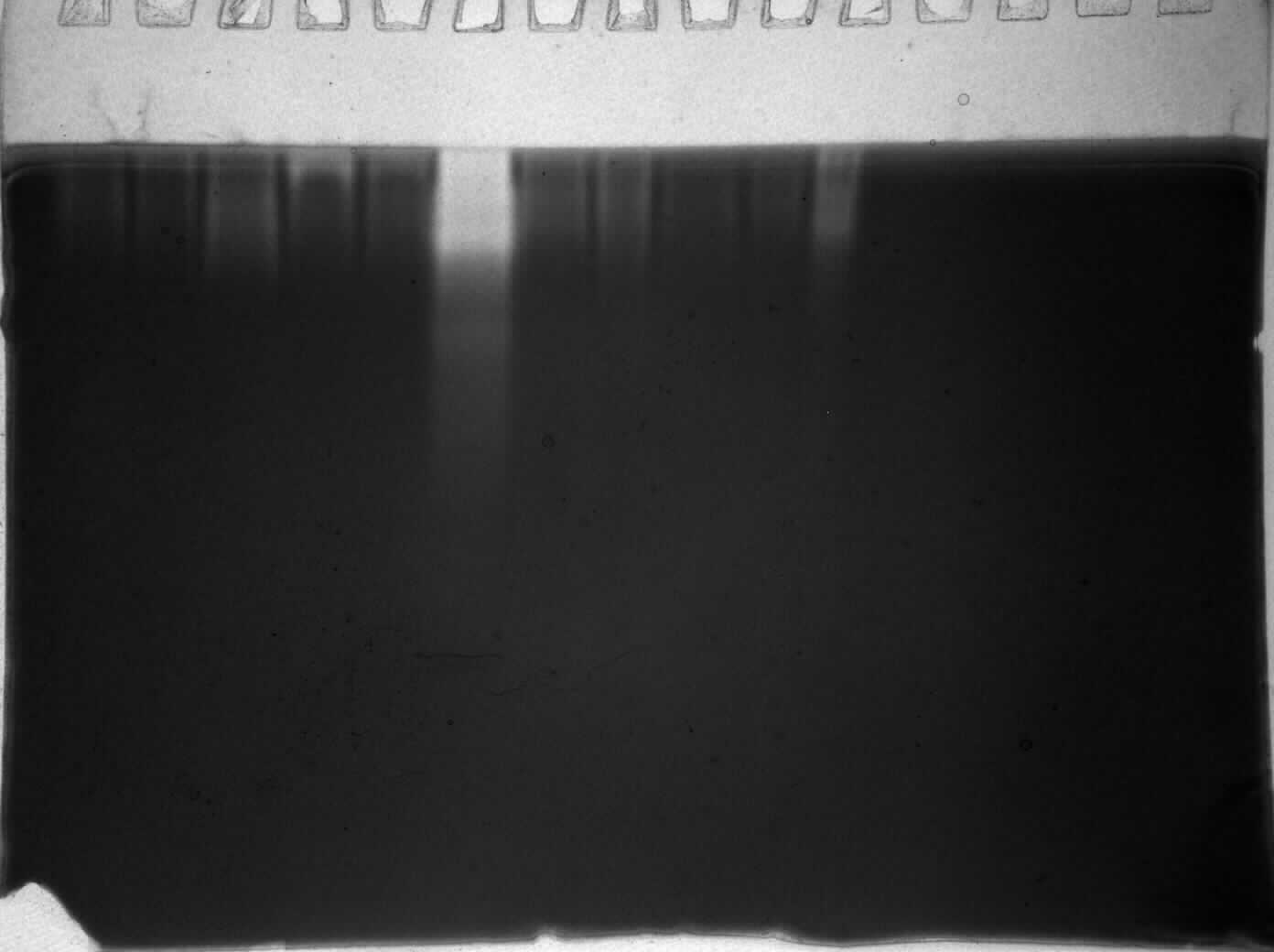

Supplement: Supplementary file 1 [file biomolecules-16-00731-s001.zip › File S1. original electrophoresis gel/Protease/ethanol 3.5/14 days ethanol ph 3.5.jpg]

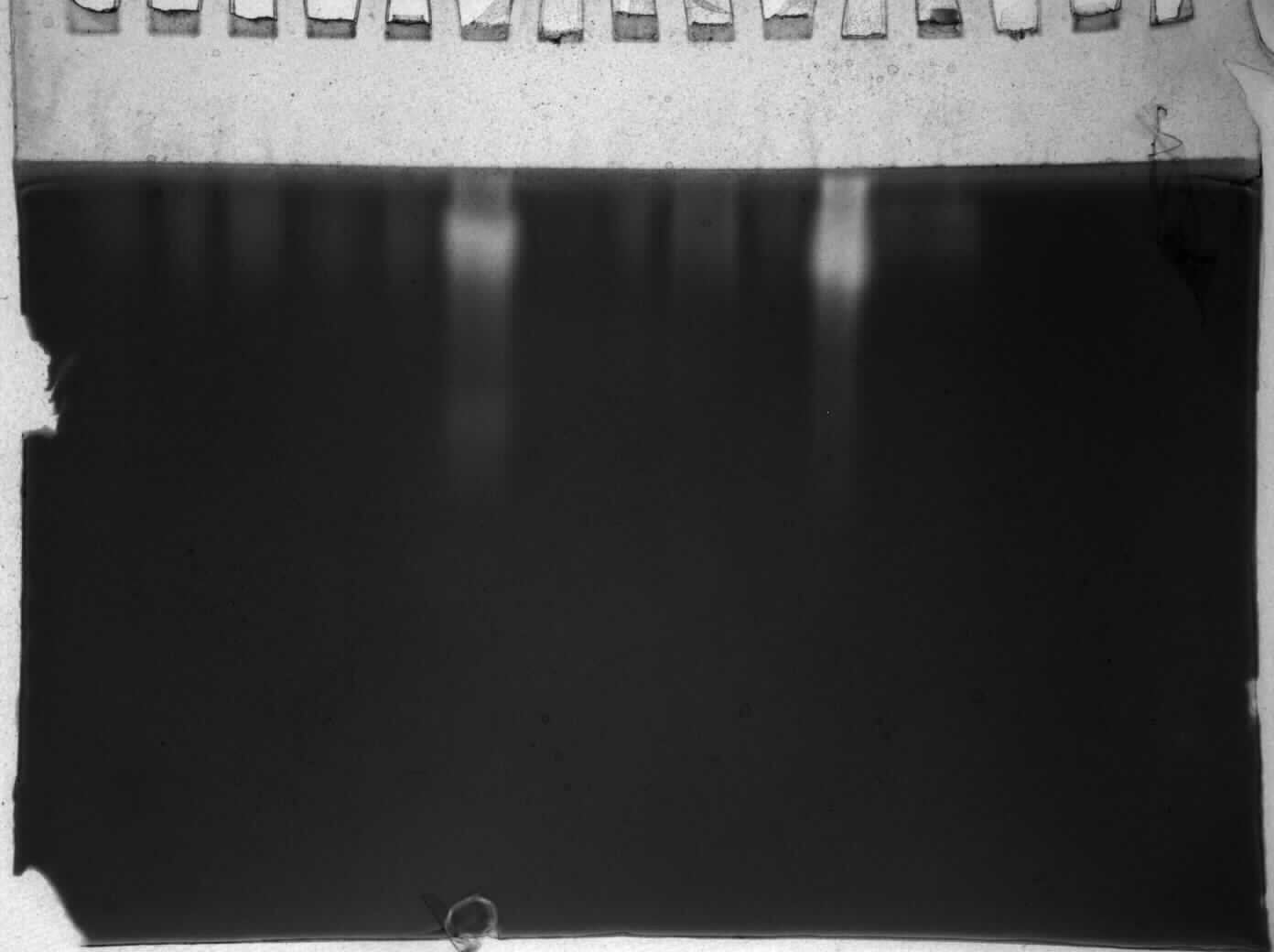

Supplement: Supplementary file 1 [file biomolecules-16-00731-s001.zip › File S1. original electrophoresis gel/Protease/ethanol 3.5/21 days ethanol ph 3.5.jpg]

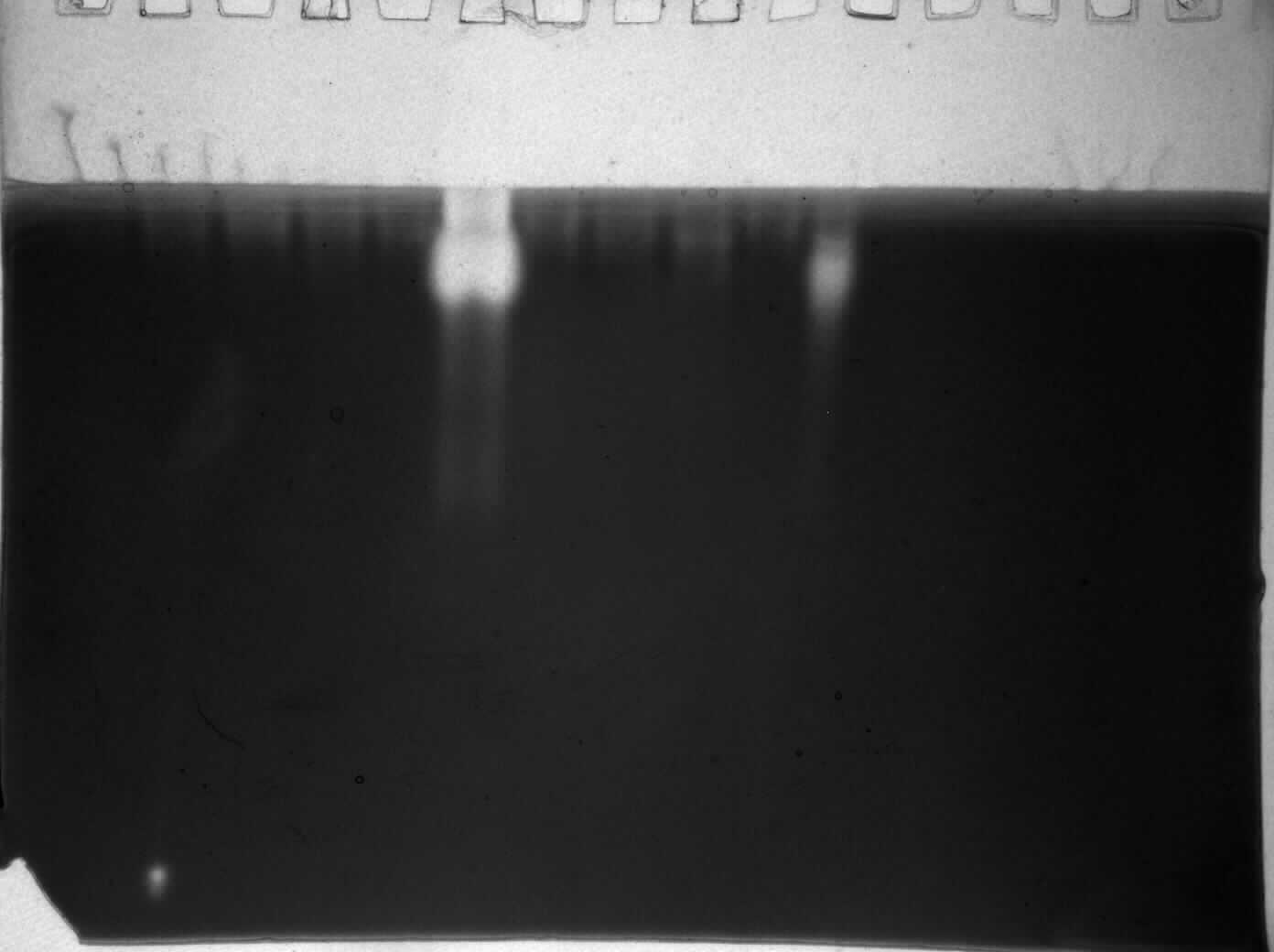

Supplement: Supplementary file 1 [file biomolecules-16-00731-s001.zip › File S1. original electrophoresis gel/Protease/ethanol 3.5/7 days ethanol ph 3.5.jpg]

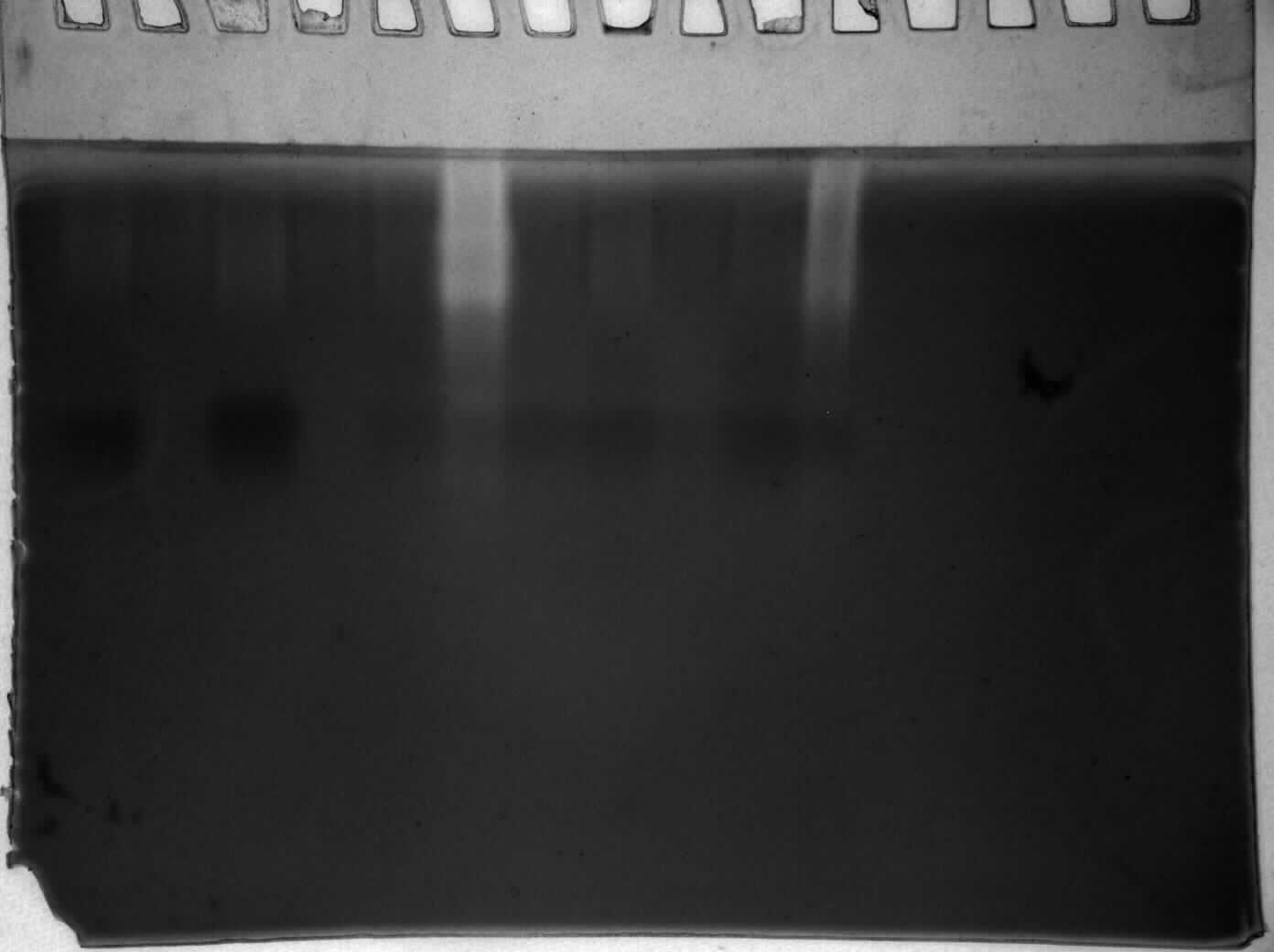

Supplement: Supplementary file 1 [file biomolecules-16-00731-s001.zip › File S1. original electrophoresis gel/Protease/ethanol pH 8/14 days etanol ph 8.0.jpg]

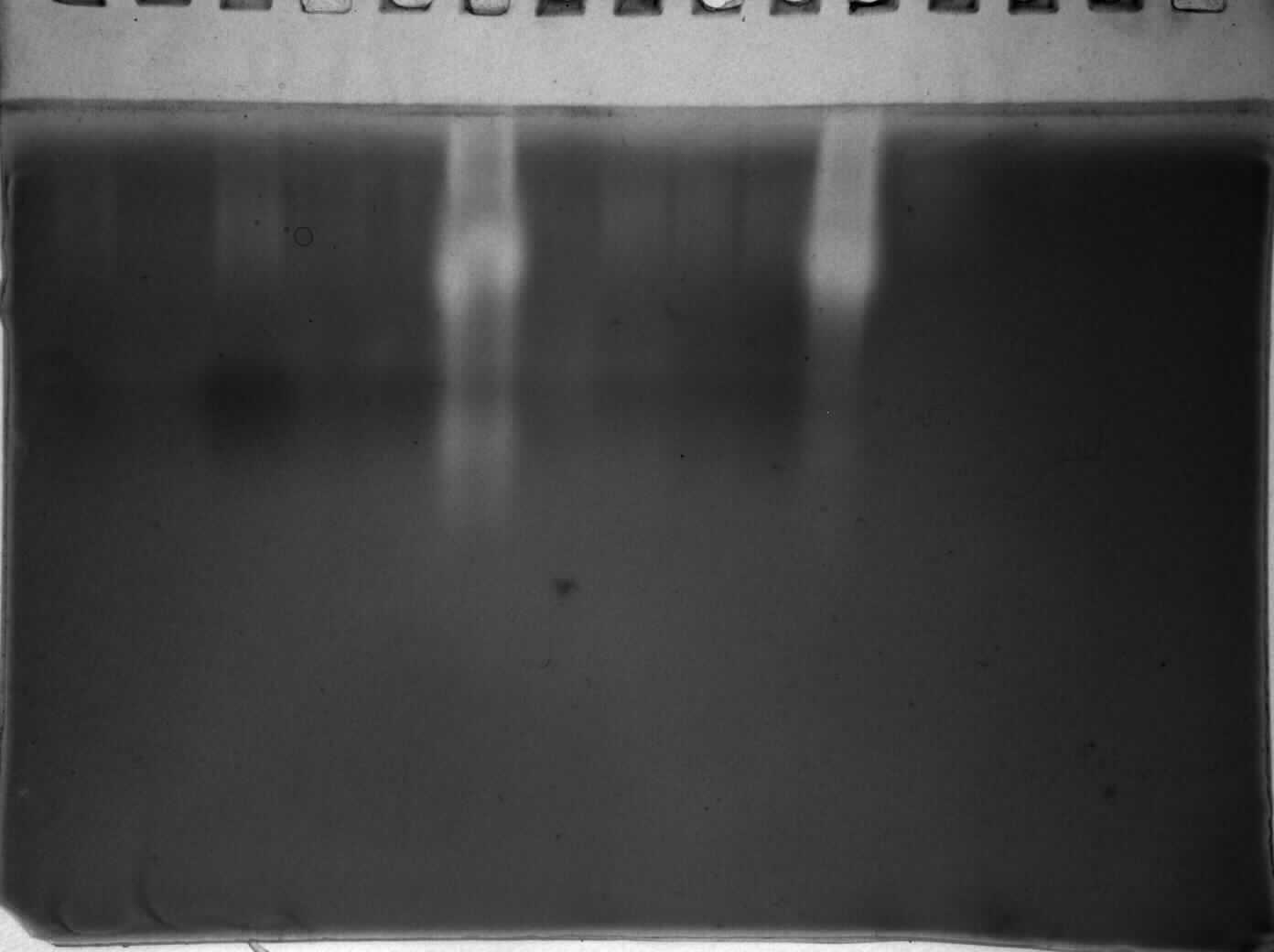

Supplement: Supplementary file 1 [file biomolecules-16-00731-s001.zip › File S1. original electrophoresis gel/Protease/ethanol pH 8/21 days etanol ph 8.0.jpg]

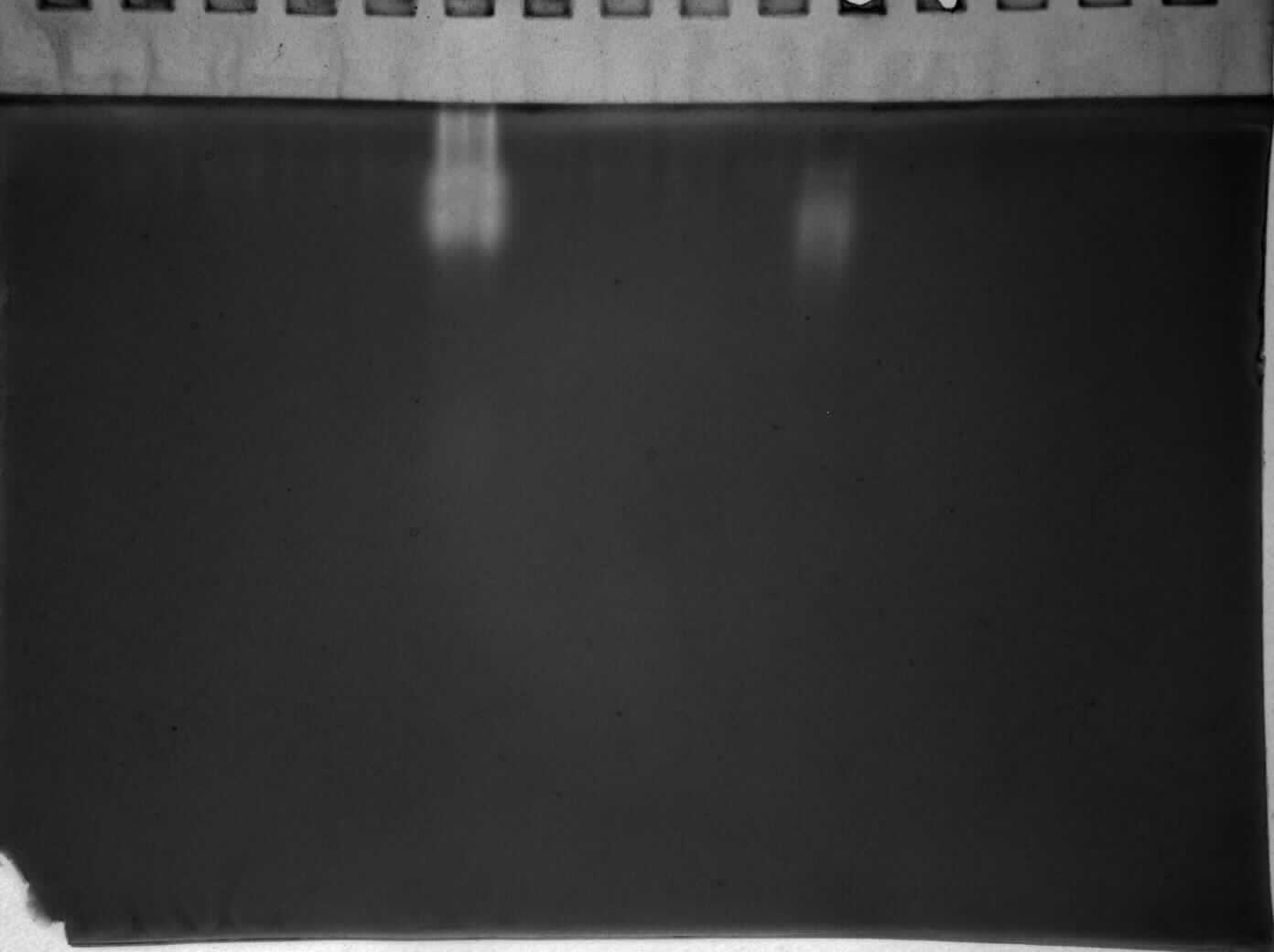

Supplement: Supplementary file 1 [file biomolecules-16-00731-s001.zip › File S1. original electrophoresis gel/Protease/ethanol pH 8/7 days ethanol ph 8.0.jpg]

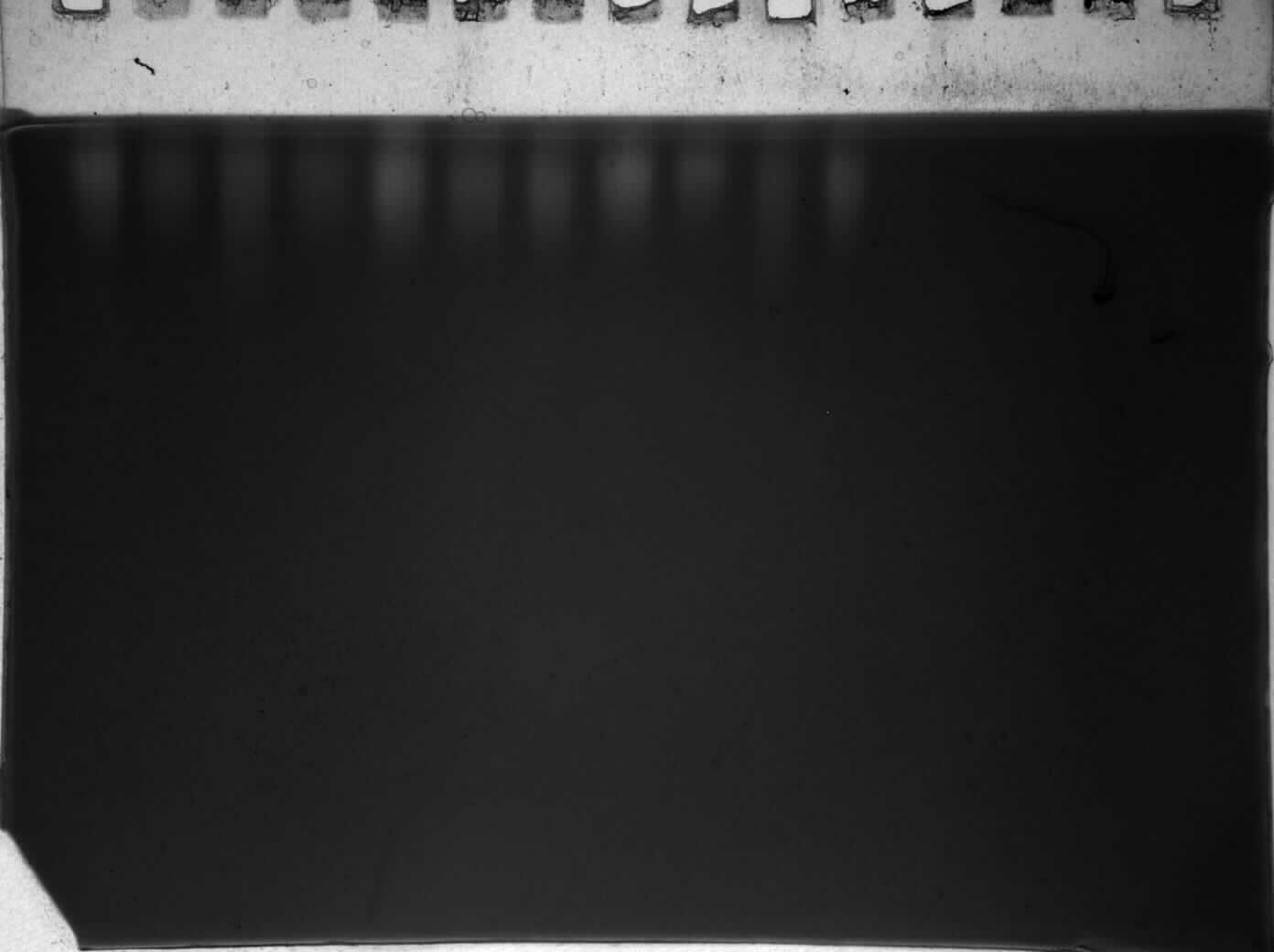

Supplement: Supplementary file 1 [file biomolecules-16-00731-s001.zip › File S1. original electrophoresis gel/Protease/UV pH 3.5/14 days uv ph 3.5.jpg]

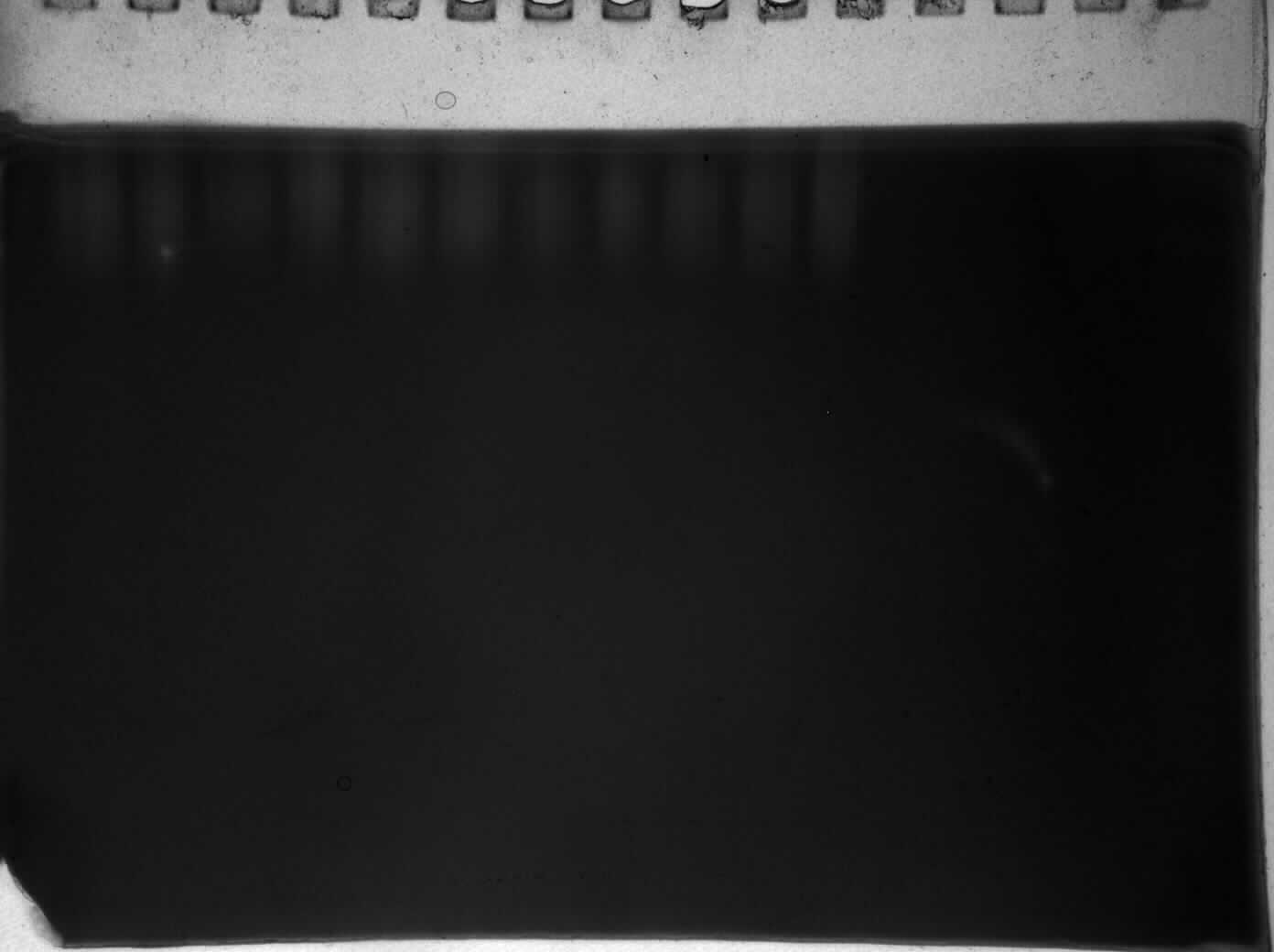

Supplement: Supplementary file 1 [file biomolecules-16-00731-s001.zip › File S1. original electrophoresis gel/Protease/UV pH 3.5/21 days uv ph 3.5.jpg]

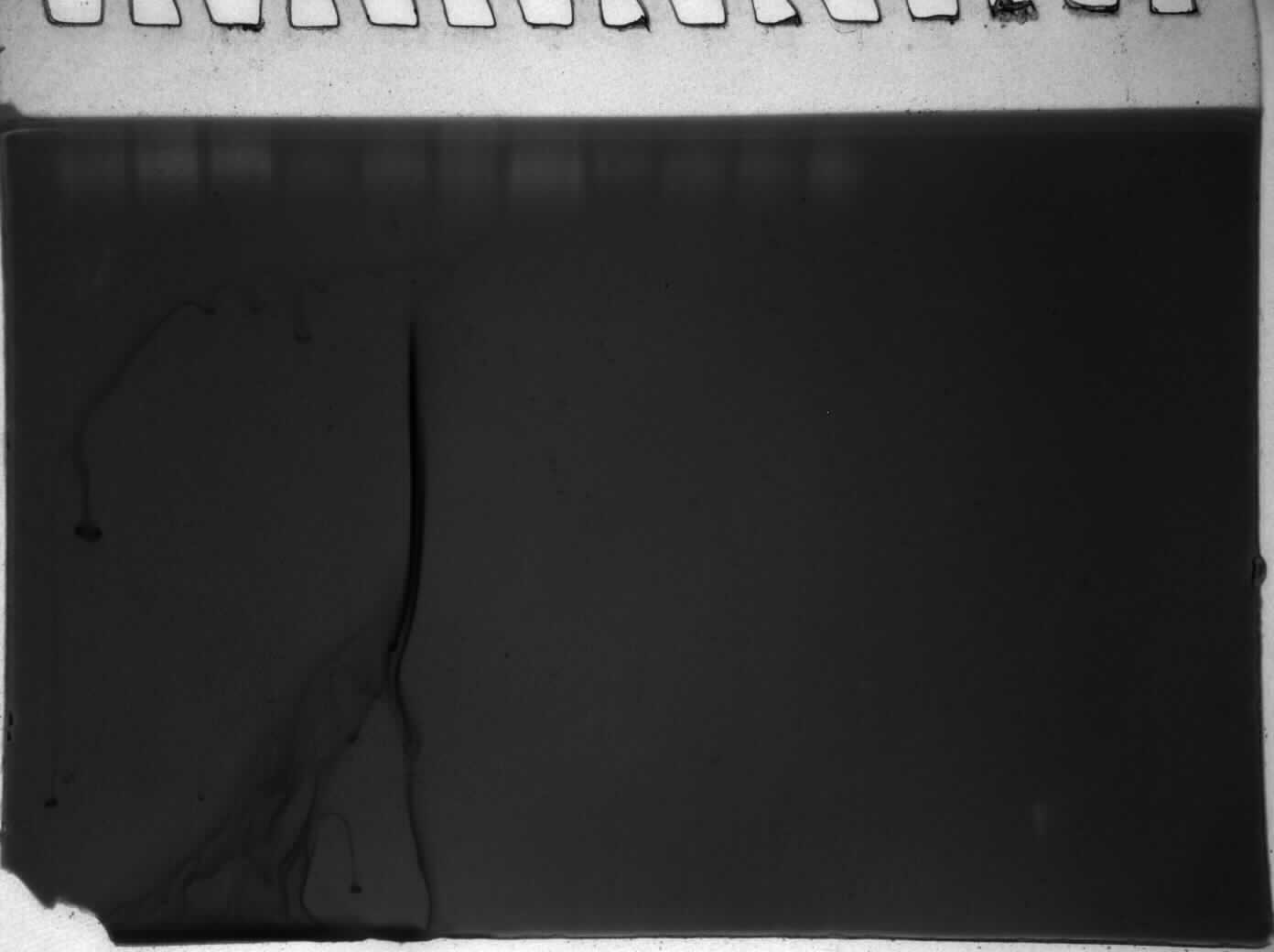

Supplement: Supplementary file 1 [file biomolecules-16-00731-s001.zip › File S1. original electrophoresis gel/Protease/UV pH 3.5/7 days uv ph 3.5.jpg]

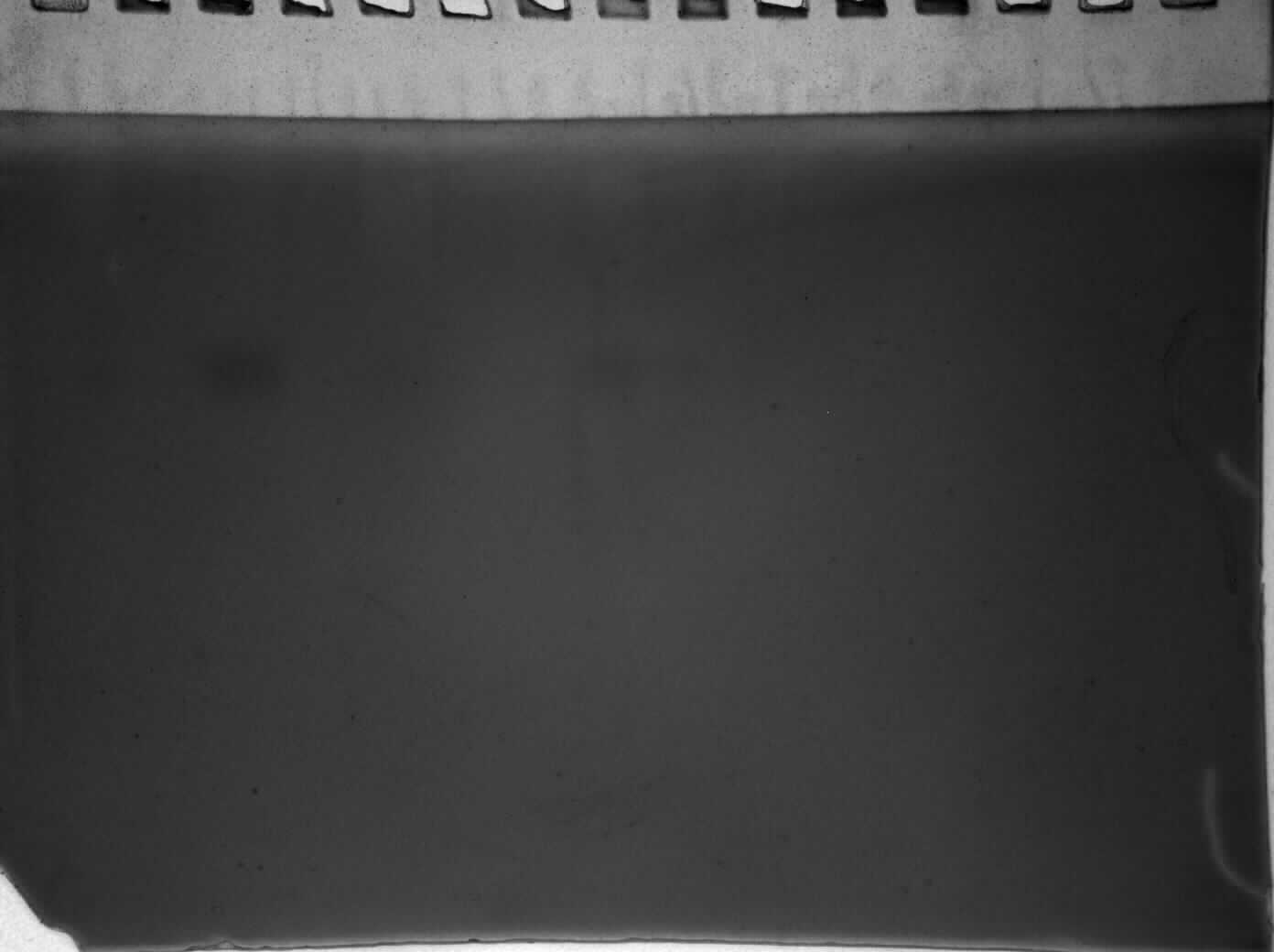

Supplement: Supplementary file 1 [file biomolecules-16-00731-s001.zip › File S1. original electrophoresis gel/Protease/UV pH 8/14 days uv ph 8.0.jpg]

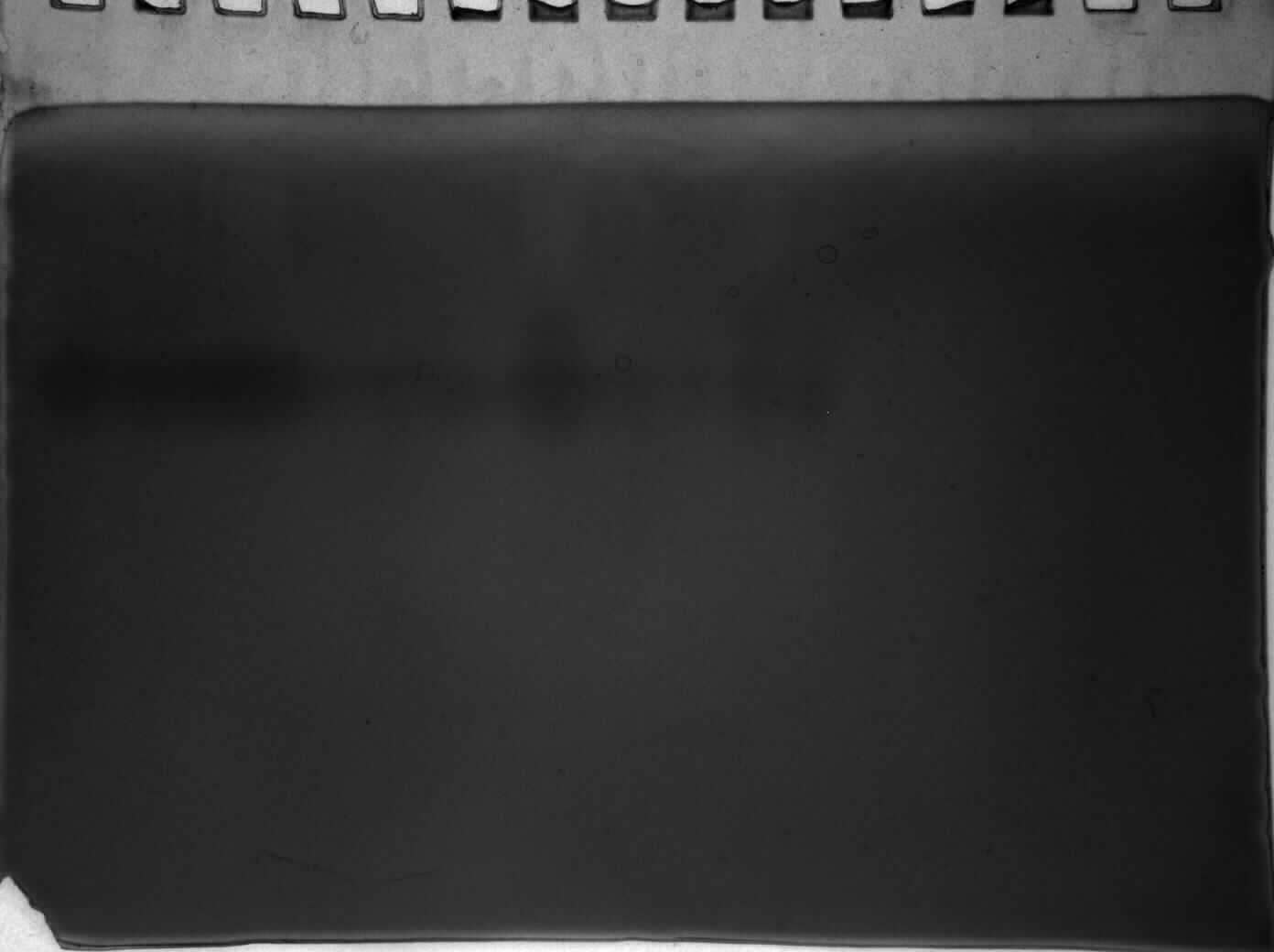

Supplement: Supplementary file 1 [file biomolecules-16-00731-s001.zip › File S1. original electrophoresis gel/Protease/UV pH 8/21 days uv ph 8.0.jpg]

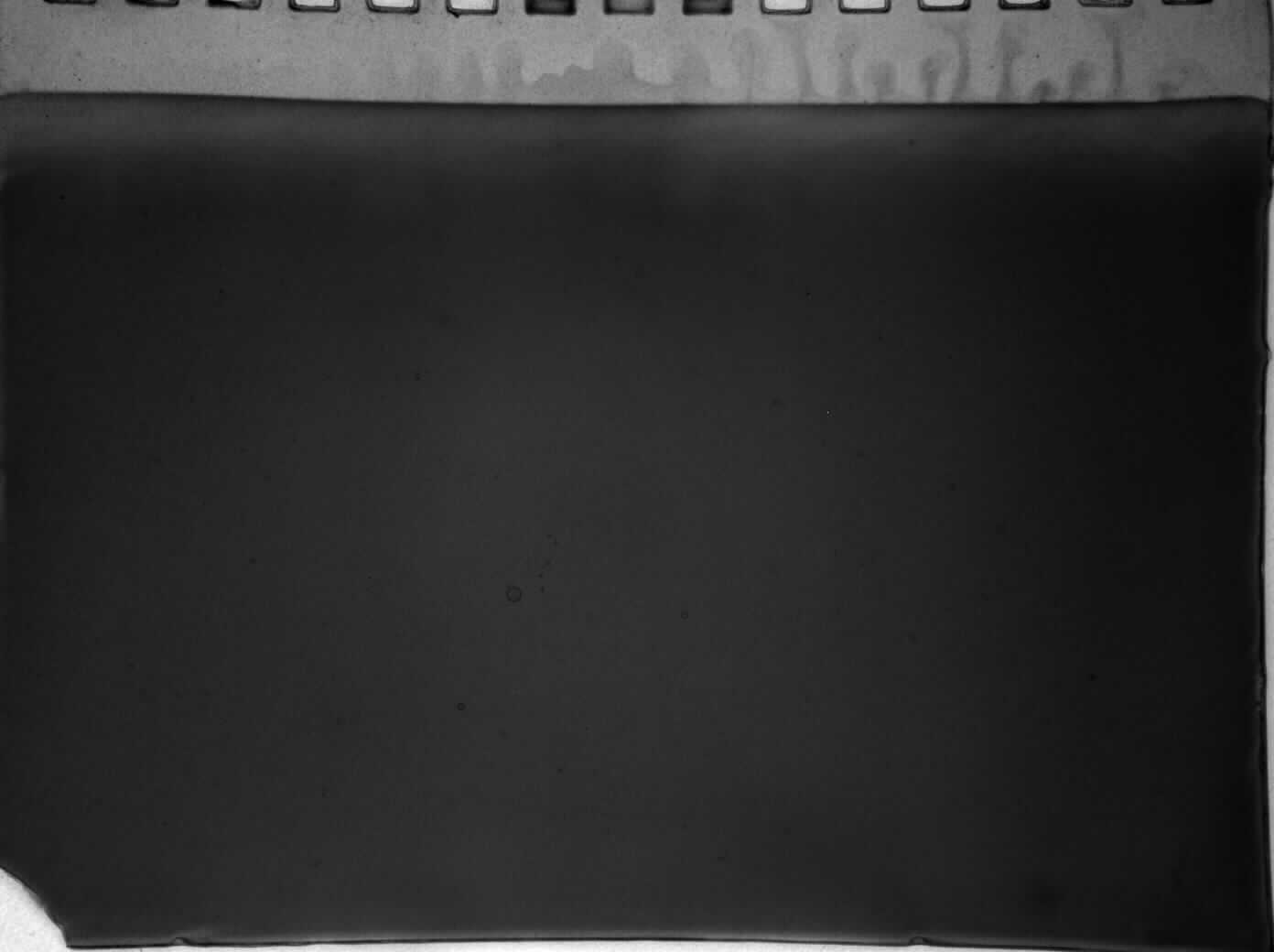

Supplement: Supplementary file 1 [file biomolecules-16-00731-s001.zip › File S1. original electrophoresis gel/Protease/UV pH 8/7 days uv ph 8.0.jpg]
